# Six reference-quality genomes reveal evolution of bat adaptations

**DOI:** 10.1038/s41586-020-2486-3

**Published:** 2020-07-22

**Authors:** David Jebb, Zixia Huang, Martin Pippel, Graham M. Hughes, Ksenia Lavrichenko, Paolo Devanna, Sylke Winkler, Lars S. Jermiin, Emilia C. Skirmuntt, Aris Katzourakis, Lucy Burkitt-Gray, David A. Ray, Kevin A. M. Sullivan, Juliana G. Roscito, Bogdan M. Kirilenko, Liliana M. Dávalos, Angelique P. Corthals, Megan L. Power, Gareth Jones, Roger D. Ransome, Dina K. N. Dechmann, Andrea G. Locatelli, Sébastien J. Puechmaille, Olivier Fedrigo, Erich D. Jarvis, Michael Hiller, Sonja C. Vernes, Eugene W. Myers, Emma C. Teeling

**Affiliations:** 1 Max Planck Institute of Molecular Cell Biology and Genetics, Dresden, Germany; 2 Max Planck Institute for the Physics of Complex Systems, Dresden, Germany; 3 Center for Systems Biology Dresden, Dresden, Germany; 4 School of Biology and Environmental Science, University College Dublin, Dublin, Ireland; 5 Neurogenetics of Vocal Communication Group, Max Planck Institute for Psycholinguistics, Nijmegen, The Netherlands; 6 Research School of Biology, Australian National University, Canberra, Australian Capital Territory Australia; 7 Earth Institute, University College Dublin, Dublin, Ireland; 8 Peter Medawar Building for Pathogen Research, Department of Zoology, University of Oxford, Oxford, UK; 9 Conway Institute of Biomolecular and Biomedical Science, University College Dublin, Dublin, Ireland; 10 Department of Biological Sciences, Texas Tech University, Lubbock, TX USA; 11 Department of Ecology and Evolution, Stony Brook University, Stony Brook, NY USA; 12 Consortium for Inter-Disciplinary Environmental Research, Stony Brook University, Stony Brook, NY USA; 13 Department of Sciences, John Jay College of Criminal Justice, New York, NY USA; 14 School of Biological Sciences, University of Bristol, Bristol, UK; 15 Department of Migration, Max Planck Institute of Animal Behavior, Radolfzell, Germany; 16 Department of Biology, University of Konstanz, Konstanz, Germany; 17 Smithsonian Tropical Research Institute, Panama City, Panama; 18 ISEM, University of Montpellier, Montpellier, France; 19 Zoological Institute and Museum, University of Greifswald, Greifswald, Germany; 20 Vertebrate Genomes Laboratory, The Rockefeller University, New York, NY USA; 21 Laboratory of Neurogenetics of Language, The Rockefeller University, New York, NY USA; 22 Howard Hughes Medical Institute, Chevy Chase, MD USA; 23 Donders Institute for Brain, Cognition and Behaviour, Nijmegen, The Netherlands; 24 Faculty of Computer Science, Technical University Dresden, Dresden, Germany

**Keywords:** Phylogenetics, Evolutionary biology, Genomics, Virology

## Abstract

Bats possess extraordinary adaptations, including flight, echolocation, extreme longevity and unique immunity. High-quality genomes are crucial for understanding the molecular basis and evolution of these traits. Here we incorporated long-read sequencing and state-of-the-art scaffolding protocols^1^ to generate, to our knowledge, the first reference-quality genomes of six bat species (*Rhinolophus ferrumequinum*, *Rousettus aegyptiacus*, *Phyllostomus discolor*, *Myotis myotis*, *Pipistrellus kuhlii* and *Molossus molossus*). We integrated gene projections from our ‘Tool to infer Orthologs from Genome Alignments’ (TOGA) software with de novo and homology gene predictions as well as short- and long-read transcriptomics to generate highly complete gene annotations. To resolve the phylogenetic position of bats within Laurasiatheria, we applied several phylogenetic methods to comprehensive sets of orthologous protein-coding and noncoding regions of the genome, and identified a basal origin for bats within Scrotifera. Our genome-wide screens revealed positive selection on hearing-related genes in the ancestral branch of bats, which is indicative of laryngeal echolocation being an ancestral trait in this clade. We found selection and loss of immunity-related genes (including pro-inflammatory NF-κB regulators) and expansions of anti-viral APOBEC3 genes, which highlights molecular mechanisms that may contribute to the exceptional immunity of bats. Genomic integrations of diverse viruses provide a genomic record of historical tolerance to viral infection in bats. Finally, we found and experimentally validated bat-specific variation in microRNAs, which may regulate bat-specific gene-expression programs. Our reference-quality bat genomes provide the resources required to uncover and validate the genomic basis of adaptations of bats, and stimulate new avenues of research that are directly relevant to human health and disease^1^.

## Abstract (web summary)

Reference-quality genomes for six bat species shed light on the phylogenetic position of Chiroptera, and provide insight into the genetic underpinnings of the unique adaptations of this clade.

## Main

With more than 1,400 species identified to date^2^, bats (Chiroptera) account for about 20% of all extant mammal species. Bats are found around the world and successfully occupy diverse ecological niches^1^. Their global success is attributed to an extraordinary suite of adaptations^1^ including powered flight, laryngeal echolocation, vocal learning, exceptional longevity and a unique immune system that probably enables bats to better tolerate viruses that are lethal to other mammals (such as severe acute respiratory syndrome-related coronavirus, Middle East respiratory syndrome-related coronavirus and Ebola virus)^3^. Bats therefore represent important model systems for the study of extended healthspan^4^, enhanced disease tolerance^3^, vocal communication^5^ and sensory perception^6^. To understand the evolution of bats and the molecular basis of these traits, we generated reference-quality genomes for six bat species as part of the Bat1K global genome consortium^1^ (http://bat1k.com) in coordination with the Vertebrate Genome Project (https://vertebrategenomesproject.org). These six bat genera span both major suborders Yinpterochiroptera (*R. ferrumequinum* and *R. aegyptiacus*) and Yangochiroptera (*P. discolor*, *M. myotis*, *P. kuhlii*, *M. molossus*)^7^ (Supplementary Table 1), represent extremes in bat longevity^8^, possess major adaptations in bat sensory perception^1^ and can better survive viral infections as compared with other mammals^3^.

## Genome sequencing and assembly

**Fig. 1:**
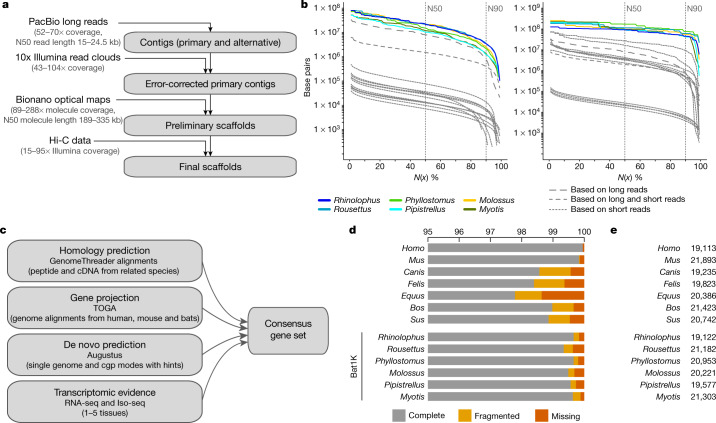
Assembly and annotation of the genomes of six bat species. **a**, Genome assembly strategy and data produced. **b**, Comparison of assembly contiguity. *N*(*x*) % graphs show contig (left) and scaffold (right) sizes (*y*-axis), in which *x* per cent of the assembly consists of contigs and scaffolds of at least that size. Coloured lines refer to species with Bat1K assemblies. Extended Data Figure 1 labels all previous bat assemblies (shown as grey lines here). **c**, Overview of our strategy to annotate coding genes combining various types of evidence. cgp, comparative gene prediction. **d**, Comparison of the completeness of gene annotations, as a percentage of 4,101 mammalian genes from BUSCO. **e**, Total number of annotated genes.

**Extended Data Fig. 1:**
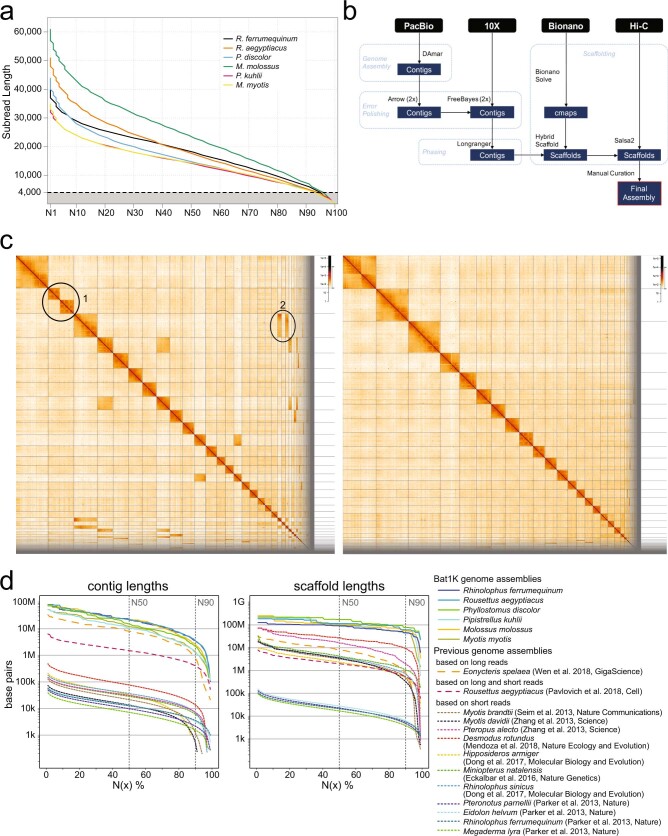
Genome assembly of six bats. **a**, Distribution of PacBio read lengths. The dashed line at 4 kb marks the minimum read length that was used in the assemblies. **b**, Detailed Bat1K assembly pipeline, listing the steps and methods used to assemble the genomes. **c**, Hi-C maps for *M. myotis* prior to (left) and post manual curation (right). Hi-C maps were created by mapping and filtering the Hi-C read pairs by using the tools bwa, pairsamtools, pairix and cooler following the Hi-C data processing pipeline on https://github.com/hms-dbmi/hic-data-analysis-bootcamp. Left, ellipse 1 shows that scaffold 2 contains a false join. It was split in the manual curation step. Ellipse 2 highlights two scaffolds, which were not joined in automated scaffolding steps but were manually integrated into scaffold 3. **d**, Detailed comparison of assembly contiguity of bat genomes. *N*(*x*)% graphs show the contig (left) and scaffold (right) sizes (*y*-axis), in which *x*% of the assembly consists of contigs and scaffolds of at least that size. Solid lines show assemblies generated by Bat1K (this study), dashed lines show previous assemblies of bat genomes (*Myotis brandtii*^16^, *Myotis davidii*^19^, *Pteropus alecto*^19^, *Desmodus rotundus*^18^, *Eonycteris spelaea*^17^, *Hipposideros armiger*^12^, *Rhinolophus sinicus*^12^, *Miniopterus natalensis*^13^, *Rousettus aegyptiacus*^15^, *Pteronotus parnellii*^14^, *Eidolon helvum*^14^, *Rhinolophus ferrumequinum*^14^ and *Megaderma lyra*^14^). *Eonycteris spelaea* was assembled using only PacBio long reads; the previous *Rousettus aegyptiacus* assembly is based on both long and short reads. All other previous assemblies were assembled using only short reads. Assembly gaps were defined as runs of ≥ 10 Ns.

**Extended Data Fig. 2:**
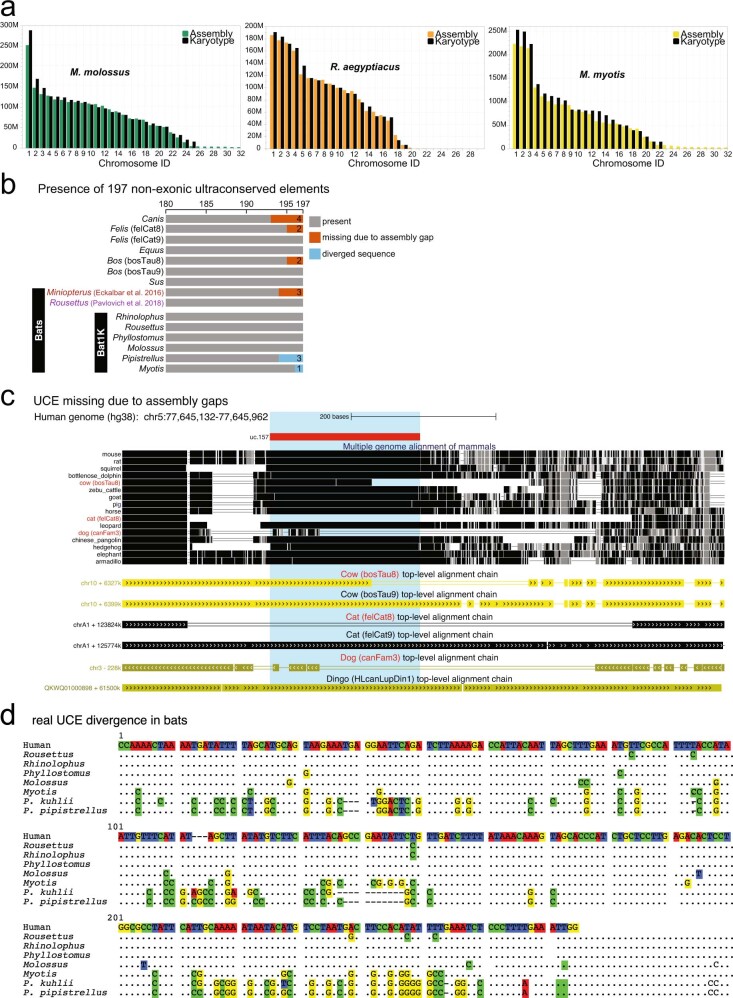
Chromosome lengths and comparison of assembly completeness in nonexonic genomic regions. **a**, Comparison of scaffold lengths and chromosome lengths that were estimated from published karyotype images of *M. molossus*, *M. myotis* and *R. aegyptiacus*. **b**, To assess completeness in nonexonic genomic regions, we determined how many of 197 nonexonic ultraconserved elements (UCEs)^20^ align at ≥ 85% identity to the human sequence. UCEs are highly conserved among mammals^88^ and are expected to be present in complete assemblies. Bar charts show the number of detected UCEs that align at these stringent parameters. As expected, the vast majority of UCEs were detected in all assemblies. UCEs not detected are separated into those that are missing owing to assembly incompleteness and those that exhibit real sequence divergence. In the bat genomes we report, no UCEs were missing owing to assembly incompleteness. Instead, one to three UCEs were not detected in our *Myotis* and *Pipistrellus* assemblies because the UCE sequences are more than 85% diverged (Supplementary Fig. 1). Human and mouse are not shown here because both genomes were used to define ultraconserved elements^20^. For cow and cat, we also compared new assemblies (bosTau9 and felCat9) that recently became available. **c**, Example of a UCE that is not fully present in the assemblies of cow (bosTau8), cat (felCat8) and dog (canFam3) because of assembly gaps. UCSC genome browser screenshot shows a multiple genome alignment of mammals of the locus around UCE.157 (highlighted) and pairwise chains of co-linear alignments (blocks represent local alignments, double lines represent unaligning sequence and single lines represent deletions). The top-level pairwise alignment chains between human (reference species) and cow, cat and dog show that UCE.157 only partially aligns (cow bosTau8 and dog canFam3) or does not align at all (cat felCat8). The unaligning region overlaps an assembly gap in all three cases, indicating that the UCE sequence is not present because of assembly incompleteness. Indeed, the UCE is entirely present in more-recent assemblies of cow (bosTau9) and cat (felCat9). Furthermore, the alignment chains of the dingo—a close relative of the dog—show that the dingo assembly also contains the entire UCE.157. **d**, Example of a UCE that shows real sequence divergence in *Pipistrellus* bats. Dots in the alignment represent nucleotides that are identical to the human sequence shown at the top. Compared to other bats, *Pipistrellus kuhlii* shows an increased number of mutations in this UCE sequence; however, *M. myotis* also shows an increased number of mutations. Because most mutations are shared between *P. kuhlii* and *Pipistrellus pipistrellus*, base errors in the assembly are highly unlikely to account for the increased sequence divergence.

To obtain genome assemblies of high contiguity and completeness, we developed pipelines that incorporate state-of-the-art sequencing technologies and assembly algorithms (Supplementary Notes 1, 2). In brief, we generated PacBio continuous long reads, 10x Genomics Illumina read clouds, Bionano optical maps and chromosome conformation capture (Hi-C) Illumina read pairs for each bat species (Fig. 1a). We assembled the PacBio reads into contigs using a customized assembler we termed DAmar, a hybrid of the earlier Marvel^9^, Dazzler and Daccord^10,11^ systems. Next, we used 10x Illumina read-cloud data to correct base errors and phase haplotypes, arbitrarily picking one haplotype in a phased block. Finally, we used Bionano optical maps and then Hi-C data to produce long-range scaffolds (Extended Data Fig. 1a, b, Supplementary Note 2). For all six bat species, this resulted in assemblies with high contiguity: 96–99% of each assembly is in chromosome-level scaffolds (N50 values of 92–171.1 Mb) (Fig. 1b, Extended Data Figs. 1c, d, 2a). When compared with previously published bat genomes^12–19^, our assemblies have higher contig N50 values—ranging from 10.6 to 22.2 Mb—and therefore, these are two orders of magnitude more contiguous than bat genomes assembled from short-read data alone (Fig. 1b, Extended Data Fig. 1d, Supplementary Tables 2, 3, Supplementary Note 2). Similarly, our genomes are estimated to have near-100% gene completeness (see ‘Gene annotation’) (Fig. 1c, d, Supplementary Table 4, Supplementary Note 3.1). Furthermore, analysis of 197 nonexonic ultraconserved elements^20^ indicates a high completeness of nonexonic genomic regions. This analysis also revealed three cases of marked sequence divergence of ultraconserved elements in vespertilionid bats—something rarely observed in these elements, which are highly constrained amongst placental mammals (Extended Data Fig. 2b–d, Supplementary Figs. 1–3, Supplementary Table 5, Supplementary Note 3.2). In summary, these genomes are comparable to the best reference-quality genomes that have so far been generated for any eukaryote with a gigabase-sized genome^21^.

## Gene annotation

**Extended Data Fig. 3:**
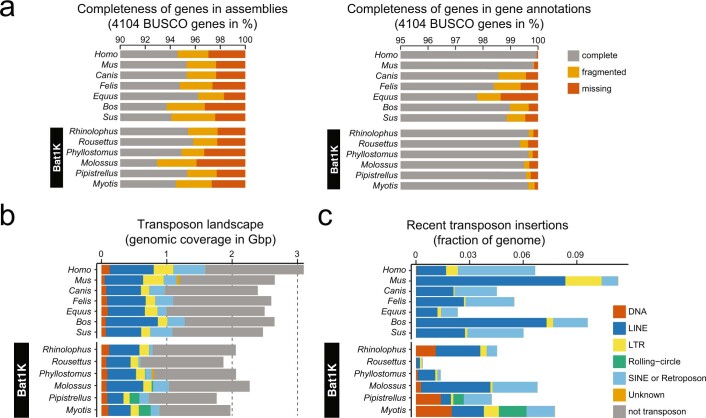
Comparison of assembly completeness in coding genomic regions and transposon content. **a**, BUSCO applied to genomic sequences markedly underestimates gene completeness of assemblies. Bar charts show the percent of 4,104 highly conserved mammalian BUSCO genes that are completely present, fragmented or missing in the assembly. Left, applying BUSCO to genome assemblies. Right, applying BUSCO to the gene annotations (protein sequences of annotated genes; this panel is reproduced from Fig. 1d to enable a direct comparison). The direct comparison shows that BUSCO applied to the whole genome detects markedly fewer genes than BUSCO applied to the gene annotation. Because every annotated gene is by definition present in the assembly, this shows that BUSCO applied to the whole genome underestimates gene completeness—probably because it is substantially more difficult to detect complete genes in assemblies. **b**, Comparison of genomic transposon composition between six bats and other representative boreoeutherian mammals (Laurasiatheria + Euarchontoglires), selected for the highest genome contiguity. We used a previously described workflow and manual curation to annotate TEs^82^. Bar charts compare genome sizes and the proportion that consist of major transposon classes. TE content generally relates with genome size. Our assemblies also revealed noticeable genome size differences within bats, with assembly sizes ranging from 1.78 Gb for *Pipistrellus* to 2.32 Gb for *Molossus*. **c**, Fraction of the genome that consists of recent transposon insertions. We compared TE copies to their consensus sequence to obtain a relative age from each TE family. This revealed an extremely variable repertoire of TE families with evidence of recent accumulation (defined as TE insertions that diverged less than 6.6% from their consensus sequence). For example, while only about 0.38% of the 1.89-Gb *Rousettus* genome exhibits recent TE accumulations, about 4.2% of the similarly sized 1.78-Gb *Pipistrellus* genome is derived from recent TE insertions. The types of TE that underwent recent expansions also differ substantially in bats compared to other mammals, particularly with regards to the evidence of recent accumulation by rolling-circle and DNA transposons in the vespertilionid bats. These two TE classes have been largely dormant in most mammals for the past approximately 40 million years and recent insertions are essentially absent from other boreoeutherian genomes^31^. These results add to previous findings revealing a substantial diversity in TE content within bats, with some species exhibiting recent and ongoing accumulation from TE classes that are extinct in most other mammals while other species show negligible evidence of TE activity^32^.

To comprehensively annotate protein-coding genes, we integrated different types of genetic evidence—including short-read (RNA sequencing (RNA-seq)) and long-read (isoform sequencing (Iso-Seq)) transcriptomic data from our bat species, gene projections by TOGA, aligned protein and cDNA sequences of related mammals, and de novo gene predictions (Fig. 1c). For the six bat species, we annotated between 19,122 and 21,303 protein-coding genes (Fig. 1e). Using the 4,104 mammalian genes in the ‘Benchmarking Universal Single-Copy Orthologs’ (BUSCO)^22^ set, we achieved 99.3–99.7% completeness (Fig. 1d); this shows that our assemblies and annotations are highly complete in protein-coding sequences (Extended Data Fig. 3a). Importantly, the completeness of our gene annotations is higher than available annotations of dog, cat, horse, cow and pig, and is only surpassed by those of human and mouse, which have received extensive manual curation (Fig. 1d, Supplementary Table 4). Thus, reference-quality genome assemblies combined with multiple types of gene evidence can generate high-quality and near-complete gene annotations of bats. This strategy can be extended to other species to improve genome assembly and annotation. All individual evidence and final gene sets can be visualized in the Bat1K genome browser (https://genome-public.pks.mpg.de) and downloaded from https://bds.mpi-cbg.de/hillerlab/Bat1KPilotProject/.

## Genome sizes and transposable elements

At about 2 Gb in size, bat genomes are generally smaller than genomes of other placental mammals^1^ (which are typically 2.5–3.5 Gb). By annotating transposable elements in our genomes (Supplementary Note 3.3), we found that smaller genome size is related to lower transposable element content (Extended Data Fig. 3b). Recently inserted transposable elements in the bat genomes are extremely variable in terms of their type and number, as compared to other mammals (Extended Data Fig. 3c). In vespertilionid bats, we detected recent activity of rolling-circle and DNA transposon classes that have been largely dormant in other mammals for over 40 million years^23^. In summary, bats exhibit substantial diversity in transposable element content, and diverse transposable element classes show evidence of recent activity.

## The phylogenetic origin of Chiroptera

**Fig. 2:**
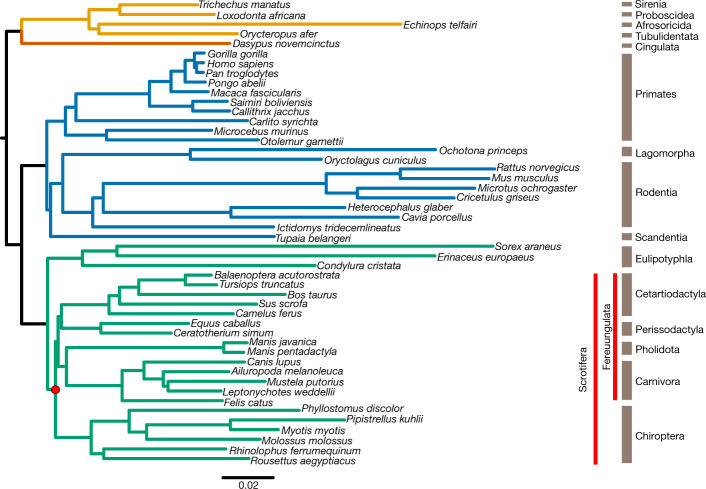
Phylogenetic analysis of Laurasiatheria. We inferred a mammalian phylogram using a supermatrix of 12,931 concatenated genes and the maximum likelihood method of tree reconstruction (topology 1 in Supplementary Fig. 4). All nodes received 100% bootstrap support. The divergence of Chiroptera and Fereuungulata is highlighted with a red dot. The mammalian superordinal groups are denoted as follows: green, Laurasiatheria; blue, Euarchontoglires; orange, Xenarthra; yellow, Afrotheria.

Identifying the evolutionary origin of bats within the mammalian clade Laurasiatheria is a key prerequisite for any comparative analyses. However, the phylogeny of Laurasiatheria and—in particular—the origin of bats is a long-standing and unresolved phylogenetic question^24^, as multiple phylogenetic and systematic studies support alternative topologies^25^. These incongruent results have been attributed to the challenge of identifying the two (presumably short) internal branches that link the four key clades that diverged in the Late Cretaceous period^26^—that is, Chiroptera, Cetartiodactyla, Perissodactyla and (Carnivora + Pholidota) (Fig. 2, Supplementary Table 1).

**Extended Data Fig. 4:**
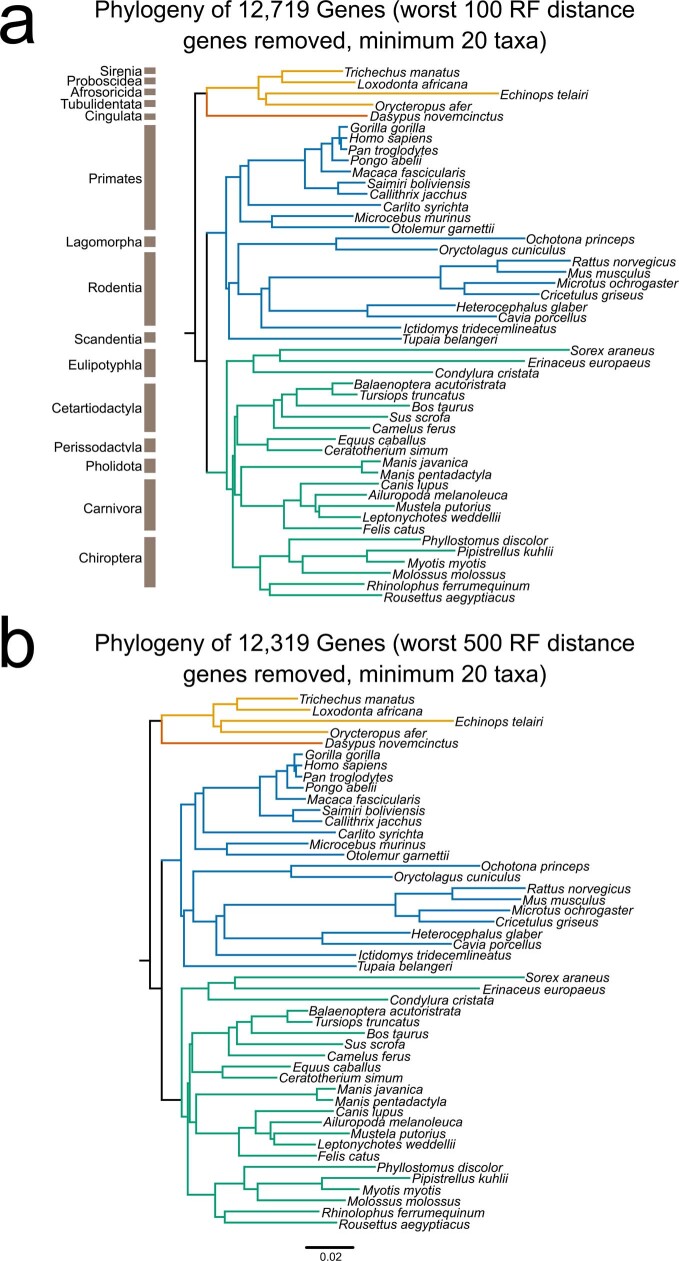
Exploring the effect of gene alignment quality using Robinson–Foulds distances. **a**, We set a minimum taxa number to 20, excluding all genes that did not meet this criterion. Additionally, we calculated the Robinson–Foulds (RF) distance for each individual gene tree relative to topology 1 (Supplementary Fig. 4), and excluded the 100 most distant gene alignments from the supermatrix. This was done to explore the effects of low taxa number and homology error (Supplementary Note 4.2.2) on species tree topology. The resulting topology showed no difference in branching pattern compared to the full supermatrix analyses (Fig. 2). **b**, We also excluded the 500 most divergent genes, to determine the effect that putative homology errors might have on the overall topology, and observed no difference.

**Extended Data Fig. 5:**
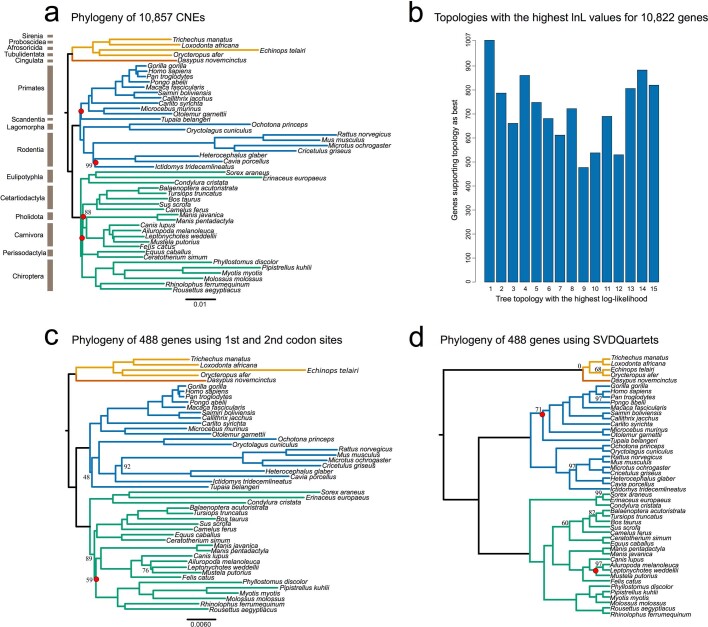
Phylogenetic analyses of Laurasiatheria. **a**, A total of 10,857 conserved noncoding elements (CNEs) were used to determine a mammalian phylogeny using noncoding regions (topology 2 in Supplementary Fig. 4). Bootstrap support values less than 100 are displayed, with internal nodes that differ to the protein-coding supermatrix highlighted in red. The position of Chiroptera as basal to Fereuungulata, as in Fig. 2, is maintained. **b**, All gene alignments were fit to the 15 laurasiatherian topologies (Supplementary Fig. 4) we explored, to determine which tree had the highest likelihood score for each gene. The number of genes supporting each topology is displayed. **c**, A supermatrix consisting of 1st and 2nd codon sites from 448 genes that are evolving under homogenous conditions—thus considered optimal ‘fit’ for phylogenetic analysis—was used to infer a phylogeny using maximum likelihood (topology 13 in Supplementary Fig. 4). Bootstrap support values less than 100 are displayed, with internal nodes that differ to the protein-coding supermatrix phylogeny highlighted in red. Unlike Fig. 2, Chiroptera is now sister to (Carnivora + Pholidota); however, this split has low bootstrap support (58%). **d**, Using the 488 genes considered fit for phylogenetic analyses, the position of bats within Laurasiatheria under a model of coalescence using SVDquartets. The resulting phylogeny is displayed. The tree is rooted on Atlantogenata, with support values from bootstrap pseudoreplicates. Only nodes with support less than 100 have their values displayed. The position of Chiroptera as basal to Fereuungulata, as in Fig. 2, is maintained.

We revisited this question, leveraging the high completeness of our gene annotations. We extracted a comprehensive dataset of 12,931 orthologous protein-coding genes using TOGA (21,468,943 aligned nucleotides in length and 7,911,881 parsimony-informative sites) and 10,857 orthologous conserved noncoding elements (5,234,049 aligned nucleotides and 1,234,026 parsimony-informative sites) from 48 mammalian genomes (Supplementary Note 4.1). We concatenated each of these datasets, identified the optimal model of sequence evolution with ModelFinder^27^ (Supplementary Table 6), inferred the species tree under maximum likelihood using the model-partitioned dataset with IQ-TREE^28^, rooted using Atlantogenata^29^, and obtained 1,000 bootstrap replicates to estimate branch support (Supplementary Note 4.2). For each protein-coding gene, we also compared the optimal gene tree inferred under maximum likelihood to the species tree, using the Robinson–Foulds distance to identify gene alignments with possibly incorrect homology statements^30^ (Supplementary Note 4.2.2). Our analysis of concatenated protein-coding genes identified the origin of bats within Laurasiatheria with 100% bootstrap support across the entire tree (Fig. 2). Omitting the top-scoring 100 and 500 genes (based on Robinson–Foulds distance) from the phylogenetic data produced the same tree topology, which suggests a small effect of homology error on the inferred phylogeny (Extended Data Fig. 4a, b). The tree inferred from the conserved noncoding element data identified the same phylogenetic position of bats, and differed from that shown in Fig. 2 only in the position of Perissodactyla (most closely related to Carnivora + Pholidota rather than to Cetartiodactyla) (Extended Data Fig. 5a). Therefore, both coding and noncoding regions of the genome support an early split between Eulipotyphla and the rest of the laurasiatherians (that is, Scrotifera); within Scrotifera, Chiroptera is the sister clade to Fereuungulata (Cetartiodactyla + Perissodactyla + Carnivora + Pholidota). This tree challenges the Pegasoferae hypothesis^31^, which groups bats with Perissodactyla, Carnivora and Pholidota, but agrees with a previous study of concatenated phylogenomic data^32^. Evolutionary studies of 102 retrotransposons, which considered incomplete lineage sorting, also supported a sister-group relationship between Chiroptera and Fereuungulata, but differ from the present study in supporting a sister-group relationship between Carnivora and Cetartiodactyla^25,26^.

Next, we considered potential phylogenetic problems with our data and methods. First, as the number of homologous sites increases in phylogenomic datasets, so too does bootstrap support^33^—sometimes even for an incorrect tree^34^. Therefore, we estimated the maximum likelihood support of each protein-coding gene (*n* = 12,931) for the 15 bifurcating trees that represent all possible topologies of the 4 key clades (Supplementary Fig. 4), with Eulipotyphla as the outgroup and the clade subtrees as in Fig. 2. We found that the best-supported tree is identical to the tree estimated from our concatenated protein-coding gene set (Fig. 2; tree 1 with 1,007/10,822 genes, described in Extended Data Fig. 5b and Supplementary Note 4.2.1) and shows the sister-group relationship between Chiroptera and Fereuungulata, which is also supported by the conserved noncoding elements (Extended Data Fig. 5a). Second, model misspecification (owing to a poor fit between phylogenetic data and the model of sequence evolution used) or loss of the historical signal^35^ can cause biases in phylogenetic estimates^36^. To assess whether these factors may have confounded our phylogenetic estimate (Fig. 2), we examined the 12,931 alignments of protein-coding genes for evidence of violating the assumption of evolution under homogeneous conditions (assumed by the phylogenetic methods used here) and for evidence that the historical signal has decayed almost completely (owing to multiple substitutions at the same sites; Supplementary Note 4.2). A total of 488 gene alignments, comprising 1st and 2nd codon sites from all 48 taxa (241,098 sites and 37,588 parsimony-informative sites), were considered optimal for phylogenetic analysis and were concatenated into a data matrix (Supplementary Table 7). Maximum likelihood trees were generated but resulted in an ambiguous phylogenetic estimate (Extended Data Fig. 5c, topology 13 in Supplementary Fig. 4, Supplementary Note 4.2). Therefore, we analysed these 488 genes individually using SVDquartets^37^, a single-site coalescence-based method that provides an alternative to phylogenetic analysis of a concatenation^26^. The inferred optimal tree again supported Chiroptera as sister group to Fereuungulata (Extended Data Fig. 5d, topology 1 in Supplementary Fig. 4), which is the most-supported position from all of our analyses and data partitions. Taken together, multiple lines of evidence from across the genome provide the highest support for Chiroptera as basal within Scrotifera (Fig. 2).

## Screens for gene selection, losses and gains

**Extended Data Fig. 6:**
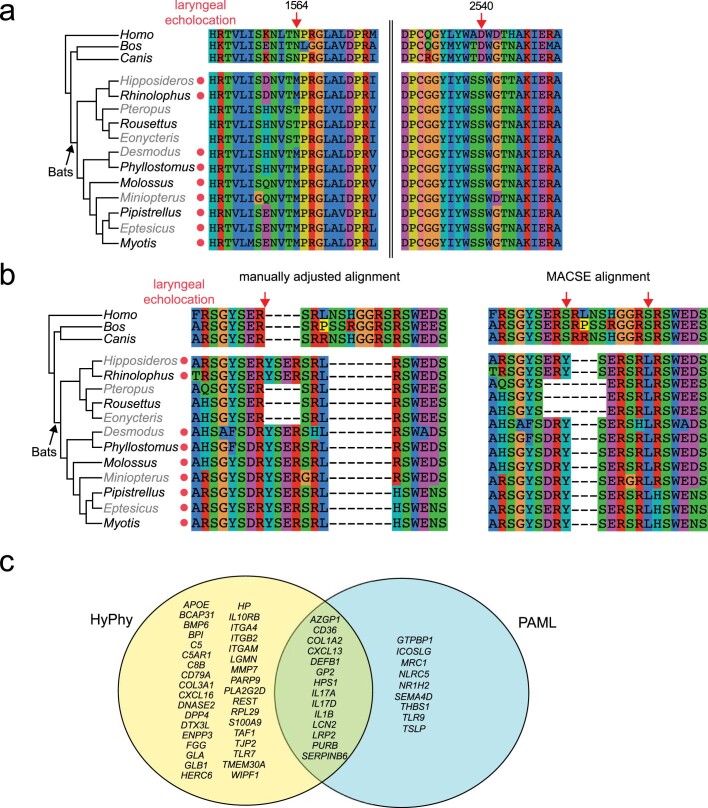
Screens for positive selection in genes in bats. **a**, Sites under positive selection in bats in the *LRP2* gene. Multiple sequence alignments of local regions surrounding two bat-specific mutations, which were found to be under positive selection (BEB > 0.95) using codeml (PAML). Site 1564 shows bat-specific changes at a conserved residue. The paraphyletic echolocating bats (indicated by a red dot) all share a methionine at this site, whereas pteropodid bats—which do not use laryngeal echolocation—have a threonine at this site. Site 2540 shows a bat-specific change, shared by all bats. The presence and patterns of mutations found in our six bats were confirmed in six previously published bat genomes, to increase taxonomic representation. Human (*Homo*), cow (*Bos*) and dog (*Canis*) are also shown. **b**, Echolocator-specific changes in the *TJP2* gene. We initially identified positive selection in the bat ancestor in the hearing-related gene *TJP2* (tight junction protein 2), which is expressed in cochlear hair cells and associated with hearing loss^43^. The right side shows the multiple sequence alignment produced by MACSE of local regions surrounding bat-specific mutations (red arrows), which were found to be under positive selection (BEB > 0.95) using codeml (PAML). The paraphyletic echolocating bats are indicated by a red dot. However, as shown on the left, manual inspection revealed a putative alignment ambiguity and manual adjustment produced an alignment with two bat-specific indels. This manually corrected alignment had a reduced significance for positive selection (aBSREL raw *P* = 0.009, not significant after multiple test correction considering 12,931 genes). The corrected alignment revealed a four-amino-acid microduplication found only in echolocating bats (*n* = 9) and not in pteropodid bats that lack laryngeal echolocation. This may be explained by incomplete lineage sorting or convergence. Insertions and deletions may also affect protein function, but are not considered by tests for positive selection; however, a phylogenetic interpretation of these events may uncover functional adaptations. **c**, Ageing and immune candidate genes showing evidence of significant positive selection using aBSREL (HyPhy, yellow) and codeml (PAML, blue). Genes identified by both methods are displayed at the intersection.

Using our best-supported species phylogeny (Fig. 2), we explored the genomic basis of exceptional traits shared by bats. We performed three unbiased genome-wide screens for gene changes that occurred in the six bat species. First, we screened the 12,931 protein-coding genes classified as 1:1 orthologues for signatures of positive selection on the ancestral bat branch (stem Chiroptera), under the aBSREL^38^ model using HyPhy^39^ (false discovery rate < 0.05) (Supplementary Note 4.3). We further required that the branch-site test implemented in codeml^40^ (part of the PAML package) independently verified positive selection, and manually excluded alignment ambiguities. This strict screen identified nine genes with diverse functions that have robust evidence of positive selection in the bat ancestor (Supplementary Table 8). This included the genes *LRP2* and *SERPINB6*, which—among other functions—are involved in hearing. Both genes are expressed in the cochlea and, in humans, are associated with disorders that involve deafness^41,42^ (Supplementary Note 4.3). *LRP2* has an amino acid substitution that is specific to bats with laryngeal echolocation, as pteropodid bats—which do not have laryngeal echolocation—exhibit a different, derived amino acid (Extended Data Fig. 6a). In a third hearing-related gene *TJP2*^43^, our analysis identified a putative microduplication that is also found only in echolocating bats (Extended Data Fig. 6b). These echolocator-specific mutations were further confirmed using publicly available bat genomes (*n* = 6) and all three genes were found not to be under positive selection in the non-bat-ancestral lineages (that is, Cetartiodactyla and Carnivora) using our strict selection protocols (Supplementary Note 4.3.3). If these mutations and the ancestral signatures of selection in these genes are indeed related to echolocation, this would provide molecular evidence that laryngeal echolocation evolved once in the bat ancestor with a subsequent loss in pteropodids rather than as multiple independent acquisitions within the echolocating bats, informing a long-standing debate in bat biology on the origin of echolocation^44^.

In addition to hearing-related genes, our genome-wide screen also revealed bat-specific selection on several immunity-related genes: the B-cell-specific chemokine *CXCL13*^45^, the asthma-associated *NPSR1*^46^ and *INAVA*, a gene that is involved in intestinal barrier integrity and enhancing NF-κB signalling in macrophages^47^. Changes in these genes may have contributed to the unique tolerance of pathogens among bats^3^. By specifically testing 2,453 candidate genes with immune- and age-related Gene Ontology terms (Supplementary Note 4.3), and strictly requiring significance by both aBSREL and codeml with multiple test correction (false discovery rate < 0.05), we found 10 additional genes with robust evidence of positive selection in the ancestral bat lineage (Extended Data Fig. 6c, Supplementary Table 9, Supplementary Note 4.3.2). These additional genes include *IL17D*^48^ and *IL1B*^49^, which are involved in immune system regulation and NF-κB activation, and *LCN2*^50^ and *GP2*^51^, which are involved in responses to pathogens. We further used I-TASSER^52^ to model the three-dimensional (3D) structure of all of the proteins encoded by the genes under positive selection, and to estimate the effect of the bat-specific residues on protein structure and stability. Our results show that bat-specific substitutions with significant support for positive selection are predicted to have stabilizing or destabilizing effects (for example, *AZGP1* and *INAVA*), which may affect protein function (Supplementary Note 4.4). Some bat-specific substitutions also occur in or near regions that may be directly involved in ligand-binding (for example, *DEFB1*, *LCN2*, *SERPINB6* and *KBTBD11*). Overall, combining genome-wide and candidate screens revealed several candidate genes, which suggests that ancestral bats evolved immunomodulatory mechanisms that enabled a higher tolerance to pathogens than is typical amongst mammals. Consistent with this, repeating the stringent genome-wide screen to detect selection on comparable, ordinal branches leading to the ancestors of Carnivora and Cetartiodactyla revealed fewer immune-related genes (three and four genes for Carnivora and Cetartiodactyla, respectively) (Supplementary Table 10, Supplementary Note 4.3.3).

**Fig. 3:**
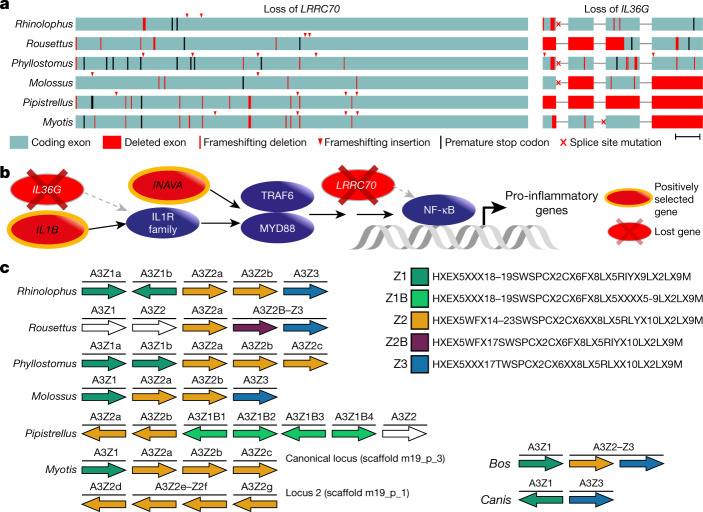
Genome-wide screens highlight changes in genes that are potentially involved in exceptional immunity in bats. **a**, Inactivation of the immune-stimulating genes *LRRC70* and *IL36G*. Boxes represent coding exons proportional to their size, overlaid with gene-inactivating mutations present in six bat species. Scale bar, 100 bp. **b**, Diagram showing the canonical NF-κB signalling pathway (purple) and interacting proteins that have experienced positive selection or have been lost in bats. **c**, Expansion of the *APOBEC3* gene locus. Each arrow represents a cytidine deaminase domain, coloured by domain subtypes as defined by the given motifs (right), with likely pseudogenes shown in white. Genes containing multiple deaminase domains are indicated with a single bar over more than one domain. A transposition event in *Myotis* created two *APOBEC3* loci on different chromosomes. Cow and dog are two Laurasiatheria outgroups; cow also represents the likely mammalian ancestral state. Within the given motifs, X denotes any amino acid.

**Extended Data Fig. 7:**
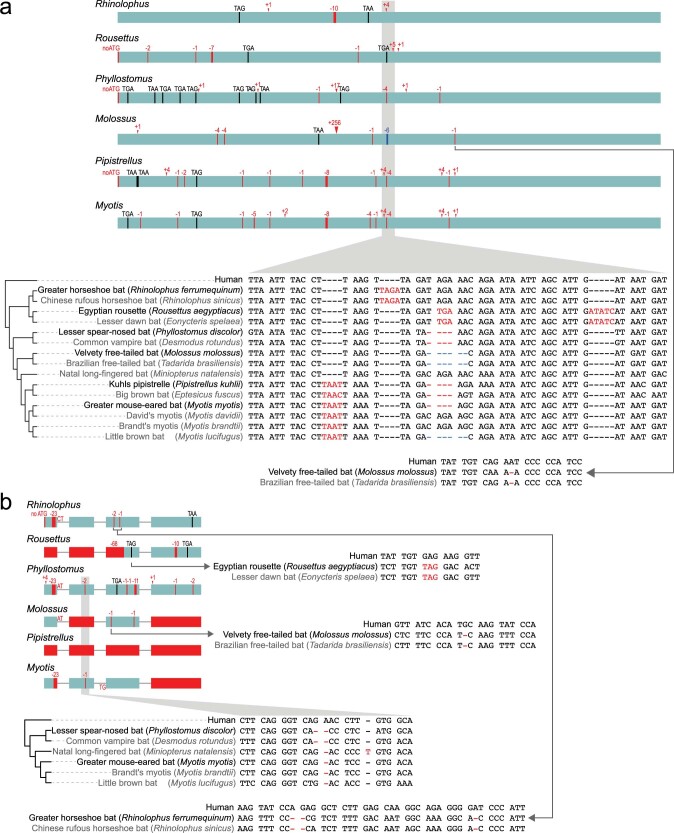
Inactivating mutations in *LRRC70* and *IL36G* genes in bats. **a**, **b**, *LRRC70* (**a**) is expressed in a broad range of tissues and potentiates cellular responses to multiple cytokines^54^ and is well-conserved among Laurasiatheria. Importantly, *LRRC70* strongly amplifies bacterial-lipopolysaccharide-mediated NF-κB activation^54^. Our finding of *LRRC70* loss in bats makes this poorly characterized gene an interesting target for future mechanistic studies. *IL36G* (**b**) encodes a proinflammatory interleukin belonging to the interleukin-1 family. Increased expression of *IL36G* was detected in patients with psoriasis or inflammatory bowel disease, and *IL36G* is probably involved in the pathophysiology of these diseases by inducing the canonical NF-κB pathway and other proinflammatory cytokines^55–57^. Coding exons are represented as boxes (*LRRC70* has only a single coding exon), superimposed with all detected inactivating mutations. Vertical red lines show frameshifting deletions; arrowheads indicate frameshifting insertions. Red boxes indicate complete or partial exon deletions. The size of deletions or insertions is given on top of the mutation. Premature stop codons are indicated by black vertical lines and the corresponding triplet. Mutated ATG start codons are indicated as ‘noATG’. Splice site mutations are shown by red letters at the end of an exon (donor mutation) or the beginning of an exon (acceptor mutation). One representative mutation for each bat is shown in detail in the alignment between human and bats (red font indicates the inactivating mutation). Genome assemblies produced in this study are in black; publicly available assemblies of sister species are in grey font. For both genes, the presence of the exact same mutation in independently sequenced and assembled genomes of sister species excludes the possibility that the representative mutations are erroneous. This analysis also reveals that both genes were in fact lost multiple times within Chiroptera, suggesting these genes came under relaxed selection in bats followed by with subsequent gene losses. In **a**, the position of the −4-bp frameshifting deletion in *LRRC70* in *Pipistrellus* and *Myotis* is ambiguous and can be shifted by up to 3 bp to the right without affecting alignment identity.

In our second genome-wide screen, we used a previously developed approach^53^ to systematically screen for gene losses (Supplementary Note 4.5). This revealed 10 genes that are inactivated in our 6 bat species but that are present in the majority of non-bat members of Laurasiatheria (Supplementary Table 11). Two of these lost genes have immune-stimulating functions (Fig. 3a). *LRRC70* is a broadly expressed gene that potentiates cellular responses to multiple cytokines and amplifies NF-κB activation mediated by bacterial lipopolysaccharides^54^. *IL36G* is overexpressed in patients with psoriasis or inflammatory bowel disease, and encodes a pro-inflammatory interleukin that induces the canonical NF-κB pathway and other pro-inflammatory cytokines^55–57^. We confirmed the loss of these genes in additional, publicly available bat genomes (*n* = 9) (Extended Data Fig. 7). Together, genome-wide screens for gene loss and positive selection revealed several genes involved in NF-κB signalling (Fig. 3b, Supplementary Note 4.3), which suggests that altered NF-κB signalling may contribute to immune-related adaptations in bats.

Third, we investigated changes in the sizes of gene families, which revealed 35 gene families that exhibit significant expansions or contractions in the bat ancestor (Supplementary Table 12). Among these, we inferred an expansion of the APOBEC gene family caused by expansion at the *APOBEC3* locus (Fig. 3c), which is known to exhibit a complex history of duplication and loss in the flying foxes (*Pteropus* genus)^58^ as well as in other mammals^59^. Our detailed analysis indicates a small expansion of *APOBEC3* in the ancestral bat lineage, followed by multiple, lineage-specific expansions that involve up to 14 duplication events (Supplementary Fig. 5, Supplementary Note 4.6), including the generation of a second *APOBEC3* locus in *Myotis*. *APOBEC3*-type genes encode DNA- and RNA-editing enzymes that can be induced by interferon signalling and are implicated in restricting viral infection and transposon activity^60,61^. Expansion of APOBEC3 genes in multiple bat lineages may contribute to viral tolerance in these lineages.

## Integrated viruses in bat genomes

**Extended Data Fig. 8:**
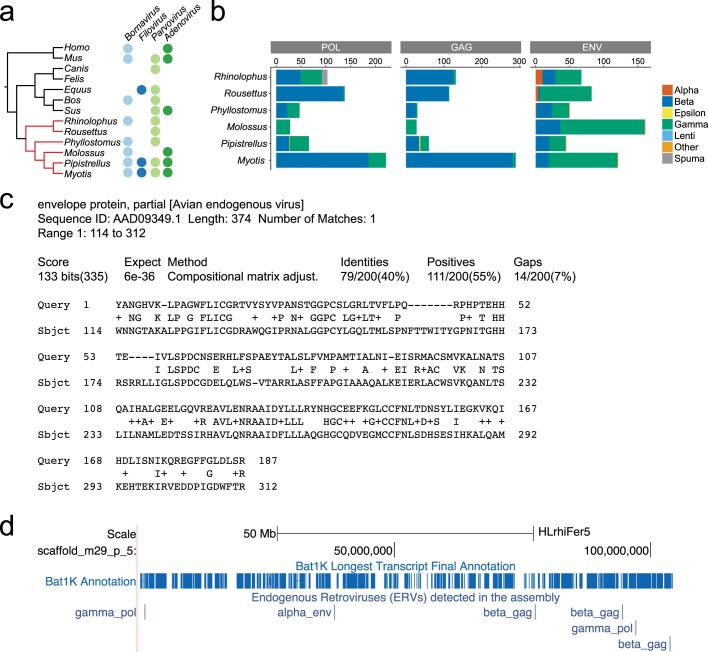
Viral sequences integrated in bat genomes. **a**, Viral families identified in more than one genus mapped to phylogenetic tree of six bat species and seven additional mammals. Using reciprocal BLAST searches and a custom comprehensive library of viral protein sequences, we screened our six bat genomes and seven mammalian outgroups for the presence of non-retroviral EVEs. Endogenous sequences identified as *Adenoviridae*, *Parvoviridae*, *Filoviridae* and *Bornaviridae* were represented across several mammalian genera. **b**, Bar plots show numbers of viral proteins of all seven *Retroviridae* genera detected in the genomes of our six bats. Beta-like integrations are most common for pol and gag proteins and gamma-like integrations are most common for env proteins. Overall, the highest number of integrations was observed in *Myotis* (*n* = 630), followed by *Rousettus* (*n* = 334) with *Phyllostomus* containing the lowest. **c**, Alignment exemplifies that an ERV found in *Rhinolophus* (scaffold_m29_p_13:24821733-24822323) best matches the env protein of an avian endogenous virus, which belongs to the alpharetrovirus group. **d**, UCSC genome browser screenshot (https://genome-public.pks.mpg.de/) of a 104 Mb scaffold (scaffold_m29_p_5) of the *Rhinolophus* assembly shows detected ERVs as an annotation track.

There is mounting evidence that suggests that bats can better tolerate and survive viral infections than most mammals, owing to adaptations in their immune response^3^. This is further supported by our findings of selection and loss of immune-related genes and expansions of the viral-restricting *APOBEC3* genes. As viral infections can leave traces in host genomes in the form of endogenous viral elements (EVEs)^62^, we screened our bat genomes to ascertain whether they contain a higher number and diversity of EVEs compared with other mammals (Supplementary Note 3.4). First, we focused on non-retroviral EVEs that generally are less abundant in animal genomes compared to endogenous retroviruses (ERVs)^62^. We identified three predominant non-retroviral families of EVEs—the *Parvoviridae*, *Adenoviridae* and *Bornaviridae*—in individual bat species and in other mammalian outgroups (Extended Data Fig. 8a). We also detected a partial filovirus EVE in Vespertilionidae (*Pipistrellus* and *Myotis*), which is consistent with a previous report that vespertilionid bats have—in the past—been exposed to and can survive filoviral infections^63^.

Second, we focused on retroviral protein-coding genes from all ERV classes. Consistent with other mammals, the highest number of integrations came from beta- and gamma-like retroviruses^64,65^ (Extended Data Fig. 8b, Supplementary Fig. 6). Notably, in the genomes of several bat species (*Phyllostomus, Rhinolophus*, and *Rousettus*), we found DNA that encodes viral envelope (Env) proteins that are more similar to those of the alpharetroviruses than to other retroviral genera (Extended Data Fig. 8b, c). Until now, alpharetroviruses have been considered as exclusively endogenous avian viruses^66^; consequently, our discovery of alpharetroviral-like elements in the genomes of several bat species suggests that bats have been infected by these viruses (Extended Data Fig. 8c). Phylogenetic analysis suggests that most viral integrations are relatively recent integration events (Supplementary Fig. 7). This analysis also revealed short *gag*-like fragments with similarity to lentiviruses in *Pipistrellus* (a retrovirus genus rarely observed in endogenized form)^67^, although it is not clear whether these resulted from ancient lentiviral integrations; two families of foamy retroviruses belonging to the spumaretroviruses in *Rhinolophus* (confirming the presence of endogenous spumaretroviruses in this species); and *pol*-like sequences clustering with deltaretroviruses in *Molossus*. Overall, these results show that bat genomes contain a diversity of ERVs, which provides evidence of past viral infections. The integrated ERVs are available as an annotation track in the Bat1K genome browser (https://genome-public.pks.mpg.de) (Extended Data Fig. 8d).

## Changes in noncoding RNAs

**Fig. 4:**
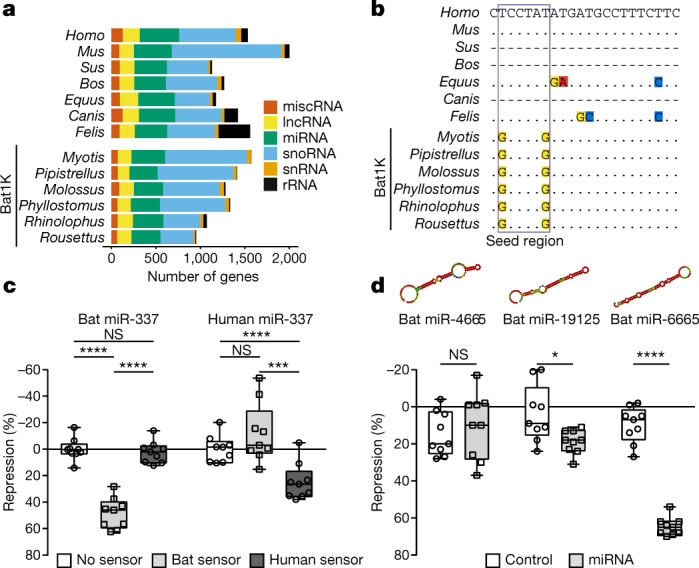
Evolution of noncoding RNAs in bats. **a**, Landscape of noncoding RNA genes. Number of noncoding RNA genes annotated in six bat and seven reference mammalian genomes. lncRNA, long noncoding RNA; miscRNA, miscellaneous RNA; rRNA, ribosomal RNA; snoRNA, small nucleolar RNA; snRNA, small nuclear RNA. **b**, Multispecies alignment of mature miR-337-3p. Dots represent bases identical to human; dashes represent species in which a functional miR-337-3p could not be identified. Extended Data Figure 9b gives alignment of mature miR-337-3p sequences across 40 mammals. **c**, Specificity differences of human and bat miR-337-3p activity is shown using species-specific sensors in luciferase reporter assays (*n* = 9 biologically independent samples in 3 independent experiments). Significance calculated using two-way analysis of variance test, followed by post hoc Tukey calculation. ****P < 0.0001, ***P = 0.0008; for bat-miR-337, not significant (NS) *P* = 0.9269; for human-miR-337, NS *P* = 0.1485. **d**, Validation of novel miRNA activity in ancestral bats. Predicted secondary structures are shown for each miRNA. Luciferase assays compare a negative control (unrelated miRNA not predicted to bind the sensor) and the cognate miRNA (*n* = 9 biologically independent samples in 3 independent experiments). Significance for each control–miRNA pair calculated using pairwise *t*-tests, type 2. *P = 0.0157; ****P < 0.0001; NS, *P* = 0.5475. Box plots extend from 25th to 75th percentiles, central line represents the median value, and whiskers extend to the smallest and largest values.

**Extended Data Fig. 9:**
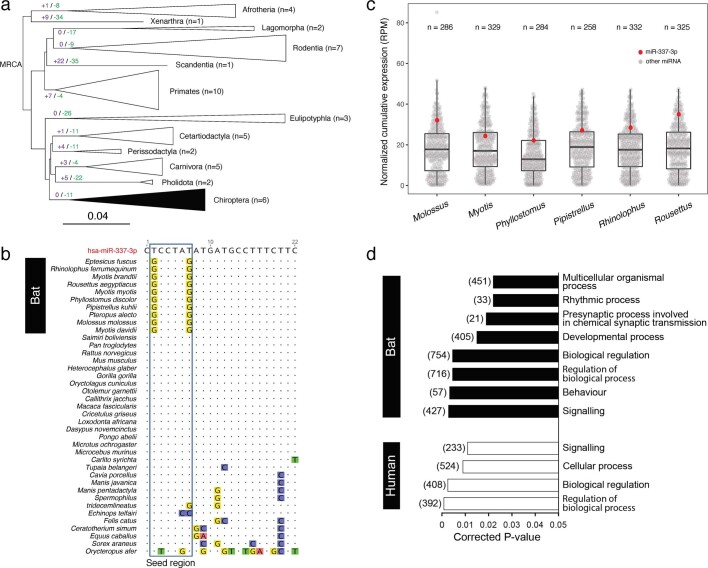
Evolution of miRNAs in bats. **a**, miRNA family expansion and contraction analyses in 48 mammalian genomes. The number of miRNA families expanded and contracted are annotated at the top of branches (at the order level) in purple and green, respectively. *n* indicates the number of species in each order used in the analysis and the size of the triangle is proportionate to this number. The order Chiroptera is filled with black. MRCA, most recent common ancestor. In total, 11 miRNA families were contracted in the ancestral bat branch, with no evidence of expansion. Between 3 and 21 miRNA families were contracted in the different bat species and between 2 and 7 were gained (Supplementary Fig. 9). This pattern of miRNA expansion and contraction in bats is not unusual compared to that observed in other lineages. **b**, Alignment of mature miR-337-3p sequences across mammals, with human as reference sequence. The genomes of all 48 mammalian species (Supplementary Table 1) were screened for the presence of miR-337 on the basis of its sequence similarity and secondary structure using the Infernal pipeline. To confirm that the seed region of miR-337-3p is conserved widely in bats, we also included four previously reported Illumina bat genomes (*Myotis brandtii*, *Myotis davidii*, *Eptesicus fuscus* and *Pteropus alecto*) alongside the six Bat1K genomes we sequenced. miR-337 was not detected in cow, pig and dog genomes by our pipelines, which are therefore not represented in this figure. Two changes are present in the seed region of miR-337-3p, the combination of which is uniquely found in bats, and not in other mammals. **c**, Cumulative expression of miR-337-3p in the six Bat1K species based on small RNA-seq data. Cumulative abundance of conserved miRNA from brain, liver and kidney for each bat species is reported as RPM (reads per million mapped reads) and reported as individual data points to show the dispersion of the data. Box plots extend from the 25th to 75th percentiles, the central line represents the median value and whiskers extend to a maximum of 1.5× the inter quartile range beyond the box. *n* indicates the number of conserved miRNA detected in each species. The abundance of miR-337-3p is highlighted in red. miR-337-3p is consistently and highly expressed across these six bat species, highlighting its potential importance in bats and suggesting that alteration to this miRNA may have an effect in bat biology. **d**, Sequence changes in the miR-337-3p seed region are predicted to alter the repertoire of its gene targets in bats. Following predictions of miRNA binding sites in the 3′ UTRs of humans and bats, respectively, we used Gene Ontology (GO) enrichment (via DAVID) to understand the biological processes regulated by the human and bat miR-337-3p. miR-337-3p was predicted to regulate distinct biological processes in bat and human as a result of the two sequence changes found in the seed region. In bats, novel GO categories were enriched including developmental, rhythmic and neuronal processes. Target lists used for analyses were *n* = 1,159 for bat and *n* = 601 for human, and background lists were *n* = 13,083 for both. Corrected *P* values were generated by DAVID via a modified Fisher’s exact test (EASE score) and Benjamini multiple testing correction.

The role of noncoding RNAs in driving phenotypic adaptation has recently been established^68^, but little is known about their evolution in bats. We comprehensively annotated noncoding RNAs in our bat genomes, and screened for variation in noncoding RNA by comparing our 6 bat species with 42 other mammals (Fig. 4a, Supplementary Note 5.1). We found that nearly all of the annotated noncoding RNA genes are shared across all six bat genomes (Supplementary Fig. 8), and between bats and other mammals (for example, 95.8–97.4% are shared between bats and humans). Given the importance of microRNAs (miRNAs) as developmental and evolutionary drivers of change^69^, we specifically investigated the evolution of families of miRNA genes. We identified 286 conserved miRNA gene families across all mammals (Supplementary Table 13), 11 of which were significantly contracted (false discovery rate < 0.05) (Extended Data Fig. 9a, Supplementary Fig. 9), and 13 of which were lost, in the ancestral bat branch (Supplementary Figs. 10, 11, Supplementary Note 5.2)—a pattern comparable to that of other mammal lineages (Extended Data Fig. 9a).

Next, we investigated the evolution of single-copy miRNA genes. Alignments of 98 highly conserved, single-copy miRNAs identified across the 6 bat and 42 other mammalian genomes revealed that one miRNA (miR-337-3p) had unique variation in the seed region in bats, as compared to other mammals (Fig. 4b, Extended Data Fig. 9b). We generated libraries for small RNA-seq from the brain, liver and kidney across the six bat species and showed that miR-337-3p is pervasively expressed (Extended Data Fig. 9c). Because miRNA seed sequences are the strongest determinant of target specificity, these seed changes are expected to alter the repertoire of sequences targeted by miR-337-3p in bats. Indeed, reporter assays (Supplementary Note 5.4, Supplementary Table 14) revealed that bat miR-337-3p strongly repressed the expression of its cognate bat target sequence but had no effect on the human site (and vice versa) (Fig. 4c), which demonstrates that the bat-specific seed sequence changes alter miR-337-3p binding specificity. We further explored whether this difference in binding specificity changes the set of target genes regulated, and found that bat and human miR-337-3p are predicted to regulate a distinct spectrum of gene targets (Supplementary Tables 15, 16, Supplementary Note 5.3). Gene Ontology enrichment analysis of these target gene sets suggests a shift towards regulation of developmental, rhythmic, synaptic and behavioural gene pathways in bats (Extended Data Fig. 9d), pointing to a marked change in processes regulated by miR-337-3p in this clade.

In addition to losses and variation, continuous miRNA innovation has previously been suggested to act as a key player in the emergence of increasing organismal complexity in eukaryotes^68^. To identify novel miRNAs (defined as having a novel seed sequence) that evolved in bats, we screened for novel sequences in the small RNA libraries from all six bat species (Supplementary Table 17, Supplementary Note 5.3). This expression analysis revealed 122–261 novel miRNAs across the 6 bat genomes, with only a small number being shared across 2 or more bats (Supplementary Fig. 12). From these, we identified 12 novel miRNAs that are present in the genome of all 6 bat species and that are also without apparent homologues in other mammals (Supplementary Table 18). To test whether these candidates are functional miRNAs, we selected the top three candidates (Supplementary Table 18, Supplementary Note 5.3), and experimentally tested their ability to regulate an ideal target sequence in reporter assays (Supplementary Table 14). Two of the three miRNAs we tested (miR-19125 and miR-6665) were able to regulate their targets, which shows that they are actively processed by endogenous miRNA machinery, loaded onto the RNA-induced silencing complex and able to repress target mRNAs (Fig. 4d). Thus, miR-19125 and miR-6665 represent true miRNAs that are evolutionary novelties in bats. Taken together, these data demonstrate innovation in the bat lineage, both in miRNA seed sequence and novel miRNA emergence. Further detailed mechanistic studies are required to determine the role of these miRNAs in bat physiology and evolution.

All of the results described here are supported by additional material that can be found in the Supplementary Methods, Supplementary Notes 1–5, Supplementary Tables 1–46, Supplementary Figs. 1–20 and Supplementary Data 1–3.

## Conclusion

We have generated chromosome-level, near-complete assemblies of six bat species that represent diverse chiropteran lineages. Using the comprehensive annotations of our bat genomes together with phylogenomic methodologies, we address the evolutionary origin of bats within Laurasiatheria and resolve bats as the sister taxa to Fereuungulata. Our conservative genome-wide screens investigating gene gain, loss and selection revealed novel candidate genes that are likely to contribute tolerance to viral infections among bats. Consistent with this finding, we also found that bat genomes contain a high diversity of endogenized viruses. We also uncovered genes involved in hearing that exhibit mutations specific to laryngeal-echolocating bats and ancestral patterns of selection. If future experiments show that these changes are indeed related to hearing, this would support a single ancestral origin of laryngeal echolocation and its subsequent loss in pteropodid bats. Finally, we identified and experimentally validated miRNAs that are evolutionary novelties or that carry bat-specific changes in their seed sequence. Changes in these important regulators of gene expression may have contributed to changes in developmental and behavioural processes in bats.

These high-quality bat genomes, together with future genomes, will provide a rich resource to address the evolutionary history and genomic basis of bat adaptations and biology, which is the ultimate goal of Bat1K^1^. These genomes enable a better understanding of the molecular mechanisms that underlie the exceptional immunity and longevity of bats, allowing us to identify and validate molecular targets that ultimately could be harnessed to alleviate human ageing and disease. For example, we predict that our reference-quality bat genomes will be tools that are heavily relied upon in future studies focusing on how bats tolerate coronavirus infections. This is of particular global relevance given the current pandemic of coronavirus disease 2019 (COVID-19), and ultimately may provide solutions to increase human survivability—thus providing a better outcome for this, and future, pandemics.

## Methods

No statistical methods were used to predetermine sample size. The experiments were not randomized and investigators were not blinded to allocation during experiments and outcome assessment.

### Genome sequencing

Genome sequencing was performed following the protocols of the Bat1K genome consortium (http://bat1k.com) in coordination with the Vertebrate Genome Project (https://vertebrategenomesproject.org/)^70^. Ultralong and long genomic DNA from various bat tissues was isolated either (a) by phenol–chloroform based DNA clean-up and precipitation, (b) with the Qiagen MagAttract HMW DNA kit or (c) with the agarose-plug-based Bionano Prep Animal tissue kit following the manufacturer’s instructions. The fragment size of all genomic DNAs was controlled by pulse-field gel electrophoresis before library construction. Size-selected PacBio CLR libraries of at least 20 kb in size were run on the SEQUEL system with 10-h movie times. For Bionano optical mapping, genomic DNA was labelled following either the NLRS or the DLS protocol according to the manufacturer’s instructions. Labelled genomic DNAs were run on the Bionano Saphyr instrument to at least 100× genome coverage. Linked Illumina reads were generated with the 10x Genomics Chromium genome protocol according to the manufacturer’s instructions. These libraries were sequenced on short read Illumina devices with a 150-bp paired-end regime. Hi-C confirmation capture was performed by Phase Genomics, ARIMA Genomics or by applying the ARIMA Genomics Hi-C kit. High-quality RNA was extracted by using commercially available RNA isolation kits. Standard PacBio Iso-Seq SMRTbell libraries were sequenced on the SEQUEL device with 10-h or 20-h movie times. Details of DNA and RNA library preparation are described in Supplementary Note 1, and statistics of all data collected for each bat are provided in Supplementary Note 2.1.

### Genome assembly

To reconstruct each genome, we first assembled the Pacbio reads ≥ 4 kb in length into contigs with our custom assembler DAmar, which outputs a set of ‘primary’ contigs that are guaranteed not to be a haplotype variant of a segment of another primary contig (called an ‘alternate’ contig). Consensus sequences of primary contigs were produced with two rounds of Arrow. The 10x data were subsequently used to both polish the consensus sequence further and to maximally phase heterozygous haplotype variation, followed by selecting one haplotype for each phased block arbitrarily. Bionano data were assembled into optical maps with Bionano Solve, which were used to scaffold the primary contigs and occasionally break a misjoined sequence contig. Finally, using Salsa2, the Hi-C data were used to scaffold the data into chromosome-spanning scaffolds. Measurements of karyotype images were used to assess whether scaffolds lengths resemble chromosome lengths.

To assess genome completeness, we used BUSCO (version 3)^22^ with the mammalian (odb9) protein set, applied both to our assemblies and our gene annotations. To assess completeness in noncoding regions, we used Blat (v.36x2)^71^ with sensitive parameters to determine how many of 197 nonexonic ultraconserved elements^20^ align at ≥ 85% identity.

### Gene annotation

To comprehensively annotate genes, we integrated different evidence. First, we used GenomeThreader (v.1.7.0)^72^ to align protein and RNA transcript sequences from NCBI or Ensembl for one other closely related bat species that has annotated genes. Second, we projected genes contained in the human, mouse and *Myotis lucifugus* Ensembl 96 annotation^73^ and our *M*. *myotis* annotation to other bats. To this end, we generated whole-genome alignments as described in ref. ^74^ and used Tool to infer Orthologs from Genome Alignments (TOGA)—a method that identifies the co-linear alignment chain(s)^75^ aligning the putative orthologue using synteny and the amount of intronic/intergenic alignments—and annotated genes with CESAR 2.0^76^ in multi-exon mode. Third, we generated de novo gene predictions by applying Augustus^77^ in single-genome mode with a bat-specific gene model trained by BRAKER (v.2.1)^78^ and extrinsic evidence provided as hints. In addition, we applied Augustus in comparative mode to a multiple genome alignment generated by MultiZ (v.11.2). Fourth, we used transcriptomic data from both publicly available data sources and our own Illumina short read RNA-seq data. Additionally, we generated PacBio long-read RNA sequences (Iso-Seq) from all six species to capture full-length isoforms and accurately annotate untranslated regions (UTRs). RNA-seq reads were stringently mapped using HISAT2 (v.2.0.0)^79^. Transcriptomic data were processed using TAMA^80^. All transcriptomic, homology-based and ab initio evidence were integrated into a consensus gene annotation using EVidenceModeller (v.1.1.1)^81^. High-confidence transcripts and TOGA projections were added if they provided novel splice site information.

### Transposable elements

We annotated each genome for transposable elements (TEs) following previous methods^82^ that incorporate de novo TE discovery with RepeatModeler^83^ followed by manual curation of potentially novel TEs (putative elements with mean K2P divergences <6.6% from the relevant consensus). Starting consensus sequences were also filtered for size (>100 bp). To classify final consensus sequences, each TE was examined for structural hallmarks and compared to online databases: blastx to confirm the presence of known ORFs in autonomous elements, RepBase (v.20181026) to identify known elements and TEclass^84^ to predict TE type. Finally, duplicates were removed via the program cd-hit-est (v.4.6.6)^85,86^ if they did not pass the 80-80-80 rule as described in ref. ^87^. The final de novo curated elements were combined with a vertebrate library of known TEs in RepBase (v.20181026) (Supplementary Data 1) and RepeatMasker analysis of the bats and seven mammalian outgroups were examined. Full details of these methods are available in Supplementary Note 3.3.

### Phylogenomics

Human transcripts were projected to 41 additional mammal species resulting in 12,931 genes classified as 1:1 orthologues by TOGA (Supplementary Data 2). Non-homologous segments were trimmed and CDS sequences were aligned. The best-fit model of sequence evolution for each alignment was found and used to infer a maximum likelihood (ML) gene tree using IQTREE^28^. Individual gene alignments were also concatenated into a partitioned supermatrix, which was used to estimate the mammalian species tree. Branch support for this tree was determined using 1,000 bootstrap replicates. This species tree was rooted on Atlantogenata and used to determine the position of Chiroptera position within Laurasiatheria. Individual gene trees were compared to the species tree using Robinson–Foulds (RF) distances^30^. Phylogenomic signal within our genomes was further explored by estimating the ML support of each protein-coding gene for the 15 possible bifurcating laurasiatherian topologies involving four clades, with Eulipotyphla as the outgroup. An additional supermatrix, consisting of 10,857 orthologous conserved noncoding elements (CNEs), was generated and explored using the aforementioned methods.

To assess whether model misspecification or loss of historic signal affected our data, all 12,931 alignments were examined for evidence of violating the assumptions of evolution under homogeneous conditions and a decay of signal owing to multiple substitutions. A total of 488 gene alignments, containing all 48 taxa, were considered optimal for phylogenetic analysis under these conditions. These data were explored using the methods above, and the SVDquartets single-site coalescence-based method^37^, as an alternative to concatenation. A full description of all phylogenetic methods is available in Supplementary Note 4.2.

### Gene selection, loss and gain

We screened all 12,931 orthologous genes for signatures of positive selection on the stem Chiroptera branch using the best supported mammalian phylogeny and two state-of-the-art methods, aBSREL implemented in HyPhy^39^ and codeml in PAML^40^. We required a HyPhy false discovery rate < 0.05 (using the Benjamini–Hochberg procedure to correct for 12,931 statistical tests) and a codeml *P* < 0.05. To increase the sensitivity in detecting positive selection in genes relevant for prominent bat traits, we also performed a screen considering 2,453 candidate genes associated with longevity, immunity or metabolism. Genes showing evidence of positive selection were subsequently explored using protein structure prediction and modelling methods (Supplementary Data 3). To systematically screen for gene losses, we used a previously developed approach^53^ (Supplementary Note 4.5), and required that less than 80% of the ORF was intact in all six bats, excluding genes classified as lost in more than 20% of non-Chiroptera Laurasiatherian mammals contained in our 120-mammal multiple genome alignment^88^ (Supplementary Note 4.5). We confirmed the presence of inactivating mutations in independently sequenced bat species. To investigate expansions and contractions of protein families, we used CAFE^89^ with a false discovery rate < 0.05 cut-off. As input for CAFE, we clustered Ensembl-annotated proteins into families using POrthoMCL^90^ and the PANTHER Database (v.14.0)^91^ and our ultrametric time tree, generated using r8s.

### Integrated viruses in bat genomes

The six bat genomes and seven additional mammalian genomes were inspected for the presence of EVEs and ERVs. Potential integrations were identified using local BLAST^92^ with 14 probes for the viral proteins Gag, Pol and Env from each genus of *Retroviridae* for ERVs; tblastn^92^ of an established comprehensive library^62^ of non-retroviral proteins identified integrations of other viral types. Reciprocal blast of identified regions was used to identify viral family (for EVEs) or closest retroviral genus (for ERVs). Regions for each viral protein family passing quality thresholds were aligned using MUSCLE within Aliview^93^. A phylogenetic tree for the identified retroviral pol-like sequences from the six bat genomes and probes was then reconstructed using RAxML with the VT + G model^94^.

### Evolution of noncoding genomic regions

Conserved noncoding RNA genes were annotated using the Infernal pipeline^95^. To gain insights into the evolution of conserved miRNA families along the bat lineages, we performed two analyses that investigate (i) expansion or contraction of members with miRNA gene families, and (ii) gain or loss of miRNA gene families. To explore variation in miRNA sequence unique to bats, we aligned and investigated single-copy miRNA genes across these 48 taxa. We developed a pipeline to predict the gene targets of candidate miRNAs and the biological processes in which they are potentially engaged. To identify novel miRNAs evolved in bats, we sequenced small RNA libraries from brain, kidney and liver for all six bat species using Illumina miRNA-seq. We carried out a comprehensive pipeline to identify novel miRNA commonly shared by the ancestral bat lineage. We further used luciferase assays^96,97^ to test the functionality of candidate miRNAs in vitro. A full description is provided in Supplementary Note 5.

### Reporting summary

Further information on research design is available in the Nature Research Reporting Summary linked to this paper.

## Online content

Any methods, additional references, Nature Research reporting summaries, source data, extended data, supplementary information, acknowledgements, peer review information; details of author contributions and competing interests; and statements of data and code availability are available at

## Supplementary information

Supplementary Information This file contains Supplementary Notes including Supplementary Materials, Supplementary Methods, Supplementary Figures 1-6, 8-20, Supplementary Tables 1-5,11,14-15,17,19-34,37,39, as well as a series of findings in addition to those shown in the main manuscript.

Reporting Summary

Supplementary Figure 7 | Reconstructed phylogenetic tree of viral pol sequences found in six species of bats Each species used in the ERV search is marked with a colour: *Phyllostomus* (navy blue), *Myotis* (green), *Pipistrellus* (orange), *Rhinolophus* (yellow), *Molossus* (light blue), *Rousettus* (pink) and reference sequences (black). Bootstrap values are shown where the values are ≥ 70%. The tips of the phylogeny are labelled with the species name, position in the reference genomes and the direction of the sequences (N- negative/P- positive).

Supplementary Tables This file contains Supplementary Tables 6-10,12-13,16,18,35-36,38,40-46. The title and caption of each Supplementary Table can be found in the Supplementary Information file.

Supplementary Data File 1 *De novo* curated transposable elements combined with a vertebrate library of known TEs in RepBase.

Supplementary Data File 2 Alignments of 12,931 protein-coding genes across 48 mammals.

Supplementary Data File 3 Predicted 3D protein structures of candidate genes.

## Data Availability

All data generated or analysed during this study are included in the Article and its Supplementary Information. All genomic and transcriptomic data are publicly available for visualization via the open-access Bat1K genome browser (https://genome-public.pks.mpg.de) and for download at https://bds.mpi-cbg.de/hillerlab/Bat1KPilotProject/. In addition, the assemblies have been deposited in the NCBI database under BioProject PRJNA489245 and GenomeArk (https://vgp.github.io/genomeark/). Accession numbers for all the miRNA-seq and RNA-seq data used in this study can be found in Supplementary Tables 17 and 34, respectively.

## Code availability

All custom code has been made available on GitHub at https://github.com/jebbd/Bat1K and https://github.com/MartinPippel/DAmar.
