# Supplementary material for: Six reference-quality genomes reveal evolution of bat adaptations
Source: Nature. 2020 Jul 22;583(7817):578–84. doi: 10.1038/s41586-020-2486-3 (PMC8075899; doi:10.1038/s41586-020-2486-3)
Supplement: Supplementary file 1 — This file contains Supplementary Notes including Supplementary Materials, Supplementary Methods, Supplementary Figures 1-6, 8-20, Supplementary Tables 1-5,11,14-15,17,19-34,37,39, as well as a series of findings in addition to those shown in the main manuscript. [file 41586_2020_2486_MOESM1_ESM.pdf]

nature

https://doi.org/10.1038/s41586-020-2486-3

Supplementary information

Six reference-quality genomes reveal evolution of bat adaptations

In the format provided by the authors and unedited

David Jebb, Zixia Huang, Martin Pippel, Graham M. Hughes, Ksenia Lavrichenko, Paolo Devanna, Sylke Winkler, Lars S. Jermiin, Emilia C. Skirmuntt, Aris Katzourakis, Lucy Burkitt-Gray, David A. Ray, Kevin A. M. Sullivan, Juliana G. Roscito, Bogdan M. Kirilenko, Liliana M. Dávalos, Angelique P. Corthals, Megan L. Power, Gareth Jones, Roger D. Ransome, Dina K. N. Dechmann, Andrea G. Locatelli, Sébastien J. Puechmaille, Olivier Fedrigo, Erich D. Jarvis20,, Michael Hiller✉, Sonja C. Vernes✉, Eugene W. Myers✉ & Emma C. Teeling✉

Supplementary Information

Title: Six reference-quality genomes reveal evolution of bat adaptations

David Jebb^[1,2,3], Zixia Huang^[4], Martin Pippel^[1,3], Graham M. Hughes[4], Ksenia Lavrichenko[5], Paolo Devanna[5], Sylke Winkler[1], Lars S. Jermiin[4,6,7], Emilia C. Skirmuntt[8], Aris Katzourakis[8], Lucy Burkitt-Gray[9], David A. Ray[10], Kevin A. M. Sullivan[10], Juliana G. Roscito[1,2,3], Bogdan M. Kirilenko[1,2,3], Liliana M. Dávalos[11,12], Angelique P. Corthals[13], Megan L. Power[4], Gareth Jones[14], Roger D. Ransome[14], Dina Dechmann[15,16,17], Andrea G. Locatelli[4], Sebastien J. Puechmaille[18,19], Olivier Fedrigo[20], Erich D. Jarvis[21,22], Michael Hiller*[1,2,3], Sonja C. Vernes*[5,23], Eugene W. Myers*[1,3,24], Emma C. Teeling*[4]

[1]Max Planck Institute of Molecular Cell Biology and Genetics, Dresden, Germany
[2]Max Planck Institute for the Physics of Complex Systems, Dresden, Germany
[3]Center for Systems Biology Dresden, Dresden, Germany
[4]School of Biology and Environmental Science, University College Dublin, Dublin, Ireland
[5]Neurogenetics of Vocal Communication Group, Max Planck Institute for Psycholinguistics, Nijmegen, The Netherlands
[6]Research School of Biology, Australian National University, Canberra, ACT, Australia
[7]Earth Institute, University College Dublin, Dublin, Ireland
[8]Peter Medawar Building for Pathogen Research, Department of Zoology, University of Oxford, Oxford, United Kingdom
[9]Conway Institute of Biomolecular and Biomedical Science, University College Dublin, Dublin, Ireland
[10]Department of Biological Sciences, Texas Tech University, Lubbock, USA
[11]Department of Ecology and Evolution, Stony Brook University, Stony Brook, Stony Brook, USA
[12]Consortium for Inter Disciplinary Environmental Research, Stony Brook University, Stony Brook, USA
[13]Department of Sciences, John Jay College of Criminal Justice, New York, USA
[14]School of Biological Sciences, University of Bristol, Bristol, United Kingdom
[15]Department of Migration and Immuno-Ecology, Max Planck Institute of Animal Behavior, Radolfzell, Germany
[16]Department of Biology, University of Konstanz, Konstanz, Germany
[17]Smithsonian Tropical Research Institute; Panama City, Panama
[18]ISEM, University of Montpellier, Montpellier, France
[19]Zoological Institute and Museum, University of Greifswald, Greifswald, Germany
[20]Vertebrate Genomes Laboratory, The Rockefeller University, New York, NY, USA
[21]Laboratory of Neurogenetics of Language, The Rockefeller University, New York, NY, USA
[22]Howard Hughes Medical Institute, Chevy Chase, MD, USA
[23]Donders Institute for Brain, Cognition and Behaviour, Nijmegen, The Netherlands
[24]Faculty of Computer Science, Technical University Dresden, Dresden, Germany

This Supplementary Information includes:

Supplementary Methods and Results

Supplementary Figures 1-6, 8-20

Supplementary Tables 1-5, 11, 14-15, 17, 19-34, 37, 39

The materials presented as separate files include:

Supplementary Figure 7

Supplementary Tables 6-10, 12-13, 16, 18, 35-36, 38, 40-46

Supplementary Data File 1-3

Table of Contents

1. Samples, DNA extraction and genome sequencing

1.1. Ethical statements and sample storage
1.2. Genomic DNA isolation and library preparation (PacBio, Illumina, Hi-C, 10x Genomics and Bionano)
1.2.1. Phenol-chloroform extraction of genomic DNA
1.2.2. Bionano agarose plug based isolation of megabase-size gDNA
1.2.3. PacBio long insert library preparation
1.2.4. Bionano optical mapping of megabase-size gDNA
1.2.5. 10x linked Illumina reads
1.2.6. Hi-C confirmation capture
1.3. Pacific Biosciences long reads transcriptome sequencing (Iso-seq)
1.3.1. Total RNA extraction
1.3.2. Library preparation
1.3.3. Sequencing

2. Genome assembly

2.1. Data sets and assembly inputs
2.1.1. PacBio reads
2.1.2. 10x Illumina read counts
2.1.3. Bionano restriction mapped molecules
2.1.4. Hi-C Illumina read pairs
2.2. Assembly pipeline
2.2.1. Setup phase
2.2.2. Read patching
2.2.3. *De novo* assembly
2.2.4. Error polishing
2.2.5. Haplotype phasing
2.2.6. Bionano scaffolding
2.2.6.1. *De novo* assembly
2.2.6.2. Hybrid scaffolding
2.2.7. Hi-C scaffolding
2.2.8. Manual curation
2.3. Assembly results

3. Genome annotation

3.1. Protein-coding gene annotation
3.1.1. Overview
3.1.2. TOGA projections
3.1.3. Alignments of protein and cDNA sequences of related bat species
3.1.4. Transcriptome data
3.1.5. *De novo* gene prediction
3.1.6. Integrating all gene evidence into a final gene annotation
3.1.7. Prediction of 3’UTR sequences from Iso-seq transcripts

3.1.8. Filtering transcripts for coding potential and assigning gene symbols
3.1.9. Computing annotation completeness
3.2. Analysis of ultraconserved elements
3.3. Repetitive element annotation
3.3.1. TE results
3.4. Annotation and analysis of endogenous viral elements (EVE) and endogenous retrovirus (ERV)
3.4.1. EVE annotation and analysis
3.4.2. ERV annotation and analysis
3.4.3. EVE and ERV results

4. Genome evolution

4.1. Identification and alignment of one-to-one orthologs across Placentalia
4.2. Phylogenetic analysis
4.2.1. Phylogenetic inference and divergence time estimation
4.2.2. Exploring the impact of misalignment and incorrect homology on supermatrix topology
4.3. Selection test
4.3.1. Genome-wide screen for signatures of positive selection
4.3.2. Candidate genes and selection tests
4.3.3. Selection in non-chiropteran branches
4.4 *In silico* analyses of protein structure
4.5. Systematic screen for gene losses
4.6. Protein family evolution
4.6.1. Evolution of the *APOBEC3* gene cluster

5. Evolution of non-coding genomic regions

5.1. Annotation of conserved non-coding RNA genes
5.2. The evolution of conserved miRNA gene families
5.2.1. miRNA family expansion and contraction
5.2.2. miRNA gene gain and loss
5.2.3. Single-copy miRNA alignments across 48 mammals
5.3. Novel microRNAs that evolved in bats
5.3.1. Small RNA Illumina sequencing from brain, kidney and liver
5.3.2. miRNA profiling pipeline
5.3.3. Identification of known and novel miRNAs in each bat species
5.3.4. miRNA evolution in bats
5.3.5. 3'UTR and miRNA target prediction
5.4. Functional validation of novel miRNAs and their regulatory gene targets

1. Samples, DNA extraction and genome sequencing

Bat species were chosen to enable capture of the major ecological trait space and life histories observed in bats while representing deep phylogenetic divergences. These six bat species belong to five families that represent key evolutionary clades, unique adaptations and span both major lineages in Chiroptera estimated to have diverged ~64 MYA[1]. In the suborder Yinpterochiroptera we sequenced *Rhinolophus ferrumequinum* (Greater horseshoe bat; family Rhinolophidae) and *Rousettus aegyptiacus* (Egyptian fruit bat; Pteropodidae), and in Yangochiroptera we sequenced *Phyllostomus discolor* (Pale spear-nose bat; Phyllostomidae), *Myotis myotis* (Greater mouse-eared bat; Vespertilionidae), *Pipistrellus kuhlii* (Kuhl's pipistrelle; Vespertilionidae) and *Molossus molossus* (Velvety free-tailed bat; Molossidae) (Supplementary Table 1).

1.1 Ethical statements and sample storage

The ethical statements of collecting and processing tissue samples for each species are listed as follows:

Myotis myotis: All procedures were carried out in accordance with the ethical guidelines and permits (AREC-13-38-Teeling) delivered by the University College Dublin and the Préfet du Morbihan, awarded to Emma Teeling and Sébastien Puechmaille respectively. A single *M. myotis* individual (MMY2607) was euthanized at a bat rescue centre given that she was missing all fingers and plagiopatagium on the left wing, and dissected. ***Rhinolophus ferrumequinum***: All the procedures were conducted under the license (Natural England 2016-25216-SCI-SCI) issued to Gareth Jones. The individual bat died unexpectedly and suddenly during sampling and was dissected immediately. ***Pipistrellus kuhlii***: The sampling procedure was carried out following all the applicable national guidelines for the care and use of animals. Sampling was done in accordance with all the relevant wildlife legislation and approved by the Ministry of Environment (Ministero della Tutela del Territorio e del Mare, Aut.Prot. N˚: 13040, 26/03/2014). ***Molossus molossus***: All sampling methods were approved by the Ministerio de Ambiente de Panamá (SE/A-29-18) and by the Institutional Animal Care and Use Committee of the Smithsonian Tropical Research Institute (2017-0815-2020). ***Phyllostomus discolor***: *P. discolor* bats originated from a breeding colony in the Department Biology II of the Ludwig-Maximilians-University in Munich. Approval to keep and breed the bats was issued by the Munich district veterinary office. Under German Law on Animal Protection, a special ethical approval is not needed for this procedure, but the sacrificed animal was reported to the district veterinary office. ***Rousettus aegyptiacus***: Egyptian fruit bats originated from a breeding colony at University of California (UC), Berkeley. All experimental and breeding procedures were approved by the UC Berkeley Institutional care and use committee (IACUC).

Sampled tissues were snap-frozen in liquid nitrogen immediately after dissection and were kept at -80ºC until further processed. Detailed information of samples is available in Supplementary Table 19.

1.2 Genomic DNA isolation and library preparation (PacBio, Illumina, Hi-C, 10x Genomics and Bionano)

1.2.1 Phenol-chloroform extraction of genomic DNA

Snap-frozen tissues of all bat species were pulverized into a fine powder in liquid nitrogen. Powdered muscle tissue was lysed overnight at 55ºC in high-salt tissue lysis buffer (400 mM NaCl, 20 mM Tris base pH 8.0. 30 mM EDTA pH 8.0, 0.5% SDS, 100 µg/ml Proteinase K), and powdered lung tissue was lysed overnight in Qiagen G2 lysis buffer (Cat. No. 1014636, Qiagen, Hilden, Germany) containing 100 µg/ml Proteinase K at 55ºC. RNA was removed by incubating in 50 µg/ml RNase A for 1 hour at 37ºC. High molecular weight genomic DNA (HMW gDNA) was purified with two washes of Phenol-Chloroform-IAA equilibrated to pH 8.0, followed by two washes of

Chloroform-IAA, and precipitated in ice-cold 100% Ethanol. Filamentous HMW gDNA was either spooled with shepherds' hooks or collected by centrifugation. HMW gDNA was washed twice with 70% Ethanol, dried for 20 minutes at room temperature and eluted in TE. DNA molecule length was between 50 and 300 kb as shown by pulse field gel electrophoresis (PFGE) (Pippin Pulse, SAGE Science, Beverly, MA).

1.2.2 Bionano agarose plug based isolation of megabase-size gDNA

Megabase-size gDNA was extracted according to the Bionano Prep™ Animal tissue DNA isolation soft tissue protocol (Document number 30077, Bionano, San Diego, CA) for liver tissue and according to the Bionano Prep™ Animal tissue DNA isolation fibrous tissue protocol (Document number 30071) for lung, muscle, and heart tissues. Fibrous tissues were mildly fixed in 2% formaldehyde and homogenized. Nuclei were enriched by centrifugation. Soft tissues were homogenized in a tissue grinder directly followed by a mild ethanol fixation. Nuclei or homogenized tissues were embedded into agarose plugs and treated with Proteinase K and RNase A. Genomic DNA was extracted from agarose plugs and purified by drop dialysis against 1x TE. PFGE revealed mega-size DNA molecule length of 100 kb up to 500 kb. For *P. discolor*, additionally we extracted DNA using the Qiagen MagAttract HMW DNA kit (according to manufacturer guidelines) using 25-30 mg of tissue. The information regarding gDNA extraction is detailed in Supplementary Table 20.

1.2.3 PacBio long insert library preparation

Long insert libraries were prepared as recommended by Pacific Biosciences (PacBio, Menlo Park, CA) according to the guidelines for preparing size-selected 20 kb SMRTbell™ templates. The Megaruptor™ device (Diagenode, Liege, Belgium) was used for shearing 10-20 µg genomic DNA following the manufacturer's instructions. PacBio SMRTbell™ libraries were size-selected for large fragments using the SAGE BluePippin™ devise. SMRT sequencing was done on the SEQUEL system using sequencing chemistries 1.0 to 2.0. Movie time was 10 hours for all SMRT cells. The detailed information regarding PacBio sequencing statistics is available in Supplementary Table 21.

1.2.4 Bionano optical mapping of megabase-size gDNA

Megabase-size gDNA of *P. discolor* and *R. ferrumequinum* was labelled as described in the Bionano Prep™ Labeling NLRS protocol (Document Number 30024). DNA was tagged with two different enzymes each (BSPQI and BSSSI) to achieve the maximum labelling information. Labelled gDNA of these species was run on the Saphyr platform at the Vertebrate Genome Lab at the Rockefeller University. Megabase-size gDNA of the other four species (*M. molossus*, *M. myotis*, *P. kuhlii*, and *R. aegyptiacus*) was labelled as described in the Bionano Prep direct label and stain (DLS) protocol (Document number 30206). These DNAs were tagged with the nicking-free DLE enzyme. One flow cell of *M. molossus, M. myotis*, and *P. kuhlii* labelled gDNA was run on the Bionano Saphyr instrument at the MPI for Evolutionary Biology in Ploen, Germany. One flow cell of labelled *R. aegyptiacus* gDNA was run on the Bionano Saphyr instrument at the DRESDEN concept Genome Center (DcGC), Dresden, Germany. For all six species, at least 100X raw genome coverage was achieved.

1.2.5 10x linked Illumina reads

Linked Illumina reads were generated using the 10x Genomics Chromium™ genome application following the Genome Reagent Kit Protocol v2 (Document CG00043, Rev B, 10x Genomics, Pleasonton, CA). In brief, 1 ng of long or megabase-size genomic DNA was partitioned across 1 Million Gel bead-in-emulsions (GEMS) using the Chromium™ device. Individual gDNA molecules were amplified in these individual GEMS in an isothermal incubation using primers that contain a specific 16 bp 10x barcode and the Illumima® R1 sequence. After breaking the emulsions, pooled amplified barcoded fragments were purified, enriched and went into Illumina sequencing library preparation as described in the protocol. Pooled Illumina libraries were sequenced to at least

40X genome coverage on an Illumina HiSEQ4000 or an Illumina NovaSeq instrument at the MPI of Molecular Genetics in Berlin, Germany, using the 2x 150 cycles paired-end regime plus 8 cycles of i7 index. The 16 bp 10x barcodes allow the reconstitution of long DNA molecules by linking reads that carry the identical barcode. The detailed information regarding 10x Genomics sequencing is available in Supplementary Table 22.

1.2.6 Hi-C confirmation capture

Hi-C confirmation capture of *M. myotis*, *P. kuhlii*, and *R. ferrumequinum* was outsourced to Phase Genomics in Seattle, WA. Hi-C confirmation capture of *P. discolor* was done by Arima Genomics in San Diego, CA. For *M. molossus* and *R. aegyptiacus*, Hi-C confirmation capture and Illumina sequencing was done at the DcGC by applying the Arima Genomics Hi-C kit and sequencing on the Illumina Nextseq device.

1.3 Pacific Biosciences long read transcriptome sequencing (Iso-seq)

1.3.1 Total RNA extraction

The overview of tissues and RNA samples used for Iso-seq is available in Supplementary Table 23. All tissues were lysed in TRIzol reagent (No. 15596-018, Carlsbad, CA). Total RNA extraction and purification was conducted either with a standard chloroform-isopropanol extraction protocol or using the QIAGEN RNeasy kit (Cat. No. 74104) or the ReliaPrep™ RNA cell miniprep kit (Cat. No. Z6110, Promega Madison, WI). The quality and quantity of all RNAs were measured using a Bioanalyzer 2100 or an Agilent 2200 Tapestation (Aligent Technologies, Santa Clara, CA). RIN values are given in Supplementary Table 23.

1.3.2 Library preparation

PacBio Iso-seq libraries were prepared according to the ‘Procedure & Checklist - Iso-Seq™ Template Preparation for Sequel® Systems’ (PN 101-070-200 version 05) without Blue Pippin size selection. Briefly, cDNA was reversely transcribed using the SMRTer PCR cDNA synthesis kit (Clontech, Mountain View, CA) from 1 µg total RNA and amplified in a large-scale PCR. Two fractions of amplified cDNA were isolated using either 1x AMPure beads or 0,4x AMPure beads. Both fractions were pooled equimolar and went into the Pacbio SMRTbell template preparation v1.0 protocol following the manufacturer’s instruction.

1.3.3 Sequencing

PacBio Iso-seq libraries were sequenced on the SEQUEL device with PacBio sequencing chemistry 3.0 and with 20 hours movie time. One SMRT cell was sequenced per Iso-seq library. Raw sequence yield (polymerase yield) for all Iso-seq libraries was between 18 and 32 Gb per SMRT with 624,989 to 732,879 reads per library. The *P. discolor* testes sample was sequenced on one SMRTcell using a 10-hour movie and chemistry 2.1, which resulted in 487,808 reads.

2. Genome assembly

2.1 Data sets and assembly inputs

The original data collection design was to produce 60X coverage in PacBio long reads, 50X in 10x Illumina read clouds, and 10X in Hi-C read pairs[2]. The idea was that the latter two technologies would be used for scaffolding contigs produced by an initial assembly of the PacBio reads into contigs. However, early on, it became clear that the yield of long read clouds with the 10x technology was very low. Even after switching to a plug-based DNA extraction method at a later

timepoint, the yield of long clouds, while better, was still not cost efficient. Therefore, we abandoned the idea of using 10x read cloud data for scaffolding, albeit this data was still very useful for base error correction and haplotype phasing, as each read cloud is itself phased. To compensate, and also in part based on our experience with the VGP project[3], we decided to generate a higher coverage in Hi-C read pairs for *P. discolor*, *R. aegyptiacus* and *M. molossus*. Furthermore, we decided to collect Bionano restriction mapped molecules and generate optical maps for all six bats since in the year after our initial proposal[2]. Bionano's optical map technology improved greatly in molecule length and has since proved very powerful for scaffolding. In addition, the increased Hi-C coverage also gave us more scaffolding power, to the extent that the largest scaffolds were effectively chromosomes. We describe in the subsections 2.1.1 – 2.1.4 each of the four data sets for each of the six bats. The genomic sequencing data of these 6 bat species are available in the NCBI BioProject PRJNA489245.

2.1.1 PacBio reads

The target coverage for long read sequencing was 60X. In Supplementary Table 24, we report statistics on all the raw data that we collected for each species, and all the data used for assembly which is the raw data except all those reads that were <4 kb in length. In the statistics for the raw data we did not count multiple reads of an insert in a given well, but only the longest read from each well. A gradual improvement is observed in yield per cell over the runtime of the project and the estimated coverage of the trimmed data is above or very near the 60X target for 5 of the 6 bats. The only exception is *M. molossus* with a trimmed data coverage of 52X; however, this species turned out to have an unexpectedly larger genome size of 2.3 Gb (versus ~2 Gb or less for all the others). Despite slightly lower read coverage, the PacBio reads for *M. molossus* are the longest (Extended Data Fig. 1a). The expected coverage reported is the total base pairs collected, divided by the *post hoc* genome size of the resulting assemblies. The data for *P. discolor* was created at Rockefeller and Duke University and the other five bats were sequenced in Dresden, Germany.

2.1.2 10x Illumina read counts

We collected about 50X Illumina reads organized into read clouds with the 10x Genomics technology[4] for *M. myotis*, *P. kuhlii*, and *R. ferrumequinum*. In this technology, a small number (*e.g.* 2-20) of ideally long molecules were isolated in an oil-immersion micro-well with a reagent payload that produces roughly 0.2-0.3X amplicons with the same barcode. The resulting library was then Illumina pair-read sequenced, resulting in "clouds" of reads with the same barcode. The reads were phased as the template was single stranded and it is noteworthy that a given cloud should map to a small number of regions whose size and number correspond to the molecules in the well. Locality information is thus rather indirect, but sufficient with large numbers of clouds to achieve moderately good assemblies[5].

We were expecting a large fraction of the clouds to be 100 kb or longer. The size distribution of the molecule lengths from which each cloud was derived cannot be measured directly. However, cloud reads can be mapped to the contigs produced by an initial assembly of the PacBio data in order to get a *post hoc* estimate of this distribution. This revealed that only 1% of the molecules were 100 kb or longer and most were much shorter. This can be seen clearly in Supplementary Figure 13, which plots the Nx values for the putative estimates of molecule length. Therefore, the coverage in long molecules was less than 1X and consequently this data provided very little scaffolding information. Given that the reads in a cloud must be inferred, they also tended to have a very high scaffolding error rate.

One could conjecture that the short molecule distribution was a protocol/lab error, but the data set produced for *P. discolor* by the VGL at Rockefeller University had the same characteristics (see Supplementary Table 25 and Supplementary Figure 13). Later in the project, when we started using DNA extracted with the Bionano plug-based method (Document number 30077, Bionano, San Diego, CA), the molecule length distribution improved significantly with a tail that put about 50% of the data in molecules above 100 kb. However, this is still a relatively low yield of long molecules

compared to the Bionano data which will be described in the Supplementary Note 2.1.3. In summary, while we produced ≥40X of 10x read clouds for all six bats, we only used this data for error polishing and phasing in our assembly pipeline described in Supplementary Note 2.2.

2.1.3 Bionano restriction mapped molecules

Since Bionano proved to be producing very long restriction-mapped molecules (100-300 kb on average), we begun to produce this data for all six bats. Supplementary Table 26 summarizes the gross statistics for each data set.

While one could use each molecule directly to scaffold contigs, we chose to first assemble each Bionano map using the company's restriction map assembler Solve (Document number 30205, Revision E, Bionano, San Diego, CA). This then gave us optical maps that we used in the sequence assembly pipeline. Supplementary Table 27 summarizes the aggregate statistics for the assemblies. The data sets of *P. discolor* and *R. ferrumequinum* were performed with two enzymes and therefore had a distinct optical map assembly for each enzyme. One should note that while the coverage in molecules was the lowest for *R. aegyptiacus*, the average and N50 map lengths were the highest. This indicates that coverage alone does not determine the degree of assembly and may be less important than the distribution of read lengths, which was the best for *R. aegyptiacus*.

2.1.4 Hi-C Illumina read pairs

Initially we contracted with Phase Genomics to produce 15X Hi-C data sets for *M. myotis*, *P. kuhlii* and *R. ferrumequinum*. Later in the project it became clear that Hi-C data is extremely well suited to give one the overall chromosomal view of a genome. Therefore, we increased the coverage of this data to >60X for the remaining three genomes, contracting one data set to Arima (*P. discolor*) and using the Arima kits in-house for the other two bats (*R. aegyptiacus*, *M. molossus*). Supplementary Table 28 shows the Hi-C sequencing statistics.

2.2 Assembly pipeline

De novo genome assembly was performed with DAmar (https://github.com/MartinPippel/DAmar). This assembler is based on an improved MARVEL assembler (https://github.com/schloi/MARVEL, commit ID: 5e17326)[6,7] and the integration of parts from the DAZZLER (DALIGNER commit ID: 233274a; DAMASKER commit ID: bc7e49c; DASCRUBBER commit ID: 3491b14; DAZZ_DB commit ID: 340fd89; DEXTRACTOR commit ID: 2f51ccb)[8] and the DACCORD code base (version: 0.0.14-release-20180525105343)[9,10].

To assemble the bat genomes, we performed the following steps: setup, PacBio read patching, assembly, error polishing, haplotype phasing, scaffolding and manual curation. Extended Data Fig. 1b shows a schematic overview of the assembly pipeline.

2.2.1 Setup phase

In the setup phase, PacBio reads were filtered by choosing only the longest read of each zero-mode waveguide (ZMW) and requiring subsequently a minimum read length of 4 kb. The resulting 6.7-11.4 million reads (52X - 70X coverage) for all 6 bats were stored in a DAZZLER database.

2.2.2 Read patching

The patch phase detects and corrects read artefacts including missed adapters, polymerase strand jumps, chimeric reads and long low-quality read segments that are the primary impediments to long contiguous assemblies. We first computed local alignments of all raw reads. Since local alignment computation is the most time- and storage-consuming part of the pipeline, we reduced

runtime and storage by masking repeats in the reads as follows. First, low complexity intervals, such as micro satellites or homopolymers, were masked with DBdust (all tools relate to the corresponding git repositories that are specified above). Second, tandem repeats were masked by using datander and TANmask. Third, as described in ref. [11], we split all reads into groups representing 1X read coverage. For each group, we then aligned all reads against all others with daligner and masked all local regions in each read where at least 10 other reads aligned. The repeat masks were subsequently used to prevent k-mer seeding in repetitive regions when computing all local alignments between all reads.

Repeat masking can sometimes be disadvantageous, especially in highly repetitive regions of the genome. Low quality or noisy regions occur randomly in PacBio reads. In case such bad regions are spread into repetitive regions, they induce premature alignment breaks and the repeat mask prohibits further computation of local alignments within the repeat. This can result in alignment piles, where the alignment patterns for chimeric reads, strand jumps and noisy regions cannot be detected anymore. In the worst case the repetitive region is trimmed back in all PacBio reads, which creates dead ends in the following assembly step.

To overcome this problem, we used LAseparate to find proper alignment chains that prematurely end in repeat regions. For those alignment chains, we recomputed local alignments with the repcomp tool without using the repeat mask. Then we applied LAfix, which we further improved in the ability to detect chimeric breaks within repeat regions. Usually, the detection of chimeric reads is based on the alignment pattern that is caused by the chimeric break point, *i.e.* the set of reads that are aligned to the left of the chimer break point is disjoint with the set of reads that are aligned to the right. Furthermore, a chimeric break induces a clear wall of alignment ends and starts at both sides. In repetitive regions, especially in microsatellites, this is not necessarily the case and an interleaved alignment pattern may occur, which complicates the detection of exact break points. To resolve those issues for the bat assemblies, repetitive regions up to a length of 8 kb were analysed for chimers. Any subread which included a repetitive region that could not be spanned by at least three valid alignment chains was marked as chimeric read. This method identified between 0.51% (*P. kuhlii*) and 1.96% (*P. discolor*) chimeric reads. Due to sufficient read coverage, all of them were discarded.

2.2.3 *De novo* assembly

In the assembly phase, we first calculated all overlaps between the patched reads using the same masking and alignment strategy of the patch phase. In addition, we applied an overlap chain rescue step. This step handles cases where a bad quality region was located at the tip of a subread, *i.e.* the interval from the tip to the minimum overlap length of the local alignment step (default 1.5 kb). In these cases, the bad quality region was not patched and therefore no proper overlaps were found. In order to avoid this behaviour, all alignment chains that prematurely ended due to a bad quality interval at subread tips were analysed with the Daccord tool forcealign. Forcealign tries to extend alignments by applying an increased error rate. For the bat assemblies this value was set to 35%. Only those alignments, which reached either a valid end in the A-read or in the B-read, were kept.

The subsequent steps were based on the generated overlaps and the original Marvel assembly pipeline[6,7]. First, the initial repeat annotation that only accounted for frequent repeats was updated by running LArepeat. Repeat regions were determined based on the coverage of the overlaps. If a potential repeat region had a coverage of more than twice the expected coverage of the genome, the region was annotated as a repeat. All following bases that had a coverage of at least 1.5 times the expected coverage were marked as repeat regions, in order to compensate for coverage fluctuations in repeat regions. The end of a repeat region was defined as the point where the coverage fell below 1.5 times the expected coverage. The expected coverage was calculated from the overlaps itself and was not given as an argument.

The minimum overlap length of 1.5 kb can result in missing repeat annotations if the ends of reads are repetitive, but do not reach far enough into a repeat. To avoid this problem, we used TKhomogenize to transitively transfer the existing repeat annotations between reads.

The remaining gaps shorter than 100 bp within the pairwise alignments were stitched with LAstitch. Quality scores for all reads were then recalculated and trim tracks were generated by LAq. Next, we used LAgap to rescan the reads for remaining gaps (points which were not spanned by any overlap). Gaps at this stage usually exist due to left-overs of the “weak” regions in the reads that are not detected in previous stages. In order to resolve a gap, the overlaps from the shorter side were discarded. Gap resolution was followed by a round of trimming with LAq.

Based on the remaining overlaps and the updated trim track a final overlap filtering was performed with LAfilter, which discarded local alignments and repeat induced overlaps. For the six bats, we required that proper overlaps were at least 4 kb long and had at least 1000 anchor bases.

Based on the final set of overlaps, an overlap graph was built using OGbuild. Touring the overlap graph was performed by OGtour. The look-ahead for finding all potential paths was set to 10. Afterwards the touring paths were used to create raw-sequence contigs with tour2fasta. To correct base errors of the raw sequence contigs, we used the Marvel correction module, which is also part of DAmar. In this step, only alignment piles from reads, which were used in the touring, were used to produce a consensus for the corrected contigs. This approach was very fast and reduced the error rate down to 1-2%.

The resulting corrected contigs were analysed and classified with CTanalyze, which separated the contigs into three different sets: primary, alternate and discarded. To this end, the contigs were aligned against each other and these alignments were used to derive a repeat mask. Further information, such as touring relation, patched-read mapping position, coverage, and repeat tracks, was integrated without realigning all reads against the assembly. The main task of CTanalyze is the haplotype separation into a primary contig set and an alternative contig set. For a reliable classification, different contig relations were combined into a multi relation matrix and a consensus classification was derived.

a) Graph touring relation: alternative contigs usually contain large structural variations that differ from the corresponding primary contigs. The graph touring also reports alternative contigs as bubbles or spurs.

b) Contig alignment relation: Contig overlap chains that allow for large structural variation are analysed for containment, bridging and forking relations.

c) Patched read intersection relation: If no reliable contig alignment chain could be found or the size of structural variation is larger than the alignment between two contigs, a) and b) may provide ambiguous or even no information. In that case, the original patched read overlap piles are analysed and if a major fraction of the PacBio reads is shared between two contigs then the smaller contig is assigned as a containment relation.

Afterwards putative primary contigs were further filtered and contigs that had an average coverage below 5, were more than 80% repetitive and were smaller than 20 kb were discarded. In addition to the contig classification, CTanalyze also reported potential issues, such as putative false joins, low coverage drops within contigs, and putative bridges between contigs. For the six bats, the potential issues (between 2-10 per species) were manually inspected and corrected if necessary.

2.2.4 Error polishing

The primary and alternate contigs were further polished by using the raw PacBio reads and applying two rounds of Arrow (https://github.com/PacificBiosciences/GenomicConsensus.git) polishing. Arrow decodes polished sequence in capitals, whereas unpolished sequence was represented in lower case bases. DAmar contigs tend to end within large repeats, which could not

always be fully polished. To facilitate the later scaffolding process, uncorrected contig ends that remained after the second polishing round were trimmed back.

To further correct base errors and reduce remaining length errors in homopolymer regions, 10x read clouds were used. To map 10x read clouds to the Arrow-polished contigs, the 10x Genomics Longranger align pipeline (https://github.com/10XGenomics/longranger, version 2.2.0) was applied, which uses the barcode-aware mapping tool Lariat. Afterwards the variant detector FreeBayes (version 1.2.0, default parameters + region argument to parallelise over number of contigs)[12] detected polymorphic positions and fixed erroneous non-polymorphic sites in the reference sequence using bcftools consensus (version 1.9) (https://github.com/samtools/bcftools). 10x read cloud polishing was iteratively applied in two rounds.

2.2.5 Haplotype phasing

So far, the assembly pipeline did not account for heterozygous events at the base level and the contigs did contain a mixture of both alleles. To address this problem, the 10x Genomics Longranger wgs pipeline with FreeBayes (version 1.2.0, default parameters + region argument to parallelise over number of contigs)[12] as the variant caller was used. Based on the phased VCF output file, bcftools consensus was used to produce locally-phased primary contigs. Depending on the 10x molecule lengths, the phased N50 of the bats ranged from 0.9 Mb (*P. discolor*) to 6 Mb (*M. molossus*).

2.2.6 Bionano scaffolding

2.2.6.1 *De novo* assembly

The Bionano raw molecules were assembled with Bionano Solve (Version 3.3) that offered command line tools for analysing Bionano data. An additional signal to noise filtering (filter_SNR_dynamic.pl) was required for two bat species (*R. ferrumequinum*, *P. discolor*) for which data from BSSSI and BSPQI nicking enzymes of the Saphyr system was available. The other four bat species, for which the newer DLE-1 direct labelling technique was used, did not require a SNR filtering step.

To assemble the optical maps of the six bats, we used all molecules ≥150 kb that additionally have at least 9 sites. The number of extension and search operations was set from the default 5 to 10, but after the 7th iteration most optical map assemblies converged, and no major changes were recognized. For each bat, we generated two maps using two different assembly option argument files: nonhaplotype_noES_saphyr.xml (noES) and nonhaplotype_saphyr.xml (ES). The noES option file resulted in more contiguous assemblies with higher N50 values. The nonhaplotype_saphyr.xml option file resulted in assemblies that were larger due to uncollapsed heterozygous maps. Both assembly versions were created for all six bats and evaluated in the following hybrid scaffolding step.

2.2.6.2 Hybrid scaffolding

The input to the Bionano hybrid scaffolding were the locally phased primary contigs, which were *in silico* digested by using the corresponding restriction sites (DLE-1: *M. myotis*, *P. kuhlii*, *R. aegyptiacus*, *M. molossus*; BSPQI and BSSSI: *R. ferrumequinum*, *P. discolor*) and the previously created Bionano assemblies. For *R. ferrumequinum* and *P. discolor*, the two-enzyme hybrid scaffolding procedure was performed using the wrapper script runTGH.R of Bionano Solve. The other four bat assemblies were scaffolded with hybridScaffold.pl, which is also part of the Bionano Solve command line tools. The conflict filter level for Bionano cmaps and contig cmaps were set to 2, *i.e.* if the genome map does not have long molecule support at the conflict junction, then the map is cut. Otherwise the sequence fragment is cut.

Scaffolds that were based on the noES-Bionano assembly had more contigs integrated and therefore had a higher scaffold N50 compared to scaffolds that were based on the ES-Bionano assembly. The correctness of the scaffolds was validated with Bionano Access and manual inspection of the raw molecule coverage that supported each contig integration. Furthermore, the Hi-C reads were mapped to the Bionano scaffolds, and HiGlass[13] was used to explore the genomic contact matrix. With the exception of *R. aegyptiacus*, the noES-Bionano assembly outperformed the ES version for the other five bats. For *R. aegyptiacus*, the noES-based scaffolds included a 110 Mb scaffold, which contained a 60 Mb gap. When inspecting the ES-based scaffolds the gap was filled with a 60 Mb contig, which could not be integrated when using the noES-Bionano assembly. This could be explained by the fact that *R. aegyptiacus* was the only bat for which we could not generate the Bionano data from the same individual. As the Hi-C data indicated that all other noES-Bionano scaffolds were valid, the missing 60 Mb contig was manually integrated into the gap location.

2.2.7 Hi-C scaffolding

To map the Hi-C Illumina read pairs to the previously created Bionano scaffolds the program bwa (version 0.7.17-r1194)[14] was used. The alignments were filtered according to the Arima filtering protocol (https://github.com/ArimaGenomics/mapping_pipeline). The resulting alignments were scaffolded with the Hi-C scaffolder Salsa2 (version 2.2)[15]. The clean option that detects misassemblies in the input assembly was enabled.

2.2.8 Manual curation

To visually inspect and validate the final scaffolds, we used the web-application HiGlass. To this end, Hi-C reads were mapped with bwa (version 0.7.17-r1194) to the Salsa2 scaffolds and the alignments were filtered und successively converted into multi-resolution cooler files.

Our inspection revealed that the overall scaffolding quality was already quite high (Extended Data Fig. 1c). However, visualization revealed a few false joins and unique off-diagonal interaction patterns that suggested joining scaffolds. Scaffolds were split if the Hi-C read mapping density around the diagonal was not supported (Extended Data Fig. 1c – highlighted with ellipse 1). Scaffolds were joined if the read mapping density in the off-diagonal was increased and the map resolution allowed a unique placement (Extended Data Fig. 1c - highlighted with ellipse 2). For each bat, up to 10 splits and 10 joins (*P. discolor*) were manually performed and the curated scaffolds were validated again by HiGlass (version 0.6.3) (Extended Data Fig. 1c).

For *P. kuhlii*, we initially did not manually curate the assembly using Hi-C data and this initial assembly (referred to as 'non-curated' below) was used for annotation and all analyses in this manuscript. During the revision, we performed manual curation for *P. kuhlii* as well, which resulted in 97.99% of the assembly being assigned to chromosome-level scaffolds, as detailed below. This curated assembly is also provided on NCBI and GenomeArk.

2.3 Assembly results

After applying the DAmar assembler to the PacBio reads, two rounds of Arrow and FreeBayes polishing, and haplotype phasing in combination with the 10x read clouds, the output of this process (described above) is a collection of contigs that are either considered primary, alternate, or contigs that we discarded due to their small size. For all 6 bats, we obtained assemblies comprising just several hundred primary contigs. To determine N50 values, we used the Perl script assemblathon_stats.pl that is part of the Assemblathon 2 analysis pipeline[16] (https://github.com/ucdavis-bioinformatics/assemblathon2-analysis), defining assembly gaps as runs of ≥10 N's. The N50 of the contigs was >10 Mb in every case and correlated with the average read length of the data set. The number of contigs also roughly inversely correlated with read length adjusting for overall genome size (*e.g. M. molossus* had the highest average read length but 396

contigs versus only 260 for *R. aegyptiacus* because the latter has a 1.9 Gb genome whereas *M. molossus* has a 2.3 Gb genome). Supplementary Table 29 gives the number of contigs, total base pairs in these contigs, and N50 length for each species and each contig class. The number of alternate contigs varied significantly, presumably reflecting the level of structural heterogeneity between the haplotypes of the individual's genome.

The results of scaffolding the locally-phased primary contigs with the assembled Bionano optical maps are shown in Supplementary Table 30. In all cases, we obtained very large scaffolds just using these optical maps. The assemblies of *M. myotis* and *P. kuhlii* were more fragmented, which reflects the less contiguous assembled maps for these two genomes. The scaffolding process can occasionally fuse contigs that are implied to actually overlap, and break contigs that clearly disagree with the optical maps. Supplementary Table 30 also shows that very few such contig breaks were introduced by Bionano scaffolding.

After Bionano scaffolding, we generated final scaffolds using the Hi-C data. This scaffolding step again joined and broke scaffolds, but scaffold breaks typically occurred only at the tips, as shown by the very small "delta" values for contig NG50 in Supplementary Table 31. For all six bats, Hi-C scaffolding substantially increased scaffold N50 sizes by 25-100% and in particular spanned centromeres brinding chromosome arms together in the same scaffold. We found that the bats for which we generated only 15X of Hi-C data (*M. myotis*, *P. kuhlii*, and *R. aegyptiacus*) had slightly smaller scaffold N50 values than the bats for which 60X was generated (Supplementary Table 31). This suggests that a higher coverage of Hi-C read pairs is desirable for scaffolding and we aim at generating 60X or more in future projects.

In a final step, Hi-C maps such as illustrated in Extended Data Fig. 1c, are used to manually split and join scaffolds. The results of this manual curation are shown in Supplementary Table 3. It is noteworthy that manual curation detected very few conflicts leading to scaffold breaks but rather mostly involved joining scaffolds.

Unfortunately, the manual curation of *P. kuhlii* only occurred after the final, very time and compute expensive annotation and analysis of the pre-curation assembly had been performed. Therefore, the annotated genomes in the various public repositories do not reflect the curated result, but the result prior to curation. In what follows we speak only to the statistics of the curated assembly, but in Supplementary Tables 2-3 we give a row for the statistics of the assembly prior to curation. Since this curation does not affect contigs, the annotation and analysis are unaffected other than coordinate locations.

To assess whether our scaffolds often represent chromosomes, we used available karyotypes for each species to estimate the length of each chromosome (https://git.mpi-cbg.de/dibrov/chromosome_size). It should be noted that the length of small chromosomes (less than 20 Mb) is hard to estimate as these chromosomes are but a small blob in the karyotype images. We plotted the estimated lengths of the chromosomes against the length of our final scaffolds. The karyotype estimate was always larger than the next largest scaffold, presumably because the scaffolds did not have accurate gap lengths for the large centromere between chromosome arms. Nevertheless, as shown in Extended Data Fig. 2a for three of the bats, we found good agreement between estimated chromosome lengths and the lengths of our scaffolds. Specifically, the correlation coefficient between the N karyotypes of a species, and the N largest scaffolds is given in Supplementary Table 3 and was always above 0.989. We further examined the remaining scaffolds not correlated with the karyotype and characterized this residual into three informal categories: Cliff(x) = no scaffolds over 2 Mb remain and x karyotypes have no corresponding scaffold, Incline(x) = x scaffolds over 2 Mb remain but all were significantly smaller (35% or less) than the smallest karyotype, Tail(x) = x scaffolds over 2 Mb remain and the size distribution gradually declined from the last scaffold assigned to a karyotype. We found that, with the exception of *M. myotis* and *P. kuhlii*, all other assemblies had Cliff or Incline endings, suggesting that almost all the scaffolds corresponded to chromosomes. In

summary, for all six bats, more than 95.6% of the assembly was in the largest N chromosomes (Supplementary Table 3).

Supplementary Table 2 shows the final assembly statistics for our locally-phased primary contigs and our scaffolds of these. QV metrics were computed by mapping all the 10x read data to the final contigs and analysing discrepancies with the Illumina reads. This showed that our assemblies achieved the desired QV40 metric.

In summary, for all six bats, our sequencing and assembly strategy produced assemblies with contig N50 values ranging from 10.6 to 22.2 Mb (Fig. 1b and Supplementary Table 2). Thus, our contigs are much more contiguous than previous short read-based assemblies of bats (Extended Data Fig. 1d). Our scaffold N50 values ranged from 92 to 171.1 Mb (excluding *P. kuhlii* pre-curation) and were often limited by the size of chromosomes (Fig. 1b and Supplementary Table 2). We estimated that 95.6 to 99% of each assembly is in chromosome-level scaffolds (Supplementary Table 3, excluding uncurated *P. kuhlii*). Applying BUSCO to the genome assemblies, we found that between 92.9 and 95.8% of BUSCO genes were completely present in our assemblies, which is comparable to the assemblies of human, mouse, and other Laurasiatheria (Extended Data Fig. 3a and Supplementary Table 4). Consensus base accuracies across the entire assembly range from QV 40.8 to 46.2 (Supplementary Table 2) for the six bats (where QV 40 represents 1 error in 10,000 bp). Since the algorithms for assembling, scaffolding, and haplotyping are an active area of research[17], we expect that in the future even more complete genome reconstructions can be produced with the data we collected. Importantly, our genomes meet the Vertebrate Genome Project[18] (VGP: https://vertebrategeomesproject.org/technology) minimum standard of 3.4.2QV40 (defined as a contig N50 of 1 Mb or greater, a scaffold N50 of 10 Mb or greater, at least 90% of the assembly is assigned to chromosome-level scaffolds, and a consensus accuracy of Q40 or better, see https://vertebrategenomesproject.org/technology) and approach in fact 4.5.2.QV40. All assemblies have been added to the VGP collection, same for the curated version of *P. kuhlii*.

3. Genome annotation

3.1 Protein-coding gene annotation

3.1.1 Overview

To annotate coding genes, we used a variety of approaches and data to obtain evidence of coding genes in the bat genomes. These evidence comprise (i) projecting genes annotated in another mammal to our bat genomes via whole genome alignments, (ii) aligning protein and cDNA sequences of related mammals, (iii) mapping RNA-seq and Iso-seq data obtained for the six bats, and (iv) *de novo* gene predictions using a bat-specific gene model. These evidence were integrated into a consensus gene set, which was further enriched for high-quality isoforms. All individual evidence and the final gene set can be visualized and obtained from the genome browser. Below, we detail how each of the evidence was obtained and how they were integrated.

3.1.2 TOGA projections

As the first evidence, we projected annotations of coding genes from multiple reference genomes to our bat genomes using TOGA (Tool to infer Orthologs from Genome Alignments, last commit: 02/05/2019). Briefly, TOGA takes as input pairwise genome alignment chains between a designated reference and query genome[19], coding transcript annotations for the reference species and a file linking gene and transcripts isoforms. For each gene, TOGA identifies the chain(s) that aligns the putative ortholog in the query using synteny and the amount of aligning exonic and intronic sequence. To obtain the locations of coding exons of this gene, TOGA extracts the genomic region

corresponding to the gene on this chain from the query assembly and uses CESAR 2.0 (Coding Exon Structure Aware Realigner)[20] in multi-exon mode.

We applied TOGA to the genome alignments (see above) to project the Ensembl (version 96, last accessed: 26/04/2016) gene annotation for human (hg38) and mouse (mm10) to our six bats. Furthermore, the *M. lucifugus* (myoLuc2 assembly) Ensembl (v96) annotation was projected to our *M. myotis* assembly and the final gene annotation of *M. myotis* produced for this project was projected to the other 5 bat species. The number of projected genes for each of the six bats is listed in Supplementary Table 32.

3.1.3 Alignments of protein and cDNA sequences of related bat species

As the second evidence for coding genes, we aligned protein and cDNA sequences of related species to our six bat assemblies. For each of the six bats, we downloaded protein and RNA transcript sequences from NCBI or Ensembl for one other close-related bat species that has annotated genes (Supplementary Table 33). Protein and transcript sequences were filtered to retain only those with matching peptide and mRNA sequence. Then, we used GenomeThreader (v1.7.0)[21] to simultaneously align protein and mRNA sequences to the respective target genome. GenomeThreader was run using the Bayesian Splice Site Model (BSSM) trained for human and default parameters aside from those detailed below. For protein alignments, we used a seed and minimum match length of 20 amino acids (prseedlength 20, prminmatchlen 20) and allowed a Hamming distance of 2 (prhdist 2). For the transcript alignments, we used a seed length and minimum match length of 32 nucleotides (seedlength 32, minmatchlen 32). At least 80% of the protein or mRNA sequence was required to be covered by the alignment (-gcmincoverage 80), and potential paralogous genes were also computed (-paralogs). For *M. molossus*, these stringent parameters produced much fewer gene predictions compared to the other five bats, likely due to the increased phylogenetic distance between *Molossus* and *Miniopterus* (from which we used annotated genes) compared to the other species pairings. Therefore, we performed an additional GenomeThreader run for *M. molossus* using less stringent parameters (default parameters for the seed, minimum match lengths and Hamming distance). The stringent alignments were provided as hints to Augustus (below), while the less stringent gene predictions were used for consensus gene prediction. For the other 5 genomes, the stringent alignments provided hints for Augustus and were used for consensus gene prediction. The number of filtered gene alignments for each of the six bats is listed in Supplementary Table 32.

3.1.4 Transcriptome data

As a third evidence for genes, we used RNA-seq and Iso-seq transcriptomic data that were mostly newly-generated for each of the six bats in this project. Supplementary Table 34 provides details of the tissues used to generate transcriptome data and lists Sequence Read Archive (SRA) accession numbers used to download previously generated data.

For RNA-seq, reads were stringently mapped to the respective genome using HISAT2 (v2.0.0)[22], removing reads with greater than 5% ambiguous characters (-n-ceil L,0,0.05), disallowing discordant and mixed alignments (--no-discordant --no-mixed), and using the --dta (downstream transcriptome assembly) flag. The resulting SAM file was sorted and converted to BAM format using Samtools (v1.9)[23]. Transcripts were assembled using StringTie (v1.3.4d)[24] with default settings.

Since RNA-seq data also contains non-coding transcripts, we next filtered for transcripts that contain an open reading frame (ORF) and are not potential nonsense-mediated decay (NMD) targets. To this end, we used the Transcriptome Annotation by Modular Algorithms (TAMA) package (https://github.com/GenomeRIK/tama.git; accessed 21/5/2019; commit 58f9d98), which predicts ORFs for all assembled transcripts. Putative peptide sequences were queried against the Swissprot database (downloaded 20/05/2019) using blastp from the BLAST+ suite (v2.6.0) with default parameters[25]. BLAST results were parsed, designating a coding sequence (CDS) and mapping this to the corresponding exon structure of each transcript. Transcripts identified as full length by TAMA

were retained and used as input for consensus gene models. The number of transcripts obtained from RNA-seq for gene annotation is reported in Supplementary Table 32.

We used our Iso-seq data to produce high quality ORF predictions. To this end, raw reads were first processed using the IsoSeq3 pipeline (version 3.1.0) (https://github.com/PacificBiosciences/IsoSeq3) with the arrow polish flag on. The resulting high-quality transcripts (HQ) (full-length and supported by more than one read) and FLNC reads (full-length non-chimeric reads before the clustering step) were further processed in parallel. The FLNC and HQ PacBio BAM files were converted into FASTA format using Bamtools (version 2.4.1) and aligned to the reference genome with Minimap2 (-t 16 -ax splice -uf --secondary=no -C5, version 2.10-r784-dirty). The resulting BAM files were filtered to retain only primary alignments using Samtools (version 1.9). TAMA collapse (https://github.com/GenomeRIK/tama.git) was applied to both HQ and FLNC primary alignments to predict non-redundant transcript sets.

The resulting transcript coordinates were used to extract corresponding genomic sequences with Bedtools (getfasta –split –name -s, version v2.27.1) for both the HQ and FLNC set. ORF prediction was run in two steps. First, the TAMA-GO package was run on HQ transcript sequences (see above) resulting in the annotation of a putative ORF in each transcript. The putative CDS coordinates were used to determine and filter out potential targets of nonsense-mediated decay[26] by removing all transcripts that have more than one intron in the 3'UTR or transcripts in which an intron is located more than 50 bp downstream from the stop codon. The resulting set (HQ.nonnmd) was used to train an ANGEL ORF prediction model (https://github.com/PacificBiosciences/ANGEL). The FLNC.nonnmd set was produced using TAMA-GO in the same way as described above and was used as input for ANGEL in prediction mode (output_mode=best --min_angel_aa_length 100 --min_dumb_aa_length 100) with the model trained in the previous step. The resulting annotations were used to split the FLNC.nonmd transcript set into three groups: (i) ANGEL positive (with evidence of an ORF predicted by ANGEL); (ii) ANGEL negative but with blastp hits; (iii) ANGEL negative and no blastp hits. ANGEL positive, full length transcripts were provided as gene predictions for consensus gene prediction. The number of putatively coding transcripts obtained with Iso-seq for each of the six bats is listed in Supplementary Table 32.

3.1.5 *De novo* gene prediction:

As a fourth piece of evidence, we generated *de novo* gene predictions using Augustus (v3.3.1)[27]. To this end, we first trained a bat-specific Augustus model using *M. myotis* as a representative species and the BRAKER pipeline (v2.1)[28]. BRAKER uses extrinsic evidence (RNA sequencing and/or proteins from a close-related species) as training data and performs iterative gene prediction to train model parameters. We used an earlier contig assembly of *M. myotis* and provided GenomeThreader alignments of *M. lucifugus* proteins (downloaded from Ensembl, date: 8/8/2018) and a BAM file of mapped *M. myotis* RNA-seq data from several tissues (kidney, liver, heart and brain) as input to BRAKER. The resulting "bat" model was used in subsequent Augustus runs.

Augustus is able to use extrinsic evidence as hints when predicting genes in a newly-sequenced genome. We compiled the following data as Augustus hints. RNA-seq was used to produce intron hints using the Augustus bam2hints module with the introns-only flag. RNA-seq derived hints was given a 'priority' of 4. High quality ORFs predicted from Iso-seq transcripts and classified as positive using ANGEL (described above) were converted to BAM format, and bam2hints was used to produce intron, exon and exonpart hints. Iso-seq derived hints were given a priority of 6. GenomeThreader alignments were converted to hints using the align2hints.pl script provided in the BRAKER distribution. This produced CDSpart, intron, start and stop hints that were given a priority of 4. Identical hints were merged using the join_mult_hints.pl script from Augustus. Further, human (Gencode version 27) and mouse (Gencode version 16) gene annotations were provided as high weight CDS and intron "manual" hints when running Augustus in comparative mode.

Augustus was run in two modes, in single genome mode for each of our six assemblies and once in comparative mode using a multiple genome alignment. For single genome mode, human TOGA projections were used to divide each genome into approximately 2.5 Mb regions with 250 kb overlap, avoiding splits inside putative genes. Augustus was run with the trained "bat" model and a custom extrinsic config file containing the bonus and malus parameter for each hint type. Alternative splice forms were predicted from evidence (alternatives-from-evidence), and AT/AC splice sites were allowed if supported by hints (allow_hinted_splicesites=atac). The resulting GTF files of gene predictions for each region were merged using the Augustus joingenes module.

For Augustus in comparative mode, we used the multiple genome alignment (MAF format) produced by MultiZ (v11.2) with *M. myotis* as the reference species as input. We used the split regions determined for single genome mode for *M. myotis* to split the MAF file into non-overlapping 2.5 Mb regions. A database containing the genomes for the 6 bat species, human and mouse, and the hint data was constructed, and a custom extrinsic config file was provided. All genomes were provided as soft-masked (repetitive sequence indicated as lower case letters). The phylogeny with branch lengths as estimated using IQ-Tree (see Supplementary Note 4.2 "Phylogenetic inference and divergence time estimation" below) was trimmed to contain only the species in the MAF file, and also provided to Augustus in Newick format. GTF files of gene predictions for each species were merged using the Augustus joingenes module. The number of predicted transcripts for each of the six bats is listed in Supplementary Table 32.

3.1.6 Integrating all gene evidence into a final gene annotation

We used EVidenceModeler (v1.1.1)[29] to integrate the gene evidences from TOGA projections, Genome Threader alignments, Augustus gene models and transcript ORF predictions from Iso-seq and assembled RNA-seq reads into a consensus gene set. Augustus gene predictions from the single and comparative mode were designated as *ab initio* predictions and given weights 2 and 1 respectively. GenomeThreader alignments were designated protein alignments with weight 2. The TOGA projections were given as "other" predictions all with weight 8. Transcript ORF predictions from assembled RNA-seq were filtered for those labelled as full length by TAMA and were provided as "other" predictions with a weight of 10. ANGEL positive ORF predictions from Iso-seq data that were also labelled as full length by TAMA were provided as "other" predictions with a weight of 12. Genomes were partitioned using EVidenceModeler into 1 Mb chunks with 150 kb overlap. Consensus gene models were called for each partition. EVidenceModeler output was converted into GTF format using in-house Perl scripts. We used the joingenes function from Augustus to combine all outputs into a consensus gene set.

EVidenceModeler does not, by default, produce consensus gene models for genes that are nested in an intron of another gene. Although this behaviour can be enabled via a parameter, it also produces a high number of likely false positive gene models. Therefore, in order to rescue these intronic genes, we incorporated TOGA projections from human and mouse with no CDS overlap to any already-detected consensus gene model. TOGA projections were only considered for incorporation if the gene began and ended with canonical start and stop codons and contained no internal stop codons. Further, only APPRIS "Principal" isoforms[30] were considered. Transcripts were added first from human and then from mouse.

As we used the *M. myotis* gene annotation as input for TOGA projections to the other five bats, we visualised the gene annotation in a genome browser and screened for obvious annotation errors such as potential genes lacking a consensus model, fused or split genes. Manual refinement and correction of a few loci was performed where necessary.

EVidenceModeler produces a single consensus gene model for each locus, and therefore will not annotate exons or splice sites that only occur in alternative isoforms of the same gene. We therefore used evidence sources of high confidence to incorporate isoforms to already-detected gene loci if an isoform provided novel splice information relative to the annotated consensus isoform. We

did not incorporate isoforms that are potential NMD targets, defined as transcripts having more than two introns in the 3'UTR or transcripts in which an intron is more than 50 bp downstream of the stop codon[26]. Isoforms predicted from Iso-seq data were added as priority, followed by RNA-seq derived transcripts and finally TOGA projections. RNA-seq transcripts were filtered to remove those which may represent 5' degraded transcripts, identified as a transcript with no novel splice sites and a start codon nested within a previously annotated exon or having more than two 5' non-coding exons. A TOGA gene projection was only considered if it was an APPRIS Principal isoform, had canonical start and stop codons, no internal stop codons and that all coding exons from the reference were projected.

3.1.7 Prediction of 3'UTR sequences from Iso-seq transcripts

3'UTR sequences were predicted using FLNC.nonmd Iso-seq transcripts set as follows. First-pass 3'UTR coordinates were created using CDS predictions, from the stop codon to the end of the transcript. Then, a custom script was run to cluster all 3'UTR coordinates per gene locus that shared the stop codon coordinate but varied in the 3' most (end of 3'UTR) coordinate. For these cases, we chose the longest 3'UTR per cluster and assigned it a weight, defined as the number of Iso-seq transcripts that shared this stop codon coordinate. Next, if more than one clustered 3'UTR per gene locus was found, the one with the highest weight was selected. Finally, the set of the candidate 3'UTRs was compared to gene annotations of our bats and only the sequences with a stop codon within a 100 bp window from the end of the annotated CDS of a gene were retained.

3.1.8 Filtering transcripts for coding potential and assigning gene symbols

Manual inspection showed that integrating transcripts from a variety of evidence also included a number of genes that are unlikely to code for a protein and may represent non-coding or erroneous genes. In particular, many Iso-seq transcripts only had short and non-conserved predicted ORFs, indicative of non-coding genes, but were included by EVidenceModeler because of the high weight we gave this high-confidence transcript evidence. To remove putative erroneous or non-coding genes, all putative peptide sequences were queried against the Swissprot database using blastp with a minimum E-value of $1e^{-10}$. Sequences with no match to a mammalian sequence in the database were removed if they were smaller than 120 amino acids. Reported hits were further filtered, only retaining a match which covered >75% of the query sequence and >50% of the subject, and >50% positive scoring matches.

We assigned the human gene symbol to an annotated gene in bats if the CDS overlapped between the locus and a single TOGA-projected human gene. Genes for which we could not assign a gene symbol based on TOGA projections were assigned a symbol based on the previously computed BLAST alignments. BLAST alignments were divided into complete matches (>65% query coverage, >70% subject coverage, and >30% identity), and partial hits (>75% query coverage, >50% subject coverage, and >50% positive scoring matches). Gene symbols were retrieved for all matches with a bit-score no less than 85% the value of the top hit. Gene symbols from the majority of retained hits were assigned to a gene. Genes with no complete matches were assigned a symbol from the partial matches, with an appended 'L' to indicate the partial match. When multiple loci were assigned the same symbol, they were distinguished by incrementing a trailing alphanumeric character.

3.1.9 Computing Annotation Completeness

In order to assess the completeness of the protein coding annotation, we used BUSCO (version 3)[31] with the mammalian (odb9) protein set. Predicted peptide sequences from the six bat species along with annotated peptide sequences for seven other mammal species, including human (hg38), mouse (mm10), pig (susScr11), cow (bosTau8), cat (felCat8), horse (equCab3) and dog (canFam3), were downloaded from Ensembl (version 96) (Supplementary Table 1). BUSCO was run in protein mode, and the number of complete, fragmented and missing genes were compared across assemblies.

For the six bats, we annotated between 19,122 and 21,303 coding genes (Fig. 1e). These annotations completely contain between 99.3 and 99.7% of the 4,104 highly conserved mammalian BUSCO genes (Fig. 1d and Supplementary Table 4), showing that our six bat assemblies are highly complete in coding sequences. Since every annotated gene is by definition present in the assembly, one would expect that BUSCO applied to the protein sequences of annotated genes and BUSCO applied to the genome assembly should yield highly similar statistics. However, the latter finds only 92.9 to 95.8% of the exact same gene set as completely present, showing that BUSCO applied to an assembly only, underestimates the number of completely contained genes (Extended Data Fig. 3a).

3.2 Analysis of ultraconserved elements

To assess the completeness of non-exonic regions in mammalian assemblies, we determined the number of aligning ultraconserved elements per assembly. Since the 481 ultraconserved elements (UCEs) were originally defined as genomic regions ≥200 bp that are identical between human, mouse and rat[32], we did not use the human and mouse genomes in this comparison as by definition all UCEs are present in these assemblies. As in a previous study[6], we focused on the 197 UCEs that do not overlap exons according to the human Ensembl gene annotation and that align to chicken (galGal5 assembly) and teleost fish (zebrafish danRer10, medaka oryLat2). Given their strong conservation across vertebrates, we expect that these 197 vertebrate non-exonic UCEs are present in mammalian genomes.

To align these 197 ultraconserved sequences against mammalian genomes, we used Blat (v36x2)[33] with sensitive parameters (-minIdentity=60 -minScore=30 -minMatch=1 -stepSize=8 -mask=lower). We kept those Blat hits where the alignment had a minimum identity of 85% and at least 150 of the ≥200 bp in the ultraconserved sequence aligned. This number of aligning UCEs is shown in Extended Data Fig. 2b.

As expected, the vast majority of UCEs were detected in all assemblies. To investigate why 15 UCEs did not align with these criteria to individual assemblies, we inspected these UCEs in the human UCSC genome browser in the context of a multiple genome alignment of mammals and pairwise alignment chains. Supplementary Table 5 lists the details of all these 15 UCEs. We used the nearest up- and downstream aligning block in the chain to determine whether the UCE maps completely or partially to a query genomic locus that includes an assembly gap, as shown in Extended Data Fig. 2c. These UCEs were classified as ‘missing due to assembly gap’. This applied to two to four UCEs that were not detected in *Miniopterus*, dog, cat, and cow (Supplementary Table 5). Consistent with assembly gaps being the underlying issue, an analysis of the newer assemblies of cow (bosTau9) and cat (felCat9) that recently became available showed that all UCEs that were missing in their previous bosTau8 and felCat8 assemblies are now entirely present in the newer assemblies (Extended Data Fig. 2c).

For *M. myotis* and *P. kuhlii*, one and three UCEs could not be detected when using an 85% identity threshold. However, these UCEs are not missing due to assembly incompleteness. Instead, these UCEs are present in our *Myotis* and *Pipistrellus* assemblies but were not detected because they exhibited substitutions and smaller insertions/deletions that decreased the alignment identity below our 85% threshold. For these UCEs, we used BLAST with default parameters for the 10x Genomics Illumina reads and daligner with “-A -k11 -w5 -h35 -e.7 -l100 -M64” parameters for PacBio reads to confirm that (i) the genomic sequence aligning to the UCE is supported by both PacBio and by Illumina reads of the respective bat species and (ii) that the human ultraconserved sequence does not have a better match in any of the read data acquired for the respective bat. To further corroborate real sequence divergence in an otherwise ultraconserved element, we aligned the sequences of close-related bats with sequenced genomes and found that most mutations were shared among other independently-sequenced bats. These three diverged UCEs are shown in Supplementary Figures 1-3 and Extended Data Fig. 2d. Given the high degree of UCE sequence identity between mammals in general[34], these UCEs with true sequence divergence in certain bat lineages represent striking

exceptions. In summary, this analysis shows that our six bat assemblies are highly complete in non-coding regions.

3.3 Repetitive element annotation

We annotated each genome for transposable elements following the methods described in [35]. Briefly, each assembly was mined for potential novel TEs using RepeatModeler[36]. The resulting putative TE libraries were masked with RepeatMasker (v4.0.9)[36] and the results then processed using calcDivergenceFromAlign.pl in the RepeatMasker package to generate Kimura-2-parameter (K2P) distances. We presumed that younger TE families, defined as consensus sequences having hits with K2P distances less than 6.6% (approximating ~30 Myrs or less since insertion, based on a general mammalian neutral mutation rate of $2.2x10^{-9}$)[37], were lineage-specific and potentially undescribed. Consensus sequences were also filtered for size (>100 bp), subjected to iterative homology-based searches against the genome, and manually curated[35]. For each iteration, new consensus sequences were generated to match the top 50 BLAST hits. Bioinformatically, this was accomplished by aligning with MUSCLE (v3.8.31)[38], trimming the alignments with trimal (-gt 0.6 -cons 60) (v1.3)[39], and estimating a consensus with the EMBOSS script ‘cons’ (-plurality 3 -identity 3)[40]. Files with fewer than 10 BLAST hits were discarded. Curation of the estimated consensus by eye ensured accuracy by preventing inclusion of single indels and observing 5’ and 3’ TE ends to confirm the full length of each element in each alignment.

To confirm TE type, each TE was compared to three online databases: BLASTx to confirm the presence of known ORFs in autonomous elements, RepBase (v20181026) to identify known elements, and TEclass[41] to predict the TE type. We also used structural criteria as follows. For DNA transposons, only elements with visible terminal inverted repeats were retained. For rolling circle transposons, we required elements to have an identifiable ACTAG at one end. Putative novel SINEs were inspected for a repetitive tail and A and B boxes. LTR retrotransposons were required to have recognizable hallmarks such as TG, TGT or TGTT at their 5’ and the inverse at the 3’ ends. Finally, duplicates were removed via the program cd-hit-est (v4.6.6)[42,43] if they did not pass the 80-80-80 rule as described in [44].

The complete TE library for each bat was combined with a vertebrate library of known TEs in RepBase (v20181026). This library is available as Supplementary Data File 1. RepeatMasker was used to mask the genomes with this custom library. Postprocessing of output was performed using a custom script, RM2Bed.py (https://github.com/davidaray/bioinfo_tools), which eliminated overlapping hits and converted to Bed format. The same methods were used to analyse seven mammalian outgroups (Supplementary Table 1). The resulting data is shown in Extended Data Fig. 3b-c and Supplementary Table 35.

3.3.1 TE Results

Our assemblies revealed noticeable genome size differences within bats, with assembly sizes ranging from 1.78 Gb for *P. kuhlii* to 2.32 Gb for *M. molossus* (Extended Data Fig. 3b). As genome size is often correlated with transposable element (TE) content and activity, we compared TE content of the genomes of the six bats and seven other representative Boreoeutherian mammals (Laurasiatheria + Euarchontoglires), selected for the highest genome contiguity. This showed that TE content generally correlates with genome size (Extended Data Fig. 3b). Next, we compared TE copies to their consensus sequence to obtain a relative age from each TE family. This revealed an extremely variable repertoire of TE families with evidence of recent accumulation. For example, while the 1.89 Gb *R. aegyptiacus* genome exhibits few recent TE accumulations (~0.38%), while ~4.2% of the similarly sized 1.78 Gb *P. kuhlii* genome is derived from recent TE insertions (Extended Data Fig. 3c). The types of TE that underwent recent expansions also differ substantially in bats compared to other mammals, particularly with regard to the evidence of recent accumulation by rolling-circle and DNA transposons in the vespertilionid bats (Extended Data Fig. 3c). These two TE classes have been largely dormant in most mammals for the past ~40 million years and recent insertions are essentially

absent from other Boreoeutherian genomes[45]. These results add to previous findings revealing a substantial diversity in TE content within bats, with some species exhibiting recent and ongoing accumulation from TE classes that are extinct in most other mammals while other species show negligible evidence of TE activity[46].

3.4 Annotation and analysis of endogenous viral elements (EVE) and endogenous retrovirus (ERV)

3.4.1 EVE annotation and analysis

We analysed the bat genomes and seven additional mammalian genomes as outgroups (Supplementary Table 1) for the presence of endogenous viral elements (EVEs). Mammalian genomes were converted to nucleotide BLAST databases[47]. A comprehensive library of viral proteins (Supplementary Table 36) was queried against the mammalian genomes using tBLASTn (maximum E-value 0.001; maximum number of 100 alignments reported). The viral proteins span the viral classes and families listed in [48] and were updated to the current versions of the reference sequences for each virus. The results were manually inspected and total viral insertions under 100 amino acids in length were discarded. Reciprocal BLAST searches were run for each hit, with the best hit viral family considered the true identity. BLAST hits in regions annotated as functional mammalian genes were considered false hits. Nucleotide sequences for each identified viral family, plus extant representatives of the family and previously identified EVEs, were aligned with Aliview[49].

3.4.2 ERV annotation and analysis

All 6 bat genomes and the 7 additional mammalian genomes were searched with local BLAST[47] using 14 probes of the viral proteins Gag, Pol and Env from each genus of Retroviridae: alpha-, beta-, delta-, epsilon-, gamma-, lenti-, and spumaretroviruses (Supplementary Table 37). Using the custom Python (version 3.6+) script ERVin (https://github.com/strongles/ervin), we extracted all BLAST hits with an E-value ≤0.009 that comprised a length ≥400 amino acids for Pol regions and ≥200 amino acids for both Gag and Env regions. We grouped sequences according to their taxonomic relation to the first returned hit given by reciprocal BLAST. For the Pol region, we extracted the highly conserved 200 amino acid region ending with a ‘Y[M/V]DD’ motif for all the bats and the 7 other mammals, and aligned them using MUSCLE within the Aliview software (v1.25)[49]. We manually inspected sequences and corrected the alignment. We discarded all sequences where the highly conserved region was shorter than 50 amino acids.

To reconstruct the phylogenetic tree of the retroviral Pol-like sequences for all 6 bat genomes and the viral probes used in BLAST search, we first ran Prottest (v3.4.2)[50] to determine which model to use with RAxML (version 8) package[51]. The best model was the VT+G model according to the AICc scoring criteria.

3.4.3 EVE and ERV results

Our analysis showed that *M. molossus* displayed more gamma-like sequences for all 3 viral proteins in comparison to other bat species. Apart from that, we detected integrations for the following retroviral families: delta (*M. molossus*), epsilon (*P. kuhlii*), and spuma (*M. molossus* and *R. ferrumequinum*) for the Pol region; lenti and epsilon for Gag region (both in *P. kuhlii*); and alpha for the Env region (*P. discolor, R. ferrumequinum, R. aegyptiacus*) (Extended Data Fig. 8b). Overall, the highest number of integrations was observed in *M. myotis* (with the exception of the Env region which was observed most frequently in the *M. molossus* genome), while the greatest variety of genera was observed in *P. kuhlii*. We also compared the numbers of Pol, Env, and Gag regions found in bats to the 7 mammalian reference genomes (Supplementary Figure 6). Of all analysed genomes, *M. musculus* displayed the highest number of integrations of viral protein sequences. The numbers of Pol sequences found in the non-bat mammalian genomes and 5 of our 6 bat genomes were comparable to

each other, with *M. myotis*, whose genome contained twice more Pol and Gag integrations, being an exception. Apart from mouse, all of analysed bats exhibited more Env and Gag integrations in comparison to the other mammalian genomes.

We identified three predominant non-retroviral EVE families: *Parvoviridae*, *Adenoviridae* and *Bornaviridae* (Extended Data Fig. 8a). Parvovirus and bornavirus integrations were found in all bats except for *Rousettus* and *Molossus* respectively. A partial filovirus EVE was found to be present in the Vespertilionidae (*Pipistrellus* and *Myotis*), but absent in the other bat species, suggesting that vespertilionid bats have been exposed in the past to and can survive filoviral infections, corroborating a previous study[52]. Consistent with other mammals, the highest number of ERV integrations came from beta- and gamma-like retroviruses[53,54], with beta-like integrations most common for Pol and Gag proteins and gamma-like integrations most common for env proteins in most of the bats (Extended Data Fig. 8b and Supplementary Figure 6). Overall, the highest number of integrations was observed in *Myotis* (n=630), followed by *Rousettus* (n=334) with *Phyllostomus* containing the lowest (n=126; Extended Data Fig. 8b and Supplementary Table 38). Additionally, we detected ERV sequences with hits for alpha- and lenti-retroviruses in reciprocal BLAST searches. Until now, alpharetroviruses were considered as exclusively endogenous avian viruses[55]. Thus, our discovery of endogenous alpharetroviral-like elements in bats is the first record of these sequences in mammalian genomes, widening the known biodiversity of potential hosts for retrovirus transmission. We detected several alpha-like Env regions in *Phyllostomus*, *Rhinolophus*, and *Rousettus* (Extended Data Fig. 8b), showing that multiple and diverse bat species have been and possibly are being infected by alpharetroviruses. We also detected lentivirus gag-like fragments in *Pipistrellus*, which are rarely observed in endogenized form[56].

To identify historical ancestral transmission events, we reconstructed a phylogenetic tree from our recovered ERVs with the known viral protein ‘probe’ sequences for all six bat genomes and seven mammalian outgroups (Supplementary Figure 7). The majority of sequences group as single bat-species clusters, suggesting that relatively recent integration events, more than ancestral transmission (Supplementary Figure 7) govern the ERV diversity. While, most ERVs are simple retroviruses, consisting of Gag, Pol and Env genes, we found an unusual diversity of complex retroviruses in bats, which are generally rare in endogenous form[56-58] (Supplementary Figure 7). We detected a clade of 5 *Rhinolophus* Pol sequences clustered together with reference foamy retroviruses – Feline Foamy Virus (FFV) and Bovine Foamy Virus (BFV). Foamy retroviruses in bats were detected before from metagenomic data from *Rhinolophus affinis*[59], however, until now it was unclear whether these sequences represented exogenous or endogenous viruses[60]. With the detection of these sequences, we can now confirm the presence of endogenous spumaretroviruses in the *R. ferrumequinum* genome, which furthers our understanding of the historical transmission dynamics of this pathogen. We also detected Pol sequences in the *Molossus* genome clustering closely with reference delta sequences (Bovine Leukemia virus – BLV, Human T-lymphotropic Virus – HTLV). Pol regions for delta retroviruses in bats have not been detected before, with only partial Gag and a single LTR identified previously in *Miniopterus* and *Rhinolophus* species[57,61].

Overall these results show that bat genomes contain a surprising diversity of ERVs, with some sequences never previously recorded in mammalian genomes, confirming interactions between bats and complex retroviruses, which endogenize exceptionally rarely. These integrations are indicative of past viral infections, highlighting which viruses bat species have co-evolved with and tolerated, and thus, can help us better predict potential zoonotic spillover events and direct routine viral monitoring in key species and populations. In addition, bats, as one of the largest orders of mammals, are an excellent model to observe how co-evolution with viruses can shape the mammalian genome over evolutionary timescales. For example, the expansion of the *APOBEC3* genes in bats reported in this and other studies, could be a result of a co-evolutionary arms race shaped by ancient retroviral invasions, and could contribute to the restriction in copy number of endogenous viruses in some bat species. Given that these findings were generated from only six bat genomes we can be confident that further cross-species comparison with similar quality bat genomes will bring even greater insight.

4. Genome evolution

4.1 Identification and alignment of one-to-one orthologs across Placentalia

Human transcripts were projected to 41 additional mammal species (Supplementary Table 1) using TOGA as described above. To avoid aligning non-homologous exons that belong to different transcripts, we selected a single representative transcript for each gene. Selection of the representative transcript was guided by the goal of selecting a transcript with an intact reading frame in our six bats to ensure properly aligned coding regions for these bats. To this end, we considered for each human gene all Principal APPRIS isoforms that were inferred to be 1:1 orthologs in any bat species. In the case where no or multiple Principal isoforms were determined, we considered the longest annotated transcript as a candidate. If this transcript did not contain an intact open reading frame (presence of internal stop codons in all three forward frames or >20% ambiguous bases (N's)) in all six bats, we discarded this transcript as a candidate for the representative transcript and replaced it with a functional alternative isoform where possible. The coding sequences of the final representative transcripts were then extracted from human and the 41 other species using the CESAR 2.0[20] mapping. Individual species were ignored if the representative transcript did not contain an intact reading frame.

To align coding sequences, we used the "alignSequences" module of MACSE (v2.01)[62], trimming potential non-homologous fragments from individual sequences using its "trimNonHomologousFragments" module. Sequences which contained an in-frame stop codon after alignment were removed. Alignments were retained if they contained at least one Yinpterochiroptera and one Yangochiroptera species. This resulted in a final set of 12,931 coding alignments, having a median coverage of 44 mammals.

4.2 Phylogenetic analysis

4.2.1 Phylogenetic inference and divergence time estimation

The best-fit model of sequence evolution for each of the 12,931 nucleotide alignment files was determined using ModelFinder[63] (Supplementary Table 6), which is part of IQ-TREE (v1.6.10)[64], with species trees inferred using the maximum-likelihood (ML) method of phylogenetic reconstruction. A nucleotide supermatrix was generated by concatenating all 12,931 alignments into a single file, which was used as input to infer a mammalian species tree using IQ-TREE and using model partitions for each gene. Branch-support values were determined using UFBoot (v2.0.0)[65] with 1000 bootstrap replicates. The tree was rooted with Atlantogenata (*Trichechus manatus*, *Loxodonta africana*, *Orycteropus afer*, *Echinops telfairi*, *Dasypus novemcinctus*) as a sister group to all other clades. This topology was then used to establish a time tree using r8s (v1.81)[66] and the Langley-Fitch (LF) ML method with Truncated Newton (TN) optimization to find objective function optima. We constrained 14 nodes with fossil calibrations[67], as shown in Supplementary Figure 14. The final divergence time estimate of the last common ancestor of bats (63.38 Mya, Supplementary Table 39) is similar to previous estimates (64 Mya in [1], 66.5 Mya in [68]). This time tree was used to infer coding gene and miRNA family expansion and contraction (see Supplementary Notes 4.6 and 5.2.1). In addition to coding sequences, the position of bats within Laurasiatheria was further investigated using 10,857 orthologous conserved non-coding elements (CNEs), using the aforementioned concatenation method.

Given that two very short branches at the base of Scrotifera define relationships between its four major clades ((Carnivora + Pholidota), Cetartiodactyla, Chiroptera, Perissodactyla), this region of the placental tree may be in the "anomaly zone", defined as a region of tree space where the most common gene tree(s) differs from the species tree topology[69]. In the case of four taxa and a rooted pectinate species tree, anomalous gene trees should be symmetric rather than pectinate. To explore how different genes may impact the tree space, we carried out topology tests that compare all 15

possible Laurasiatheria topologies to each individual protein-coding gene partition or CNE alignment and their concatenated supermatrices, using approximately unbiased (AU) tests[70] as implemented in IQ-TREE. The 15 possible Laurasiatheria topologies that all have Eulipotyphla constrained as basal, and Carnivora and Pholidota constrained as sister orders, are shown in Supplementary Figure 4 and Supplementary Table 40. The number of protein-coding genes supporting each topology as the most likely tree ranged from 476 (Tree 9) to 1,007 (Tree 1), with 2,104 genes showing more than one topology as equally likely. Only 1,173 CNE alignments supported one unique topology (Supplementary Table 41). The AU-tests of the protein-coding supermatrix and the 15 topologies rejected all but Tree 1, while the CNE supermatrix rejected all but Tree 1 and Tree 2.

This suggests that the majority of the data supports a sister relationship between Chiroptera and the other Scrotifera. That said, there were four other topologies that had support from >800 genes (Tree14 882/10822; Tree04 862/10822; Tree15 820/10822; Tree13 806/10822) (Extended Data Fig. 5b). However, even with similar support levels for several topologies, the phylogenetic position for Chiroptera is pectinate on the most common gene tree and does not qualify as anomalous. If the base of Scrotifera is in the anomaly zone, as suggested by coalescence analyses of retroposon insertions[71], then we may expect the most common gene tree(s) to be symmetric rather than pectinate. We may also expect the species tree based on concatenation to be symmetric instead of pectinate[69]. One explanation for the absence of anomalous gene trees, and for a pectinate species tree based on concatenation, is that both protein-coding genes and CNEs are generally under purifying selection, which reduces both coalescence times and incomplete lineage sorting relative to neutrally evolving loci[72,73].

Model misspecification due to an inadequate fit between phylogenetic data and the model of sequence evolution used can cause biases in phylogenetic estimates[74]. To assess whether model misspecification or loss of the historical signal[75] might have been a contributing factor to our phylogenetic estimate (Fig. 2), we examined the 12,931 alignments of protein-coding genes for evidence of violating the assumption of evolution under homogeneous conditions (assumed by the phylogenetic methods used in this paper) and for evidence that the historical signal has decayed almost completely (due to multiple substitutions at the same sites) (Supplementary Table 7). Either of these two cases imply that the data provided by such a gene may not be fit for phylogenetic analysis. To detect model misspecification and loss of historical signal, we used Homo 2.0 (https://github.com/lsjermiin/Homo2.0) and Saturation 1.0 (https://github.com/lsjermiin/SatuRation.v1.0), respectively. For each of the 12,931 protein-coding genes and each codon site within these genes (including unlinked 1st and 2nd codon sites), we surveyed the alignment, using the match-pairs test of symmetry[76] for evidence of violating the assumption of evolution under homogeneous condition. Likewise, these datasets were analysed for evidence of saturation of substitutions at variant sites.

A majority of the datasets were found to violate the phylogenetic assumption of evolution under homogeneous conditions (1st codon sites: 29.0%; 2nd codon sites: 13.5%; 3rd codon sites: 88.8%; 1st + 2nd codon sites: 41.7%; amino acids: 5.1%), implying that many of the datasets have evolved under more complex conditions than assumed by the models of sequence evolution used (note that concatenation of alignments does not mitigate the problem identified). The problem of loss of historical signal was less pronounced (1st codon sites: 4.4%; 2nd codon sites: 10.2%; 3rd codon sites: 6.1%; 1st + 2nd codon sites: 3.1%; amino acids: 3.4%). Based on these observations, and the requirement of having a sequence from all 48 species (many of the genes did not have a complete sequence for all species), we selected 1st + 2nd codon sites from 488 genes. A concatenation of these datasets was deemed fit for phylogenetic analysis assuming evolution under homogeneous conditions, and thus was subjected to the methods above. This concatenated supermatrix consisted of 241,098 sites and 37,588 parsimony-informative sites, and inferred a tree using methods described above (Extended Data Fig. 5c). However, these reduced data did not provide a clear phylogenetic estimate. The best-supported tree differed in its position of Chiroptera, which is now sister to Carnivora + Pholidota, but with a low bootstrap support of 59% (Extended Data Fig. 5c; topology 13 in Supplementary Figure 4). Furthermore, the phylogeny inferred from the subset of 488 genes is also

symmetric for the four major lineages of Scrotifera, as may be expected if this node is in the 'anomaly zone' and therefore concatenation is misleading.

Therefore, we further explored the position of bats in Laurasiatheria under a model of coalescence using SVDquartets[77], as implemented in PAUP* (v4.0b10, Swofford 2003), with 500 bootstrap pseudoreplicates. SVDquartets is a single-site coalescence method that is ideally applied to unlinked sites. However, this method also performs well with multigene alignments[78]. Importantly, SVDquartets avoids problems with the recombination ratchet and gene tree reconstruction error that negatively impact sequence-based coalescence analyses with gene trees[79-81]. The tree topology inferred under a coalescence model showed the same branching pattern for laurasiatherian orders as Tree 1, with bats as sister taxa to Fereuungulata (Extended Data Fig. 5d). The position of Tupaia recovered in this topology (sister to Primates) is identical to the CNE topology (Extended Data Fig. 5a), but differs from the concatenation topologies based on 12,931 protein-coding genes and 488 genes that fit model assumptions where Tupaia is sister to Glires (Fig. 2 and Extended Data Fig. 5c).

4.2.2 Exploring the impact of misalignment and incorrect homology on supermatrix topology

To explore the impact of potential misalignment and incorrect homology statements on gene tree and species tree topology, the distances between all gene trees for the 12,931 alignments and the inferred supermatrix species tree (topology 01) were computed using the Robinson-Foulds (RF) distance metric[82]. The RF distances, determined here as sum total of splits present in the gene tree but not species tree and splits present in the species tree but not gene tree, were computed using the *treedist* function, part of the '*Phangorn*'[83] library in *R* (Supplementary Table 42). RF distances can range from zero (no difference) to $2n$-6 (maximum dissimilarity). All trees used were unrooted. If a gene tree contained fewer taxa than the species tree, the missing taxa were clipped from the species tree using the R package '*ape*'[84].

Homology error, where a non-orthologous exon sequence was included in the alignment for at least one species, can mislead phylogenetic inference. To estimate the frequency of putative homology error in our datasets, the genes with the highest 100 RF-distances were visually inspected and all putative cases of homology error were carefully investigated. This analysis revealed that nine out of the 100 gene alignments showed evidence of homology errors after conservative classification (*AC007375*, *AC093423*, *PAQR3*, *RPL38*, *SUMO2*, *UBE2D3*, *UBE2V1*, *UBE2W*, *YAF2*). Most of these cases involve a very short (often < 30bp) non-homologous first or last coding exon, thus affecting relatively few bases in the overall gene alignment. Some of these cases were caused by incompleteness of current mammalian genome assemblies, where an assembly gap covers the real exon and CESAR 2.0 detects a sufficiently similar but non-homologous exon candidate in the vicinity. Importantly, for seven of these alignments, homology error affects only one species, and is thus less likely to cause an incorrect grouping of unrelated taxa. Only two cases (*AC007375*, *YAF2*), showed homology errors in more than one taxon. Thus, we estimate that homology errors are rare in our dataset and very infrequently affect more than one taxon.

To investigate whether these cases of homology error may have an effect on our estimated phylogenies, we removed the genes with the highest 100 (0.77% of 12,931) or the highest 500 (3.87% of 12,931) RF-distances. In addition, we also removed 112 genes having sequences for fewer than 20 taxa, and thus potentially insufficient phylogenetic signal. IQTREE was used to infer the species tree using the reduced datasets (212 genes removed: 21,218,095 columns, 7,856,816 parsimony-informative sites; 612 genes removed: 21,027,949 columns, 7,808,153 parsimony-informative sites). We found that removing these genes with distant tree topologies and low phylogenetic signal had no effect on the overall species tree (Fig. 2), with all inferred evolutionary relationship maintained, and negligible effects on all branch lengths (see Extended Data Fig. 4). This provides evidence that, even though misalignment and homology errors exist in our dataset, our overall phylogenetic inference is robust.

Taken together, multiple lines of evidence show the highest level of support for Chiroptera as basal in Scrotifera and sister to Fereuugulata (Fig. 2). However, different regions of the genome can and do support alternative evolutionary scenarios. This highlights the importance of generating phylogenetic inferences from multiple genomic regions and the importance of screening these regions for violations of phylogenetic assumptions and incongruent signals, especially when dealing with short internal branches.

4.3 Selection test

4.3.1 Genome-wide screen for signatures of positive selection

First, the aBSREL[85] model implemented in the Hyphy package (v2.3.11) was used to identify genes that have experienced episodic selection during the evolution of bats. For each alignment, we pruned the phylogeny, estimated from our amino acid supermatrix, to include only those species present in the gene alignment. All branches in the bat subtree were labelled as test branches. For each gene, aBSREL produces a corrected P-value if multiple branches are tested. This branch-corrected P-value was extracted for each branch tested. To account for the fact that our genome-wide screen for selection considered 12,931 genes, we further corrected the branch-corrected P-values by computing a false discovery rate (FDR) using the p.adjust tool and the Benjamini–Hochberg procedure in R (v3.3.1)[86], with an FDR cut-off of 0.05. We retained genes found to be under selection only at the bat ancestor and not elsewhere in the bat subtree. Second, the branch-site test for positive selection implemented in codeml from the PAML software suite (v9.4)[87], was used to independently verify selection (FDR < 0.05) in genes identified under aBSREL and to identify putatively selected sites. To assure correctness of our homology statement, we manually inspected the alignment of all genes with significant evidence from aBSREL and codeml to detect obvious alignment errors. In addition to manual inspection, we used T_Coffee[88] to confirm a high quality of the entire alignment. Furthermore, we carefully inspected the neighbourhood of sites reported to be under selection and used T_Coffee to confirm a high alignment quality at these selected sites. For genes, where manual inspection or T_Coffee found putative alignment ambiguities, we produced a manually-adjusted alignment and re-ran aBSREL and codeml. We only reported selection in a gene if its manually-adjusted alignment also showed significant evidence for selection (aBSREL FDR < 0.05, codeml FDR < 0.05). For example, for the gene *TJP2,* a region of potential alignment ambiguity was identified during manual inspection. The alignment produced by MACSE produced significant evidence for positive selection (aBSREL P-value = 1.3×10^{-7}, FDR = 0.002), while the manually adjusted alignment lowered significance (aBSREL P-value = 0.009, FDR = 0.813). However, the manual adjustment revealed a possible echolocator specific insertion (Extended Data Fig. 6b), which is not considered as all insertions/deletions are generally ignored by phylogenetic tests for positive selection. All final alignments of genes with significant evidence for positive selection after manual curation are provided in Supplementary Data File 2.

These analyses revealed 9 genes with a robust signal of positive selection at the bat ancestor (Supplementary Table 8). While these 9 genes have diverse functions, they included two genes with hearing-related functions, which may relate to the evolution of echolocation. These genes, *LRP2* (low-density lipoprotein receptor-related protein 2, also called megalin) and *SERPINB6* (serpin family B member 6) are expressed in the cochlea and associated with human disorders involving deafness. LRP2 encodes a multi-ligand receptor involved in endocytosis that is expressed in the kidney, forebrain and, importantly, is also expressed in the cochlear duct[89]. Mutations in this gene are associated with Donnai-Barrow Syndrome, an autosomal recessive disease with symptoms including sensorineural deafness[90], and progressive hearing loss has also been observed in *Lrp2* knockout mice[91]. Similarly, *SERPINB6* is associated with non-syndromic hearing loss and this serine protease inhibitor is expressed in cochlear hair cells[92,93]. Sites identified as having experienced positive selection at the bat ancestor showed bat specific substitutions in both genes. Interestingly, the laryngeal echolocating bats showed a specific asparagine to methionine substitution in *LRP2*. In *Rousettus*, the only non-laryngeal echolocator in our six bats, this site has been substituted for a threonine. Combined with analysis of 6 other publicly available bat genomes (n=6), we confirmed the presence of a methionine

in all laryngeal echolocating bats (n=9) and a threonine residue in pteropodids, which do not have laryngeal echolocation (n=3) (Extended Data Fig. 6a).

4.3.2 Candidate genes and selection tests

A list of human ageing-related genes was collated from GenAge[94]. To augment these ageing-related genes and identify genes associated with immunity and metabolism, we queried the Gene Ontology (GO) database, AmiGO[95], with 'ageing', 'immunity' and 'metabolism' as search terms. A total of 2,453 genes were investigated across all 6 bats, using the same alignments as for the genome-wide screen (Supplementary Table 43).

Each of the 2,453 gene alignments was analysed for signatures of positive selection using the branch-site test. All branch-site tests were carried out using codeml, inferring the likelihood-derived dN/dS (ω) values under both the null (ω1, ω2 constrained to be less than 1) and alternative (ω2 can vary) hypotheses. As branch-site tests require a species tree, analyses were carried out using the best-supported mammal topology, displayed in Fig. 2, with the ancestral bat lineage designated as foreground branch. A likelihood ratio test (LRT = 2*(lnL alt – lnL null)), comparing the fit of both null and alternative log-likelihood values, was carried out for each alignment. P-values were then calculated assuming a chi-squared distribution[87] and corrected for multiple testing using FDR correction (p.adjust tool and the Benjamini–Hochberg procedure in R[86]). Only significant genes at an FDR cut-off of 0.05 having ω greater than 1 were considered for further interpretation. Sequence-specific sites undergoing positive selection were identified based on significant Bayes Empirical Bates (BEB) scores obtained from codeml (P-value > 0.95), and a subsequent visual inspection of alignments to rule out false-positive results due to potentially misaligned sequences. Significant genes showing ω values greater than 1, but with no identifiable BEB sites, were also reported (Supplementary Table 9). Additionally, while six of the 15 genes showing significant P-values in HyPhy were included in the candidate list of 2,453 (*AZGP1*, *CXCL13*, *GLB1*, *HP*, *LRP2*, *SERPINB6*; see 4.3.1; Extended Data Fig. 6c), there were nine genes that were not (*APOBEC3H*, *C17orf78*, *INAVA*, *KBTBD11*, *NES*, *NPSR1*, *PALB2*, *TGM2*, *TRUB2*). These extra genes were independently explored for selection using codeml. P-values from these extra genes were added to the 2,453 genes and the entire set of 2,462 P-values was corrected for multiple testing using FDR correction as reported above (p.adjust tool and the Benjamini–Hochberg procedure in R[86]). Only significant genes at an FDR cut-off of 0.05 having ω greater than 1 were considered for further interpretation. Sequence-specific sites undergoing positive selection were identified based on significant Bayes Empirical Bates (BEB) scores obtained from codeml (P-value > 0.95), and a subsequent visual inspection of alignments to rule out false-positive results due to potentially misaligned sequences. Significant genes showing ω values greater than 1, but with no identifiable BEB sites, were also reported (Supplementary Table 9).

A total of 23 out of the 2,453 genes relating to ageing, immunity and metabolism showed evidence of positive selection in the ancestral bat lineage using codeml in PAML (Supplementary Table 9). Branch-site tests showed evidence of positive selection in the ancestral bat branch for genes associated with immune system modulation including both *IL17D* and *IL-1β*: cytokines playing roles in recruitment of natural killer cells to tumours[96] and the proinflammatory response, respectively. *IL-1β* has also been shown to up-regulate *DEFB1*[97], an antimicrobial defensin also significant in our analyses. Similarly, *CXCL13* connects innate and adaptive immune systems, promoting B-cell survival and maturation[98], and elevated levels are associated with autoimmune inflammation[99]. Positive selection was found in *SEMA4D*, which plays a role in the regulation of the humoral immune response[100]. It is also involved in the interaction between T-cells and antigen-presenting cells (APCs). These APCs can be activated by *TSLP*[101], another gene showing evidence of positive selection in the ancestral bat branch. Genes involved in the recognition and response to pathogens such as *GP2, MRC1, TLR9, LCN2*[102-104] also show evidence of bat-specific positive selection. Though not showing any specific adaptive sites, *PURB* had signatures of selection. In addition to a role in cell proliferation, *PURB* also regulates *MYC*[105,106], an oncogene shown to be under divergent selection in bats[107] and

which exhibits a unique anti-ageing transcriptomic profile in long lived *Myotis* bats[108]. Selection was found in *NR1H2*, encoding the Liver X receptor β receptor. This receptor is activated by lipophilic ligands, such as oxysterols, binds to DNA and can interfere with the NF-kB signalling pathway, suppressing pro-inflammatory responses[109]. Additionally, *NR1H2* also regulates cholesterol transport and metabolism in the liver, thus demonstrating both immune and metabolic activity[110]. Nine of the genes showing significant PAML results were detected in HyPhy (see Supplementary Note 4.3.1), showing a robust signal and agreement between different methods.

To further investigate overlap between both the aBSREL and codeml methods of selection analysis, P-values estimated for the subset of 2,453 candidate genes, taken from genome-wide screen of 12,931 genes with the HyPhy suite of software, were FDR corrected and compared with results from codeml in PAML. A total of 14 out of the 23 genes showing signatures of selection with codeml overlapped with those significant using aBSREL (Extended Data Fig. 6c), and included the aforementioned *LRP2*, *SERPINB6*, *IL17D*, *IL-1β*, *GP2*, *LCN2* and *PURB*. The remaining nine genes showing significance with codeml that did not overlap had P-values less than 0.05 before FDR correction. Combining both genome-wide and candidate gene screen approaches to selection analyses has therefore identified robust signals of adaptive evolution in the ancestral bat for key genes involved in both the function and regulation of immunity and the ability to tolerate various types of pathogens.

4.3.3 Selection in non-chiropteran branches

To explore whether the biological pathways under positive selection in the bat ancestor were unique to bats we applied the same methods (see Supplementary Notes 4.3.1 and 4.3.2) to both the ancestral Carnivora (HyPhy n=12,821; candidate genes n=2,436) and Cetartiodactyla (HyPhy n=12,866; candidate genes n=2,443) lineages. Genome-wide screens using aBSREL detected 19 and nine genes showing evidence of positive selection for Carnivora and Cetartiodactyla, respectively (Supplementary Table 10), while codeml identified 22 and 12 genes (Supplementary Table 10). Using the same strict requirements for detecting selection in the ancestral Chiroptera lineage (see Supplementary Note 4.3.2), we identified seven genes in Carnivora (Supplementary Figure 15; *CD86*, *ICAM1*, *PGA3*, *PGA4*, *PGA5*, *RAMP2*, *TLR6*) and six genes in Cetartiodactyla (Supplementary Figure 15; *CD300LG*, *FAM71B*, *RAB11FIP3*, *SPIB*, *TMEM176B*, *TRIM56*) showing a robust signal of lineage-specific positive selection.

In Carnivora, three genes in the pepsinogen A gene cluster (*PGA3*, *PGA4*, *PGA5*) had evidence of positive selection, possibly reflecting the evolution of a carnivorous diet. All three *PGA* genes have the same residue showing significant Bayes Empirical Bayes (BEB) scores, implying that positive selection may have occurred in the ancestral pepsinogen sequence, which was subsequently maintained across numerous instances of paralogous gene duplication[111]. Carnivora had fewer instances of positive selection in genes relating to immunity, showing selection in CD86 (Adaptive immune response, stimulating T-cell activity[112]), ICAM1 (migration of leukocytes during inflammation[113]) and TLR6 (non-viral pathogen sensing[114]). Of the six genes showing a robust signal of selection in Cetartiodactyla, four were involved in the immune system: CD300LG (regulation of immune response[115]), SPIB (dentritic and INF-producing cells[116]), TMEM176B (dendritic cell differentiation[117]) and TRIM56 (innate immune response[118]). While none of the genes showing robust signals of selection in bats had evidence of sequence-specific positive selection in other Laurasiatherian lineages tested, repeating the stringent genome-wide screen to detect selection on comparable, ordinal branches leading to the ancestors of Carnivora and Cetartiodactyla revealed fewer immune-related genes.

Using the candidate gene approach, we identified *LRP2* as under selection at the base of Cetartiodactyla. *LRP2* was also initially suggested to be under selection by HyPhy ($p<0.001$) using the genome-wide screen, however, was removed after filtering for genes showing selection on multiple branches within the same clade (note that we consistently applied this filter to Chiroptera too). Specifically, signatures of selection were found along the tip branches leading to cow (*Bos taurus*) and camel (*Camelus ferus*), and the branch leading to artiofabula (Cetartiodactyla excluding

Camelidae). For this reason, we excluded *LRP2* as a robust candidate for ancestral selection in cetartiodactyla. HyPhy also suggested the Carnivora may show selection in *LRP2* (p=0.048), however, this signal was not recapitulated using PAML. Importantly, the sites that are under selection in the Chiropteran ancestor are different to the sites under selection in the Cetartiodactyla ancestor. This shows that the large *LRP2* gene can be a target of selection in multiple lineages; however, potentially different sites and functions may be selected for. Thus, experimental studies are required to reveal which functional aspect of *LRP2* has been altered by the selected changes in bats.

4.4 *In silico* analyses of protein structure

In order to explore the effects of positive selection further, all amino acid sequences showing bat-specific instances of positive selection with significant Bayes Empirical Bayes (BEB) scores (Supplementary Table 44) had their 3D structure modelled using *in silico* methods. For each of these 21 alignments, the bat taxon with the most complete gene coverage was chosen as the target sequence. Protein structures were predicted using the Iterative Threading ASSEmbly Refinement (I-TASSER[119,120]) server. I-TASSER identifies reference PDB templates showing similar super-secondary structures to the target amino acid sequences using the Local Meta-Threading Server (LOMETS)[121], building unaligned (e.g. loop) regions via *ab initio* modelling. If no appropriate template is found, I-TASSER builds the whole structure using *ab initio* modelling. In addition to the bat taxon, all human structures were also modelled. For each amino acid sequence, the model with the highest estimated confidence score (C-score; Supplementary Table 44) was used for all downstream analyses (Supplementary Table 44). Bat and human structures were compared by superimposing both via a pairwise alignment and subsequent fitting of residue pairs using the MatchMaker function in UCSFChimera[122]. Root-Mean-Square Deviation (RMSD) of atomic positions between the two structures were also calculated (Supplementary Table 44). Putative ligand binding sites were predicted for each protein using the COFACTOR and COACH methods[123-125], which utilize structural comparisons, protein–protein interaction networks and known ligand-binding templates, to predict binding sites within the I-TASSER software suite. The predicted sites were cross-references with the loci showing evidence of positive selection.

C-scores for each bat model ranged from -4.45 (*C17orf78*) to 1.42 (*SERPINB6*), with higher values indicating higher confidence (Supplementary Table 44). When overlapping human and bat models, the predicted structure showing the most overlapping atomic pairs and lowest RMSD was *TLR9* (overlapping atomic pairs: 0.223Å, RMSD: 740 pairs; Supplementary Table 44). When cross-referencing sites showing evidence of positive selection with predicted ligand binding sites, bat-specific sites under selection in four genes (*DEFB1*, *LCN2*, *SERPINB6*, *KBTBD11*) were identified as ligand-binding residues, while eight genes (*AZGP1*, *ICOSLG*, *IL17A*, *IL17D*, *SEMA4D*, *TLR9*, *INAVA*, *NPSR1*) had predicted ligand-binding sites within three residues upstream or downstream of positively-selected sites in the primary amino acid sequence. Due to its size (4653 amino acids in *R. ferrumequinum*) and the maximum modelling limit on I-TASSER's online server (1500 amino acids), *LRP2* could not be modelled in full. Therefore, a smaller region, consisting of 750 sites upstream and downstream of each residue under selection was modelled for *R. ferrumequinum*, *R. aegyptiacus*, *P. kuhlii* (Supplementary Table 44).

When comparing the residues at the 37 sites under selection in bats (Supplementary Table 44) to their relative residue in humans, it was found that 21 of these sites had amino acids with differing side-chain properties (e.g. a polar-uncharged asparagine in bats relative to a hydrophobic leucine in humans for *SERPINB6*, Supplementary Figure 16). The impact of these bat-specific amino acid changes on the overall protein structure were further explored by determining the predicted changes in folding free energy (ΔΔG), between 'wild-type' bat variants and human 'mutants' using the Dynamut suite of software[126], and the I-TASSER predicted 3D structures. By calculating a predicted increase or decrease in this Gibbs free energy, it was determined whether the bat residues undergoing positive selection were stabilizing or destabilizing, by replacing them with the human amino acid at that loci. Twenty mutations from bat wild-type to human residue (excluding LRP2 fragments) resulted in a net

de-stabilizing effect, indicating a more stable bat residue relative to the human sequence. Conversely, replacing the human residue with the positively-selected bat amino acid resulted in a net-stabilizing effect on the overall human protein structure. Not all bat-specific incidents of positive selection gave rise to higher stability. In the case of *SERPINB6*, replacing the bat-specific asparagine at residue 108 with leucine, found in human and the 23 other taxa explored for this gene, resulted in a slight increase in stability through the addition of ionic bonds and hydrophobic contacts (Supplementary Figure 16). This would suggest that the bat *SERPINB6* structure is less stable than the human version. However, the difficulty in calculating free energy across multiple sites at once means that the greater context of such a mutation cannot be explored using *in silico* methods. A similar pattern was found with the majority of bat-specific residues for *INAVA* (Supplementary Figure 17 and Supplementary Table 44), with multiple stabilizing and destabilizing mutations across positively selected sites.

The effect of ‘non-bat’ asparagine (N), and the non-echolocating threonine (T) compared to the echolocating methionine (M) in *LRP2* at amino acid site 1564 under selection was explored using the modelled fragments for *R. ferrumequinum* (echolocating), *R. aegyptiacus* (non-laryngeal echolocating) and *P. kuhlii* (echolocating). In all instances, the bat-specific methionine/threonine represented a more stable residue, as replacing it with the ‘non-bat’ asparagine resulted in a destabilizing increase of free energy (Supplementary Table 44). Interestingly, when replacing the ‘non-echolocating’ threonine with the ‘echolocating’ methionine, the overall effect was stabilizing, resulting in a decrease of molecule flexibility and thus Gibbs free energy. This suggests a higher degree of stability in the ‘echolocating’ protein sequence. However, as the mutation did not fall within a predicted binding domain, and the structure represents roughly 33% of the full protein, further effects of the potential function of *LRP2* in echolocation cannot be explored *in silico*.

In summary, by changing stability or introducing different amino acid side-chains, the selected mutations observed in bat proteins may affect function. Given the computational complexity of exploring the effects of multiple mutations on structure and dynamics, it remains unclear what additional effects such sites under selection might have, especially in the context of additional mutations showing signals of selection or insertion/deletion events. However, future work using gene editing, such as CRISPR, may allow *in vivo* validation of these results beyond computational predictions. The information of predicted 3D protein structures of all candidate genes are available in Supplementary Data File 3.

4.5 Systematic screen for gene losses

To search for gene losses that occurred in the stem Chiroptera branch, we used a previously-developed approach to detect gene-inactivating mutations[127]. This approach uses whole genome alignments between a reference (here human hg38 assembly) and the six bat genomes presented here to detect large deletions that cover exons or entire genes, insertions and deletions that shift the reading frame, mutations that disrupt donor (GT/GC) or acceptor (AG) splice site dinucleotides, and mutations that create premature stop codons. To overcome issues related to genome assembly and alignment and evolutionary changes in the exon–intron structures of conserved genes, this approach performs a series of filter steps to exclude false inactivating mutations. Specifically, the approach (i) only considers those unaligning or deleted exons or genes where the respective locus does not overlap an assembly gap in the other genome, (ii) realigns all coding exons with CESAR, a Hidden Markov Model method that considers reading frame and splice site information to produce an intact exon alignment whenever possible[20,128], (iii) excludes alignments to paralogous genes or processed pseudogenes, and (iv) considers all principal or alternative APPRIS isoforms of a gene[30] and outputs the isoform with the smallest number of inactivating mutations. Our screen for lost genes is based on the human Ensembl v96 gene annotation[129]. The maximum proportion of the reading frame that remains intact for any transcript for each gene was also calculated.

To extract genes likely lost in stem Chiroptera, we filtered for genes for which less than 80% of the ORF is intact in all six bats. We excluded genes that are classified as lost in more than 20% of

the non-Chiroptera Laurasiatherian mammals contained in our multiple genome alignment[34] (https://bds.mpi-cbg.de/hillerlab/120MammalAlignment/Human120way/). While we confirmed previous findings that *PYHIN* genes (*MNDA*, *PYHIN1*, *IFI16*, *AIM2*) are completely deleted in Chiroptera[130] (Supplementary Figure 18), we excluded these genes in our screen because they were not intact in at least 80% of non-Chiroptera laurasiatherians. For the lost genes listed in Supplementary Table 11, we manually inspected the genome alignment chains to confirm that the remnants of the lost gene are located in a context of conserved gene order and to rule out that a duplicated intact copy of these genes exist in bats.

4.6 Protein Family Evolution

To investigate expansions and contractions of protein families, we used CAFE (v4.0)[131]. CAFE requires annotated proteins assigned to families. To this end, we downloaded GFF3 files from Ensembl which were available for 25 of the 42 considered species. To obtain a single isoform from each gene, we used genePredSingleCover (https://github.com/ENCODE-DCC/kentUtils.git) to obtain the longest transcript from each locus, and translated this transcript to a peptide sequence. The POrthoMCL pipeline (https://github.com/etabari/PorthoMCL, accessed 12/6/2019; commit dec8e5f), a parallel implementation of the OrthoMCL algorithm, was used to cluster proteins into families. All families were assigned PANTHER Database (v14.0) IDs, based on the human genes contained in a family. Families assigned the same ID were merged. Where POrthoMCL families were composed of multiple families, all families with overlap based on PANTHER IDs were merged. To obtain families that were already present in the Placentalia root, we retained those families where at least one member was present in all bats, at least one of human, mouse or rat (representative Euarchontoglires) and one Atlantogenata species. Families which varied in size by more than 100 members between the species with the highest and lowest count were also removed. CAFE was then used to identify families which underwent expansion or contraction at the base of bats, using the previously produced ultrametric time tree (see Supplementary Note 4.2 above). The caferror.py function was used to estimate an error model for the data, which was used in further analysis. A single lambda, or birth/death parameter, was inferred for the entire tree. Families were retained if estimated to have undergone a significant expansion or contraction at the ancestor of all bats with an FDR value < 0.05 (Supplementary Table 12).

4.6.1 Evolution of the APOBEC3 gene cluster

Gene family PTHR13857 showed evidence of expansion along the ancestral bat lineage. In order to identify which family members had expanded in bats, a phylogenetic tree was constructed from all proteins assigned to PTHR13857. Protein sequences were aligned using the G-INS-i algorithm in MAFFT (v7.310)[132]. A phylogenetic tree was constructed using PhyML (v20120412)[133] using the BLOSUM62 substitution matrix, with 4 gamma distributed rate categories, and invariant sites. The APOBEC3 genes were found to be expanded within this family in bats. The APOBEC3 proteins from bats were classified into three classes based on the Z-domain, Z1, Z2 and Z3 using previously published motifs[134]. The Z2B motif, previously observed in Pteropid bats, was also used in classification[135]. Manual inspection of unclassified APOBEC3 proteins revealed small changes in the length of the linker region between functional residues and adjusting this allowed classifying these previously unclassified proteins. Finally, a Z1B motif observed in *P. kuhlii* (HxEx5xxx18-19SWSPCx2Cx6Fx8Lx5xxxx5-9Lx2Lx9M) was produced by modifying the canonical Z1 (HxEx5xxx18-19SWSPCx2Cx6Fx8Lx5RIYx9Lx2Lx9M). Those which remained unclassified were manually assigned to a class. All motifs used to classify APOBEC3 proteins are given in Fig. 3c. Proteins were also designated as likely non-functional if they did not contain a deoxycytidine deaminase domain motif, $HxEx_{24\text{-}33}PCxxC$. In order to understand the duplication history of the APOBEC3 proteins, the Z domains from all bat APOBEC3 proteins were aligned using MAFFT and a phylogeny constructed based on amino acid distance using BioNJ (Supplementary Figure 5).

5. Evolution of non-coding genomic regions

5.1 Annotation of conserved non-coding RNA genes

In brief, the conserved non-coding RNA genes were annotated using the Infernal pipeline (v1.1.2)[136]. Initially, transposable elements (TEs) and low complexity DNA regions in each genome (six bat genomes plus seven additional mammalian genomes as outgroups; see Supplementary Table 1) were hard-masked using RepeatMasker (v4.0.9) (http://www.repeatmasker.org) by aligning the genomic sequences against a custom library of known repeats (see Supplementary Note 3.3). This library contains a collection of vertebrate TEs and the most up-to-date bat-specific TEs. It is noteworthy that some regions containing certain tRNA genes, small nuclear RNA (snRNA) genes and their pseudogenes were masked by RepeatMasker due to their high similarity to SINEs. The repeat-masked genomes were queried against the Rfam database (v14.0)[137] using Infernal (v1.1.2)[136] with default parameters. The alignments with an E-value < 10^{-6} were considered statistically significant and their corresponding genomic regions were annotated as conserved non-coding RNA genes. Based on the Rfam database, these candidates were further categorized into ribosomal RNA (rRNA), small nuclear RNA (snRNA), small nucleolar RNA (snoRNA), microRNA (miRNA), and long non-coding RNA (LncRNA). Other RNA types or uncharacterized RNA genes were grouped into miscellaneous RNA (miscRNA). See Fig. 4a for the summary of non-coding RNAs in six bats. The number of conserved ncRNAs that are shared between bats is shown in Supplementary Figure 8.

5.2 The evolution of conserved miRNA gene families

As the miRNA genes were predicted *in silico*, we further investigated the conserved miRNA genes which had no copies in each of these 6 bat genomes using our miRNA-Seq data. Unexpectedly, depending on the species we detected the expression of a few miRNA genes (~2%) which were not predicted by Infernal and were initially considered to be lost. To understand the contradiction, we investigated their genomic loci and noticed that they were masked as transposable elements in the respective genomes in which they actually showed evidence of expression. As these miRNA genes were also supported by stable secondary structures, we regarded them as real miRNA genes rather than transposable element and manually curated them (Supplementary Table 13).

To gain an overview of the evolutionary patterns of conserved miRNA families along the bat lineages, we performed two analyses that investigate (i) expansions or contractions of members with miRNA gene families (see Supplementary Note 5.2.1) and (ii) gains or losses of miRNA families (see Supplementary Note 5.2.2). These analyses compared 48 mammalian taxa (6 bat species plus 42 non-bat taxa; see Supplementary Table 1). The masked genomes of these 42 non-bat species were obtained from NCBI. miRNA gene families were predicted using the same pipeline as described above, and the copy number for each miRNA family was subsequently determined. This pipeline reduced the number of false-positive miRNA predictions, which overlapped with annotated TEs, to a minimal level. We obtained a matrix containing the copy numbers of 286 conserved miRNA families across 48 mammalian species. This dataset was filtered by retaining those miRNA families present at least in one Atlantogenata species and one Boreoeutherian species. As the current miRNA set is biased towards conserved and highly expressed miRNA, the lineage-specific miRNAs could not be discovered via *ab initio* genomic prediction, therefore, were not included in these analyses.

5.2.1 miRNA family expansion and contraction

miRNA family expansion and contraction analysis was carried out using CAFE (v4.2.1)[131]. A random birth and death model was used to infer the evolution of miRNA gene copy number across a user-specified phylogenetic tree. We used the supermatrix tree that was inferred on the basis of the alignments of 12,931 single-copy orthologous genes across 48 taxa (see Supplementary Note 4.1) and calibrated as described in Supplementary Note 4.2. An error model was estimated to correct for genome assembly error. The global parameter lambda, which indicates a universal miRNA birth and death rate across all branches, was estimated using maximum likelihood. A P-value was calculated for

each family. The miRNA families with an FDR value < 0.05 were regarded to have a significantly accelerated expansion and contraction rate. The genomic loci of the miRNA families exhibiting expansions and contractions in bat lineages were manually checked and confirmed.

5.2.2 miRNA gene gain and loss

The gain and loss of miRNA families was inferred by using Dollop from the Phylip software (v3.696) (http://evolution.genetics.washington.edu/phylip/doc/dollop.html). Dollop is based on the Dollo parsimony principle, which assumes an independent evolution in each lineage and irreversibility of gene loss. In the context of this study, it implies that once a miRNA gene family is predicted to be lost in certain lineages, it cannot be regained during evolution. For Dollo inference, the supermatrix tree (see Supplementary Note 4.2) and a binary matrix derived from the matrix used for CAFE analysis (see Supplementary Note 5.2.1) were employed. In this binary matrix, ‘1’ and ‘0’ indicate the presence and absence of each miRNA family in each of the 48 taxa, respectively. The number of miRNA family gain and loss in each branch and node was further extracted using in-house Perl scripts.

To assess the performance of the Dollo parsimony principle, we generated a random matrix of phylogenetic profiles where both miRNA family presence in each species and the phylogenetic tree were shuffled. Based on this matrix, the number of losses required to explain the random profiles was determined by Dollop. We observed a major difference in the number of inferred miRNA losses between real and random data. In particular, the random data resulted in multiple losses, while the real data could be generally explained by a limited number of losses (Supplementary Figure 10). This result supports the Dollo assumption that the evolutionary patterns of most miRNA gene families can be inferred by a single acquisition event. The miRNA families that were gained or lost in the bat lineages are listed in Supplementary Table 45.

5.2.3 Single-copy miRNA alignments across 48 mammals

To ascertain the sequence conservation of these predicted miRNA families between bats and other mammals, we focused on the single-copy miRNA genes across 48 taxa. We only considered the single-copy miRNA genes that were present in at least 80% of all taxa and at least 3 bat species. Based on these criteria, 98 single-copy miRNA genes were investigated and their precursor sequences in each genome were retrieved using Bedtools (v2.25.0)[138], respectively. For each miRNA gene, the precursor sequences were aligned using ClustalW (v2.1)[139] and the alignments were visualized in Geneious (v7.1.9) (https://www.geneious.com). For each miRNA, conservation of the mature 5’ and 3’ sequences and the hairpin loop was further investigated and manually curated (Supplementary Table 46).

5.3 Novel microRNAs that evolved in bats

5.3.1 Small RNA Illumina sequencing from brain, kidney and liver

To identify novel microRNAs that evolved in bats, we used Illumina technology to sequence small RNA libraries from brain, kidney and liver for all 6 bat species (Supplementary Table 17). Briefly, total RNA was extracted from respective tissue types using TRIzol reagents (No. 15596-018, Carlsbad, CA) or the QIAGEN miRNeasy mini kit (Cat. No. 217004), following the manufacturer’s instructions. The quality and quantity of RNA were measured using a Bioanalyzer 2100 (Aligent Technologies). The samples with total RNA > 1 µg and an RNA integrity score (RIN) > 7 were prioritized for Illumina small RNA library preparation. RNA libraries were prepared using the Illumina TruSeq small RNA library preparation kit and were further sequenced on Illumina HiSeq 4000 platforms at the BGI (Hong Kong). Each sample was sequenced to a minimum depth of 30 million 50 bp single-end reads. The information of small RNA sequencing is summarized in Supplementary Table 17. The raw miRNA-Seq data have been deposited in the NCBI SRA database under the BioProject ID: 572574.

5.3.2 miRNA profiling pipeline

Our approach to identify novel miRNAs was based largely on miRDeep2 (v2.0.0.8)[140]. Prior to analyses, the 3' adaptor sequence (5'-TGGAATTCTCGGGTGCCAAGGAACTCCAA-3') and low-quality bases (< Q25) were trimmed from the raw reads using Cutadapt (v1.14). We further filtered the reads with low complexity and only retained the reads ranging from 16 bp to 25 bp in length. Subsequently, identical reads were compressed to single entries with the headers indicating their read counts using Mapper[140]. These unique sequence tags were then mapped to the respective genome and were analysed by miRDeep2 to predict mature miRNAs and their precursor sequences. The prediction was based on the stability of their secondary structures and their sequence similarity to the known miRNA curated in miRBase (release 22)[141]. We considered miRNA candidates, which had the read counts < 5 and the true positive probability < 60%, as unreliable and excluded them from downstream analyses. miRDeep2 categorized miRNA into known and novel groups. We further manually inspected the novel groups by comparing them against the miRBase (release 22)[141]. For each bat species, any miRNA in the novel category, which shared the same seed region (nucleotides 2-7 of the mature miRNA sequence) with a known miRNA, were moved to the respective known groups. This filter ensures that the miRNAs in novel groups have a novel target specificity and potentially a novel repertoire of gene targets.

5.3.3 Identification of known and novel miRNAs in each bat species

To identify known miRNA in each bat species, the raw reads from brain, kidney and liver were first pooled together and the pipeline described above was employed. This pipeline resulted in two categories of predicted miRNAs: known and novel miRNAs. The miRNAs in the known group also exist in other mammalian species in miRBase. In general, novel miRNAs are usually expressed at a lower level than known miRNAs[142], which makes it more likely that authentic novel miRNA are falsely regarded as sequencing noise. To resolve this, we also predicted novel miRNAs for each bat using small RNA-seq data from each individual tissue (brain, kidney and liver). In this second screen, we considered miRNA candidates assigned to the novel group, if their expression was detected in at least two of the three profiled tissues. To achieve this, for each species all novel precursor miRNA predicted from brain, kidney and liver were pooled, and the sequence similarity was calculated using CD-HIT (v4.6.7)[42]. Only miRNAs, whose precursor sequences showed > 95% identity between tissues, were considered as reliable novel miRNA. The number of known and novel miRNAs identified in each species is listed in Supplementary Table 17. For each species we further analysed the genomic coordinates of both known and novel miRNA based on gene annotation using Bedtools (v2.27.0)[138]. The distribution of miRNA locations in exons, introns, 3'UTRs and intergenic regions is given in Supplementary Figure 19. Consistent with the observation in other species, a large proportion of miRNAs are located in the intergenic and intronic regions in these 6 bat genomes (Supplementary Figure 19).

5.3.4 miRNA evolution in bats

To better understand miRNA evolution in bats, we investigated and compared novel miRNAs that evolved in our 6 bat species. miRNAs are often expressed in a time- and tissue-specific manner, which implies that small RNA-seq data may not capture all novel miRNA that are shared among all 6 bats. Therefore, to identify novel miRNAs that are likely shared among all 6 bats but are not present in any other of the 42 mammals, we integrated our small RNA-seq data and sequence similarity searches. Briefly, we first merged all novel miRNA precursors predicted from small RNA-seq data of our bats, and removed redundancy using CD-HIT (v4.6.7)[42]. Next, to identify shared miRNAs by sequence similarity, we mapped these nonredundant novel miRNA precursors to the 6 bat genomes and the other 42 mammalian genomes using bowtie (v2.2.5)[143], with the -N 1 parameter to allow at most one mismatch in the alignment seed. We allowed a maximum of 2 mismatches or indels outside the seed region and required an identical seed sequence. The number of novel miRNA and novel seeds shared across 6 bats was plotted in Supplementary Figure 12. Next, we only kept miRNA

precursors that were successfully mapped to all 6 bat genomes but did not map to any of the other 42 mammalian genomes. Subsequently, the filtered miRNA precursors were compared against the NCBI nucleotide database[47] and miRBase (release 22). We excluded any miRNA that exhibited homology to non-bat genomic sequences (NCBI nt database) and or that exhibited homology to non-bat miRNAs. This approach resulted in 12 novel miRNAs identified in all 6 bats. The details of these 12 novel miRNAs, including precursor and mature sequences, seed regions, expression values in different tissues, and hairpin structures, are listed in Supplementary Table 18.

5.3.5 3'UTR and miRNA target prediction

Our analysis of 3'UTRs inferred from Iso-seq data showed that many genes in each bat species had alternative 3'UTRs (Supplementary Table 15). In order to obtain a comprehensive set of 3'UTRs that maximizes the potential target space for miRNA target prediction, we generated a "pseudo 3'UTR" for each gene per species, defining the pseudo 3'UTR of a gene as the union of all its annotated 3' UTRs. To do this, we used Bedtools (v2.27.0) to merge the coordinates of alternative 3'UTRs for genes with more than one annotated 3'UTR where possible or concatenated different 3'UTRs with 20 'X's if they did not share overlapping coordinates using in-house scripts. For the few cases, where the 3'UTRs of neighbouring genes overlapped in their genomic coordinates, we first separated the 3'UTRs of each gene and processed them separately. The statistics of pseudo 3'UTRs is summarized in Supplementary Table 15.

We found that miR-337-3p has a unique seed region in bats compared to all other 42 taxa (Fig. 4b and Extended Data Fig. 9b). To investigate whether this unique seed alters the predicted target genes, we developed a pipeline to extrapolate different targets of miR-337-3p between bats and human. As miR-337-3p mature sequences are conserved among all bats, we created a 'master list' of 3'UTRs by merging the pseudo 3'UTRs from the 6 bats to predict target genes. This procedure produced a set of 13,083 genes that could be potentially regulated by miR-337-3p in all 6 bats. We used the mature miR-337-3p sequence to predict targets in the master list of 3'UTRs using both miranda (v3.3a)[144] and RNAhybrid (v2.2.1)[145]. For miranda, we determined the optimal minimum free energy (MFE) cut-off by employing empirical data (real miRNA – target gene pairs predicted by miranda)[144] and plotting their distribution. As shown in Supplementary Figure 20, we observed a wide range of MFE values and chose -10 kcal/mol as the cut-off. For RNAhybrid, no empirical data is available, therefore we used the default cut-off of -20 kcal/mol. To increase the reliability of target prediction, we only kept target genes that were predicted by both methods or target genes that were predicted in multiple species ($n > 1$) by one method. To predict miR-337-3p targets in human, we extracted 3'UTR sequences from the same 13,083 genes in the human genome (hg38) that had corresponding 'pseudo' 3'UTRs found in 6 bats (the 'master' list). This allowed us to compare predicted targets in 3'UTRs of the same set of genes in bats and humans, which is a requirement to test whether the differences in the miR-337-3p seed region alters the set of predicted target genes. Targets of human miR-337-3p were predicted using the same procedure as described above.

GO enrichment analysis was performed using DAVID[146]. The non-redundant list of target genes predicted above was used as a query list while the list of the non-redundant genes that had 3'UTR data supported by Iso-seq was used as the background list. Enrichment analysis was performed on the first sublevel GO terms for Biological Process (BP), Cellular Component (CC) and Molecular Function (MF), using Fisher's exact tests. Enriched GO terms with a P-value < 0.05 after correcting for multiple testing using the Benjamini-Hochberg method were considered statistically significant.

5.4 Functional validation of novel miRNAs and their regulatory gene targets

Luciferase assays to test the functionality of miRNAs were designed and performed as described previously[147,148] in HEK293 cells. Cells were obtained from ATCC, authenticated by visual inspection of morphology and STR analysis, and free from mycoplasma contamination. The precursor sequences predicted by miRDeep2 were cloned in the pLKO.1 vector (Invitrogen) carrying the flanking sequences representing the primary transcript of the hsa-miR-342, which allowed optimal

transcription; we did this in order to ensure transcription from a known and reliably expressed pri-miRNA. All insertions were confirmed by Sanger sequencing. To maximize the sensitivity of the assay, we designed the miRNA sensors to contain two copies of the ideal targets of the to-be-tested miRNA. To this end, we inserted two repetitions of a fully complementary sequence to the cognate miRNA within the 3'UTR of the firefly luciferase gene in the pmiR-GLO vector (Promega). All cloning oligonucleotides are listed in Supplementary Table 14. Luciferase assays were performed in HEK293 cells as described in [147,148]. Briefly, 50ng of miRNA expression vector and 70ng of sensor vector were combined and transfected in HEK293 cells (18K cells per well in 96well plate format, density $5.625x10^5$ cells per cm^2) using GeneJuice (Merck Millipore) transfection reagent. 48 h post-transfection, firefly luciferase and renilla luciferase activities were measured as per manufacturer's instructions (Dual Luciferase reporter assay system, Promega), using a fully automated plate reader (TECAN, F200PRO or TECAN MPLEX, both equipped with fully automated injectors). Ratios between the firefly/renilla luciferase activity were normalized to account for technical variability. All box plots displaying reporter assay results (Fig. 4c-d, main text) extend from the 25th to 75th percentiles, the central line represents the median value, and whiskers are drawn using the function "min to max" in GraphPad Prism7 (GraphPad Software, La Jolla California USA, http://www.graphpad.com) and go down to the smallest value and up to the largest.

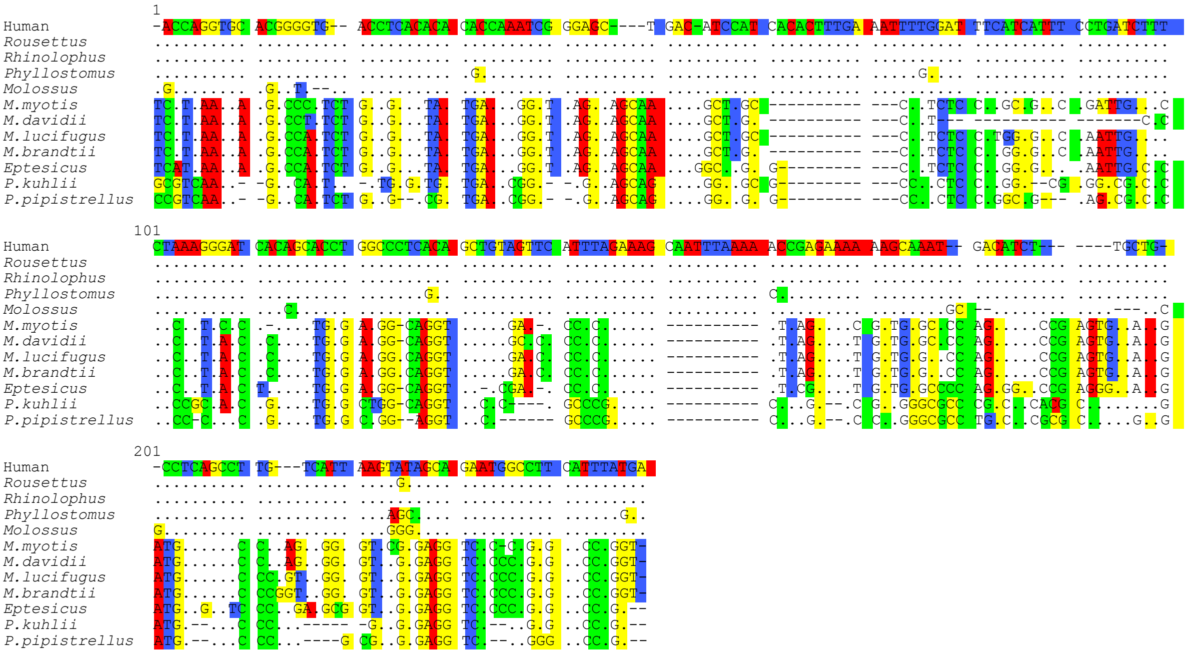

Supplementary Figure 1: **Alignment of bat UCE.47 sequences showing sequence divergence in *Myotis* and *Pipistrellus* bats**. Dots in the alignment represent nucleotides that are identical to the human sequence shown at the top. While *R. aegyptiacus*, *R. ferrumequinum*, *P. discolor*, and *M. molossus* have few sequence changes compared to human, *M. myotis* and *P. kuhlii* show numerous mutations. Importantly, many of these mutations are shared between *M. myotis* and *P. kuhlii*, indicating that these mutations already arose early in Vespertilionid lineage. Supporting this, mutations are also shared with related Vespertilionid species. Shared mutations also show that the sequence divergence is real and not attributed to base errors in the assemblies.

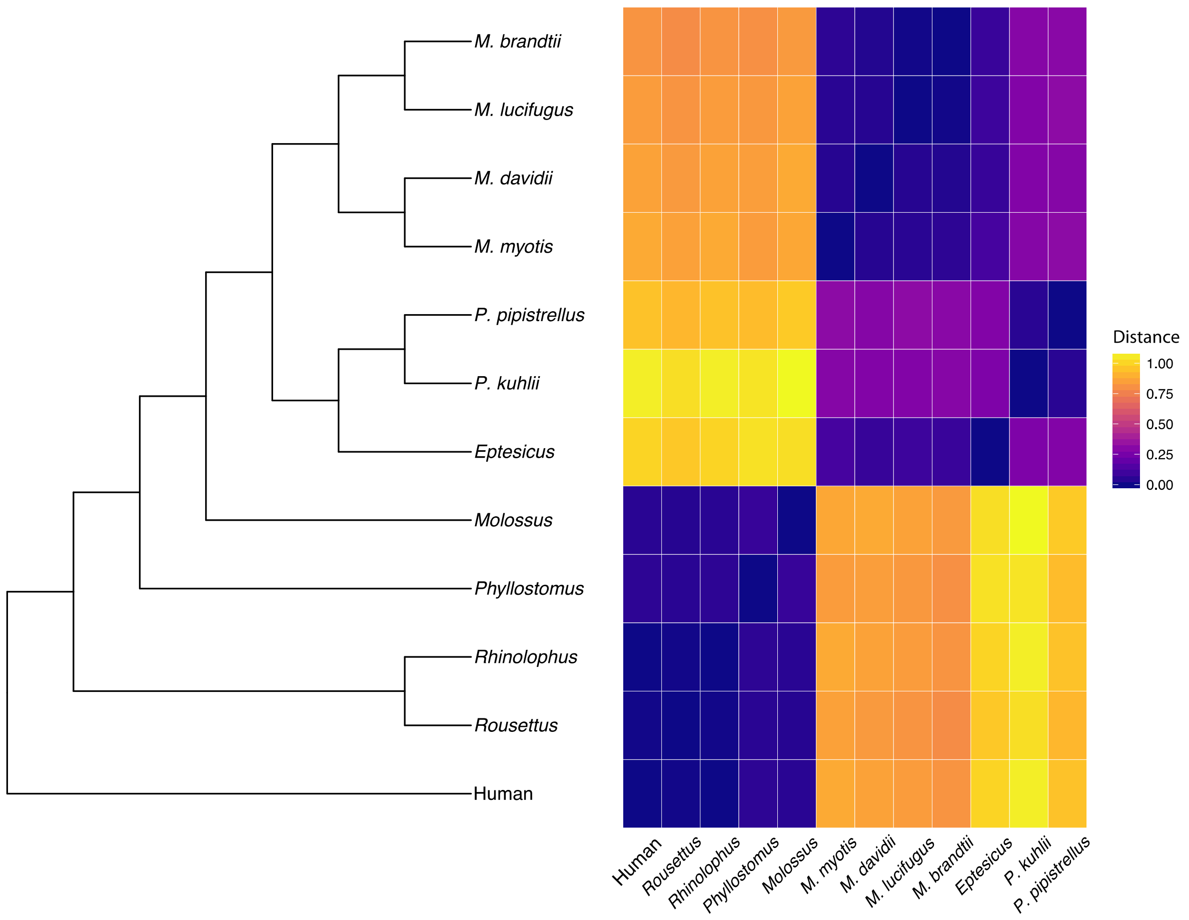

Supplementary Figure 2: Genetic distance of Vespertilionid UCE.47 to other bats and canonical human sequence. The heatmap shows that the UCE.47 sequences of *Rousettus*, *Rhinolophus*, *Phyllostomus*, *Molossus* and human are highly conserved. In contrast, the sequence of Vespertilionid bats, represented by *Myotis*, *Eptesicus* and *Pipistrellus* species, is substantially diverged from the conserved UCE sequence but relatively similar within the clade.

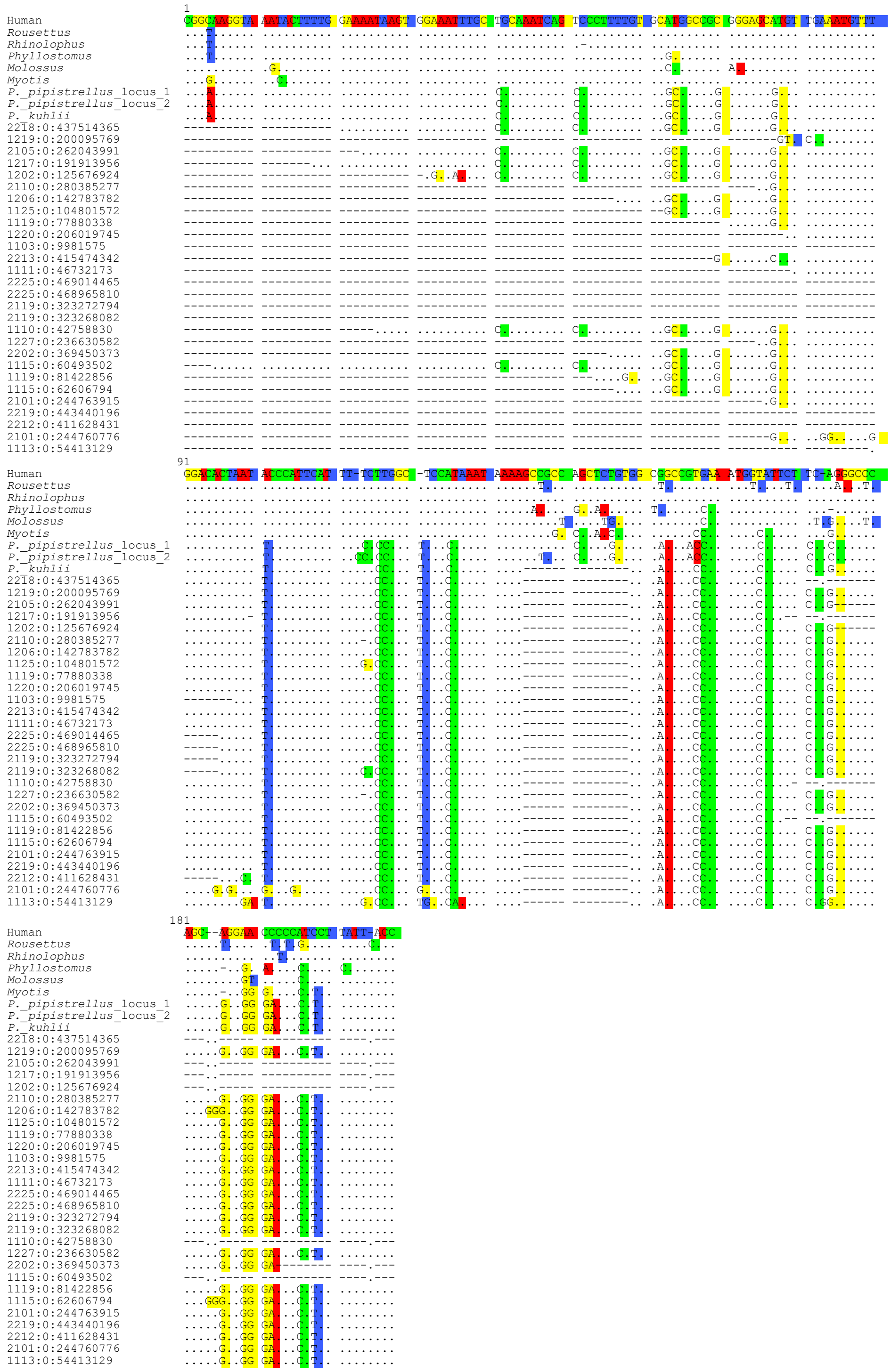

Supplementary Figure 3: Alignment of bat UCE.394 sequences showing sequence divergence in *Pipistrellus* bats. Dots in the alignment represent nucleotides that are identical to the human sequence shown at the top. Compared to other bats, *P. kuhlii* shows an increased number of mutations in this UCE sequence. These mutations are supported by Illumina reads (note that reads do not cover the entire UCE locus). Furthermore, most mutations are shared with *P. pipistrellus* (which has two nearly identical loci in the current genome assembly), indicating that the sequence divergence is real and not attributed to base errors in the assembly.

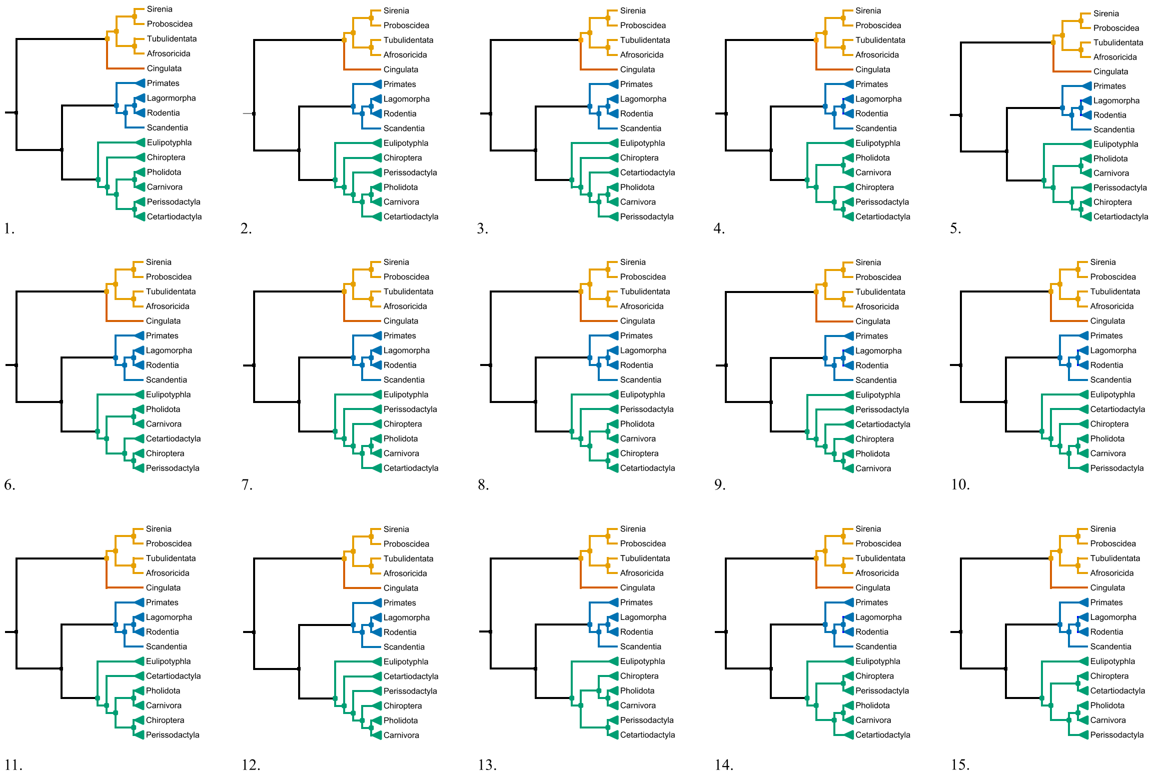

Supplementary Figure 4: **The 15 topologies showing different arrangements of Laurasiatheria.**

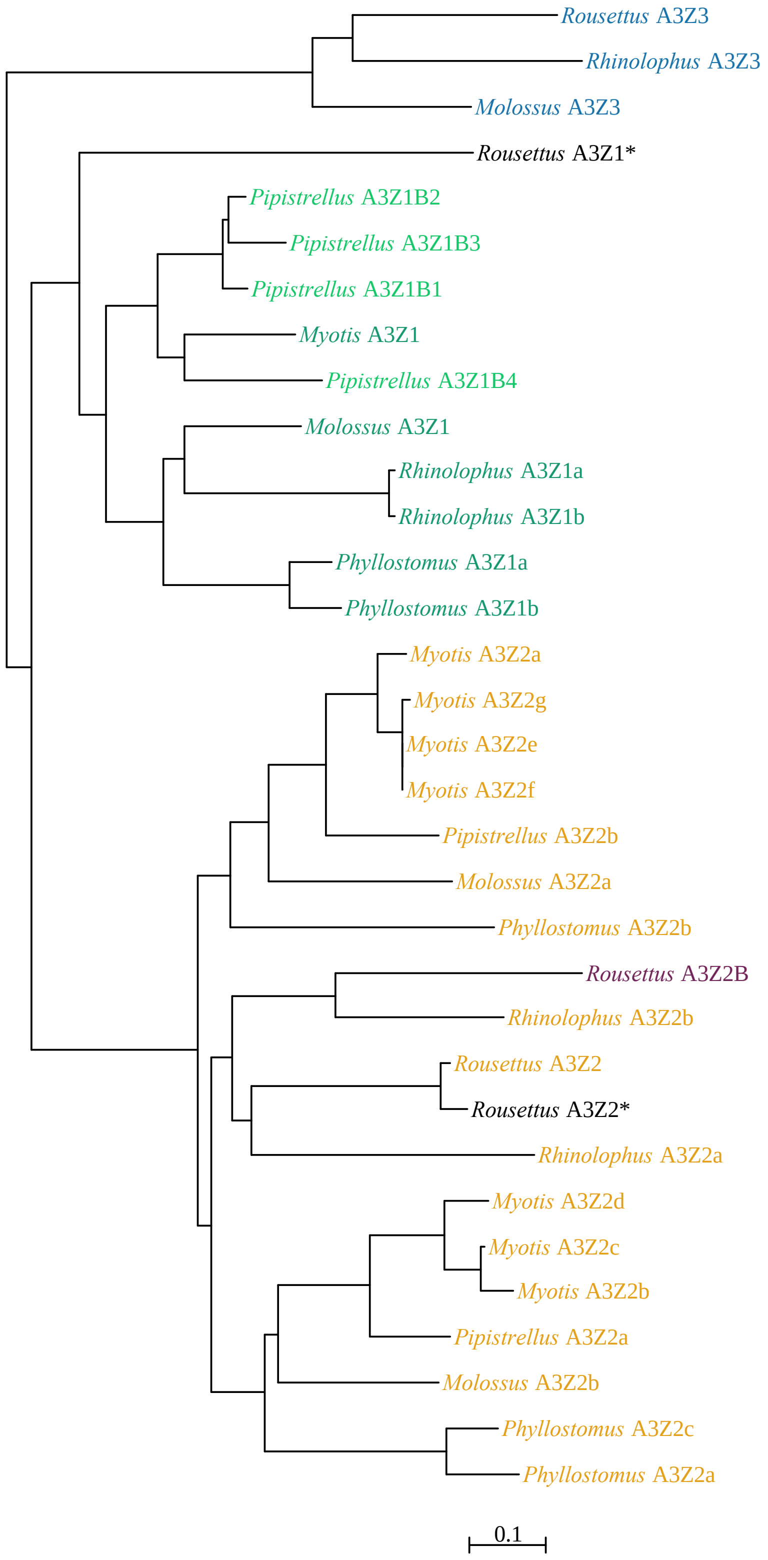

Supplementary Figure 5: Gene tree showing the evolution of *APOBEC3* genes in mammals. A phylogenetic tree was reconstructed using BioNJ. We used the Z domain of the members of the panther gene family PTHR13857, added by *APOBEC3* genes we annotated in our six bat genomes. In addition to a possible small expansion in the ancestral bat lineage, the tree supports a scenario of several additional *APOBEC3* expansions in independent bat lineages.

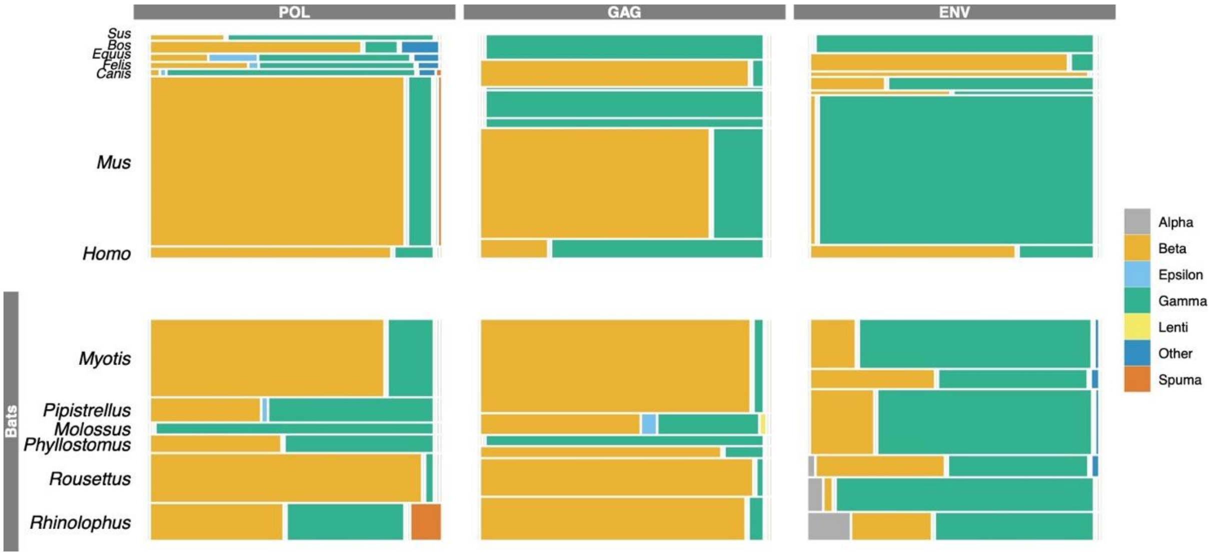

Supplementary Figure 6: Mosaic plot showing the relative numbers of viral gene sequences found in six bat genomes compared to seven mammalian reference genomes.

Supplementary Figure 7: Reconstructed phylogenetic tree of viral pol sequences found in six species of bats. Each species used in the ERV search is marked with a colour: *Phyllostomus* (navy blue), *Myotis* (green), *Pipistrellus* (orange), *Rhinolophus* (yellow), *Molossus* (light blue), *Rousettus* (pink) and reference sequences (black). Bootstrap values are shown where the values are ≥ 70%. The tips of the phylogeny are labelled with the species name, position in the reference genomes and the direction of the sequences (N- negative/P- positive).

(See Separate File for Supplementary Figure 7)

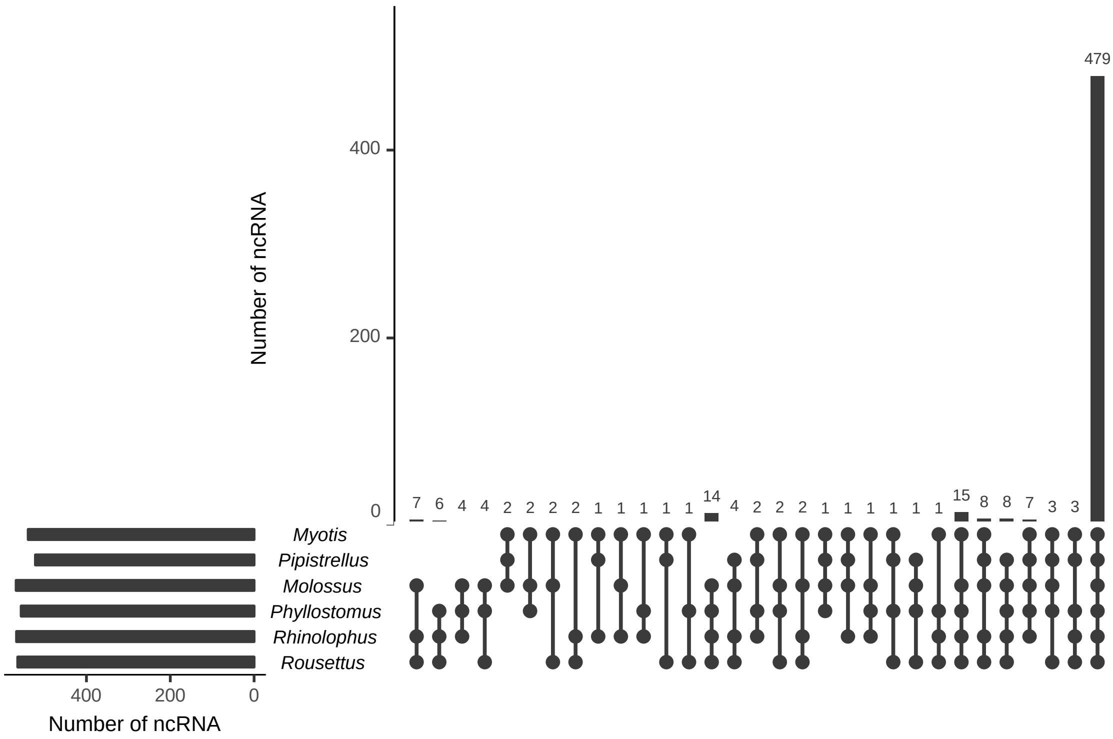

Supplementary Figure 8: The number of conserved noncoding RNA genes shared across 6 bat species. The figure was generated using UpSetR[149]. Each black dot indicates each species. The connected black dots indicate the number of noncoding RNA genes shared between them.

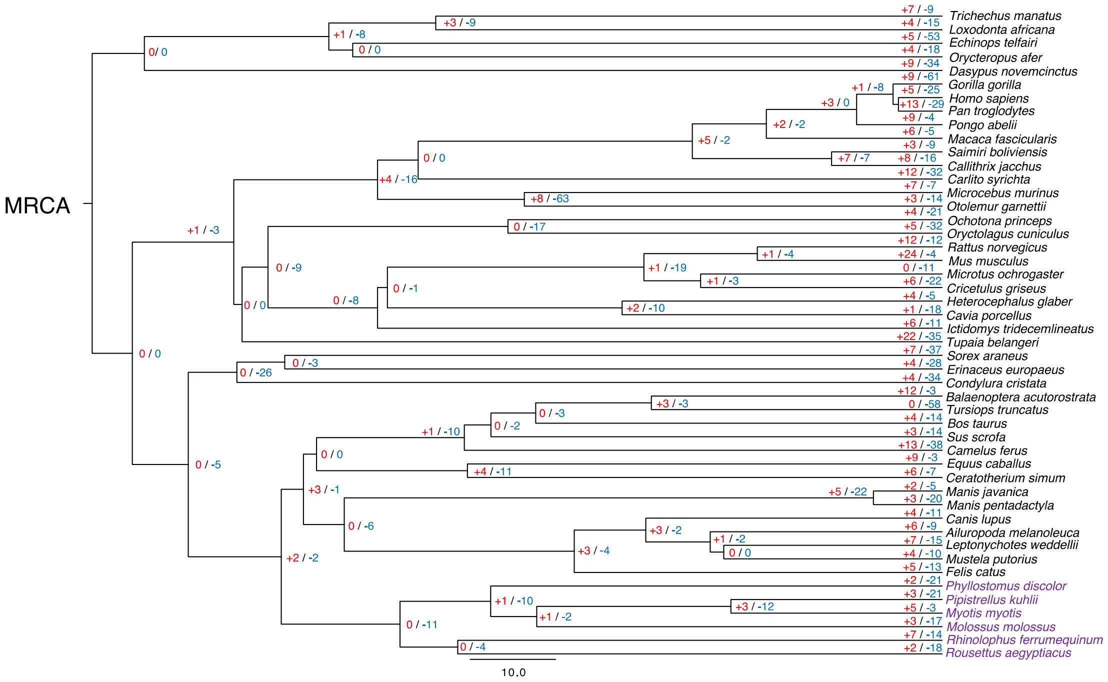

Supplementary Figure 9: The number of miRNA gene families under contraction and expansion along the lineages. The phylogenetic tree was inferred based on the alignment of 12,931 protein-coding genes. Based on 286 conserved miRNA gene families, the number of miRNA families under a significant rate of contraction and expansion was inferred by CAFE. The red values indicate the number of expanded miRNA families while the blue values indicate the number of contracted miRNA families. The bat species were highlighted in purple. MRCA is the acronym of Most Recent Common Ancestor.

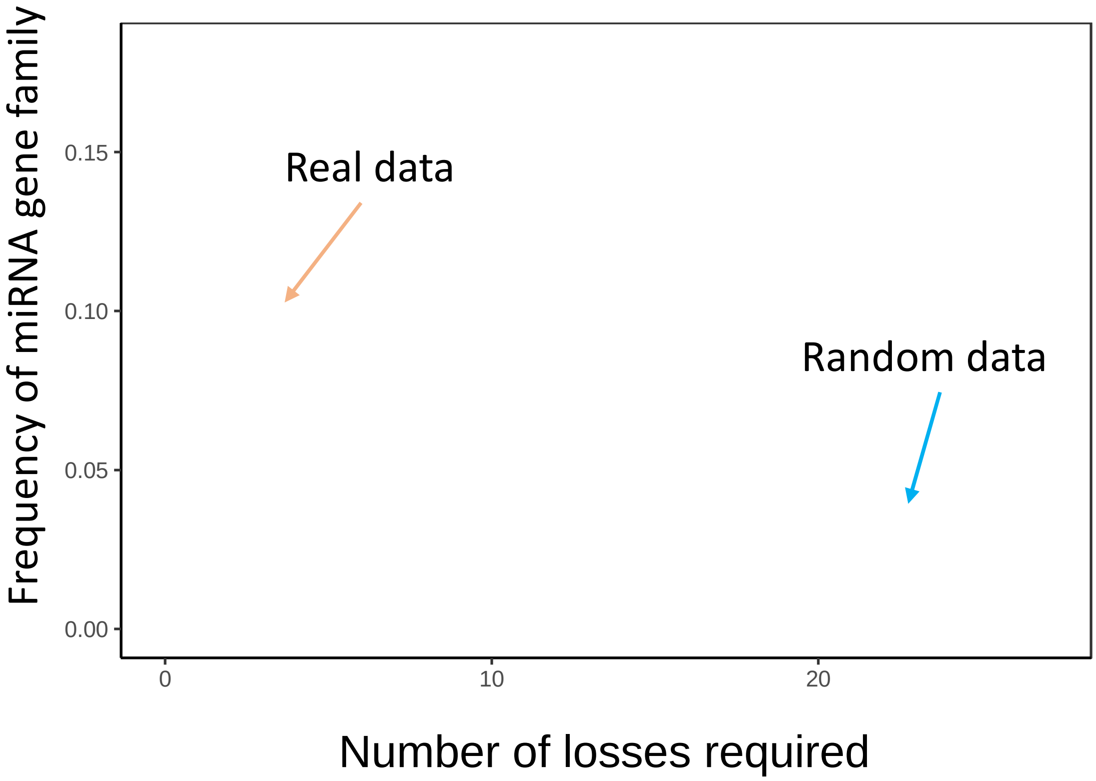

Supplementary Figure 10: The performance of the Dollo parsimony principle on random and real data. The real data refer to the observed phylogenetic tree inferred from the alignment of 12,931 protein-coding genes and the matrix containing the number of miRNA copies across 48 mammalian species; while the random data refer to the relationship of species in the phylogenetic tree and the number of miRNA copies that have been randomly shuffled. The figure indicates the number of losses required to explain the observed phylogenetic pattern, both on real and random data.

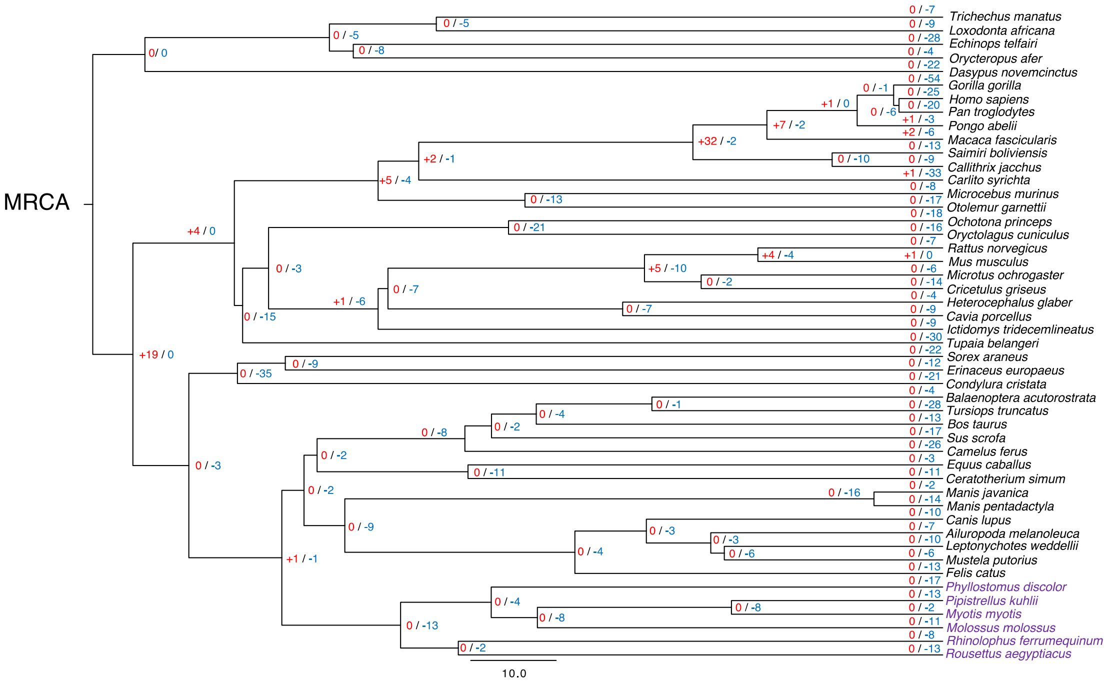

Supplementary Figure 11: The miRNA gene loss and acquisition based on the Dollo parsimony principle. The red numbers indicate miRNA gain while the blue numbers indicate miRNA loss. The bat species were highlighted in purple.

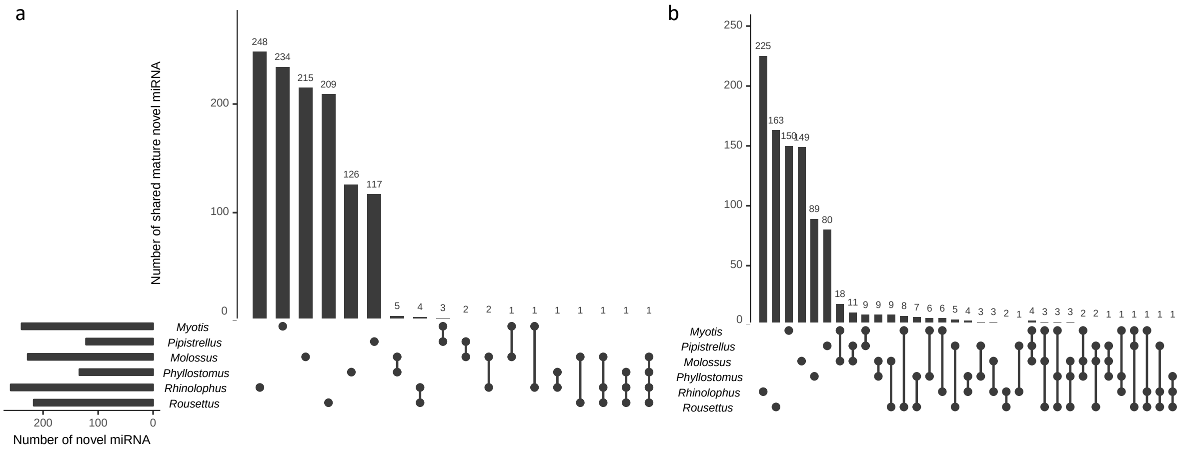

Supplementary Figure 12: The evolution of novel miRNA predicted in 6 bat species. a) The number of novel mature miRNA shared across 6 bat species. **b)** The number of novel seeds shared across 6 bat species.

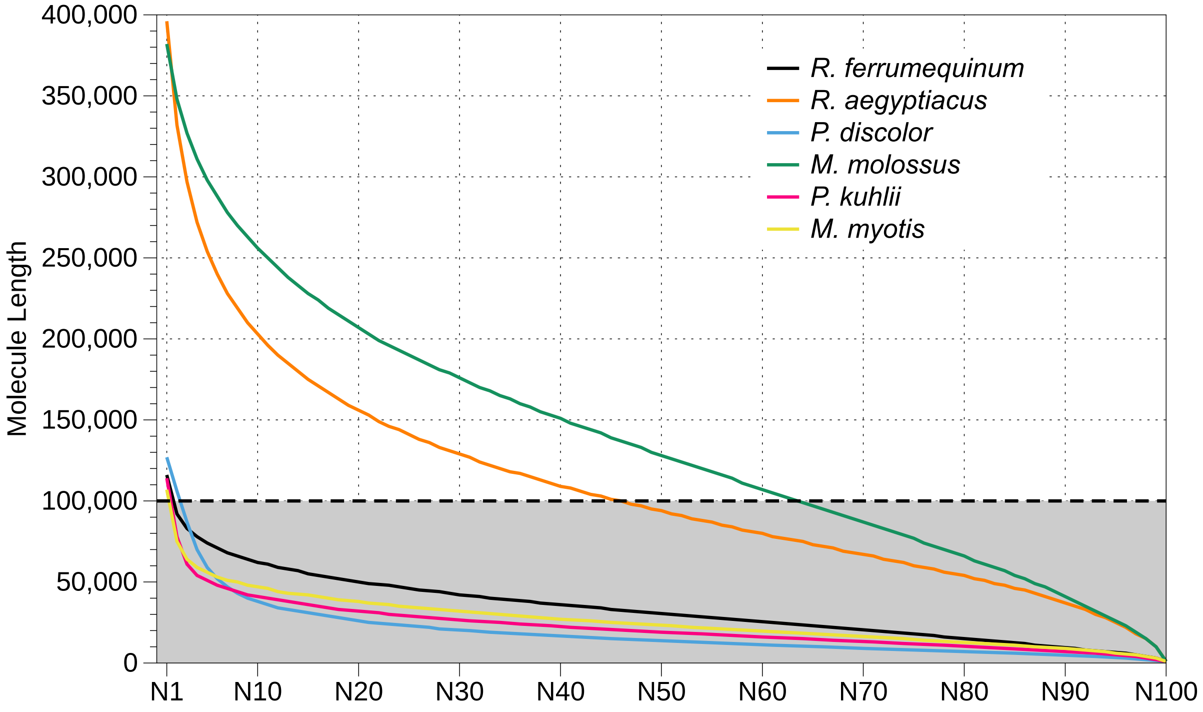

Supplementary Figure 13: Distribution of putative 10x molecule lengths (N1 to N100). Based on the final assemblies, 10x reads were mapped with longranger align and the molecule lengths were calculated using the tool bxcheck (https://github.com/pd3/bxcheck).

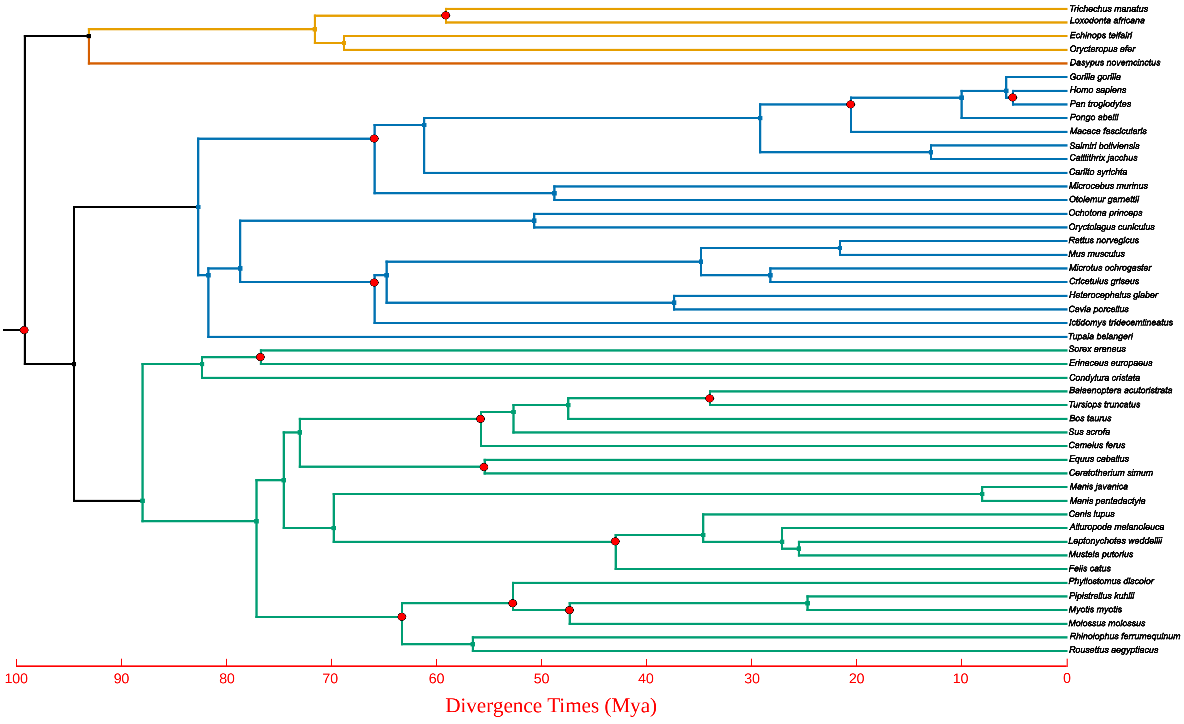

Supplementary Figure 14: The tree topology inferred with a supermatrix of 12,931 genes. Divergence times are calculated using the tree topology inferred with a supermatrix of 12,931 genes. Node calibrated with fossils are highlighted in red.

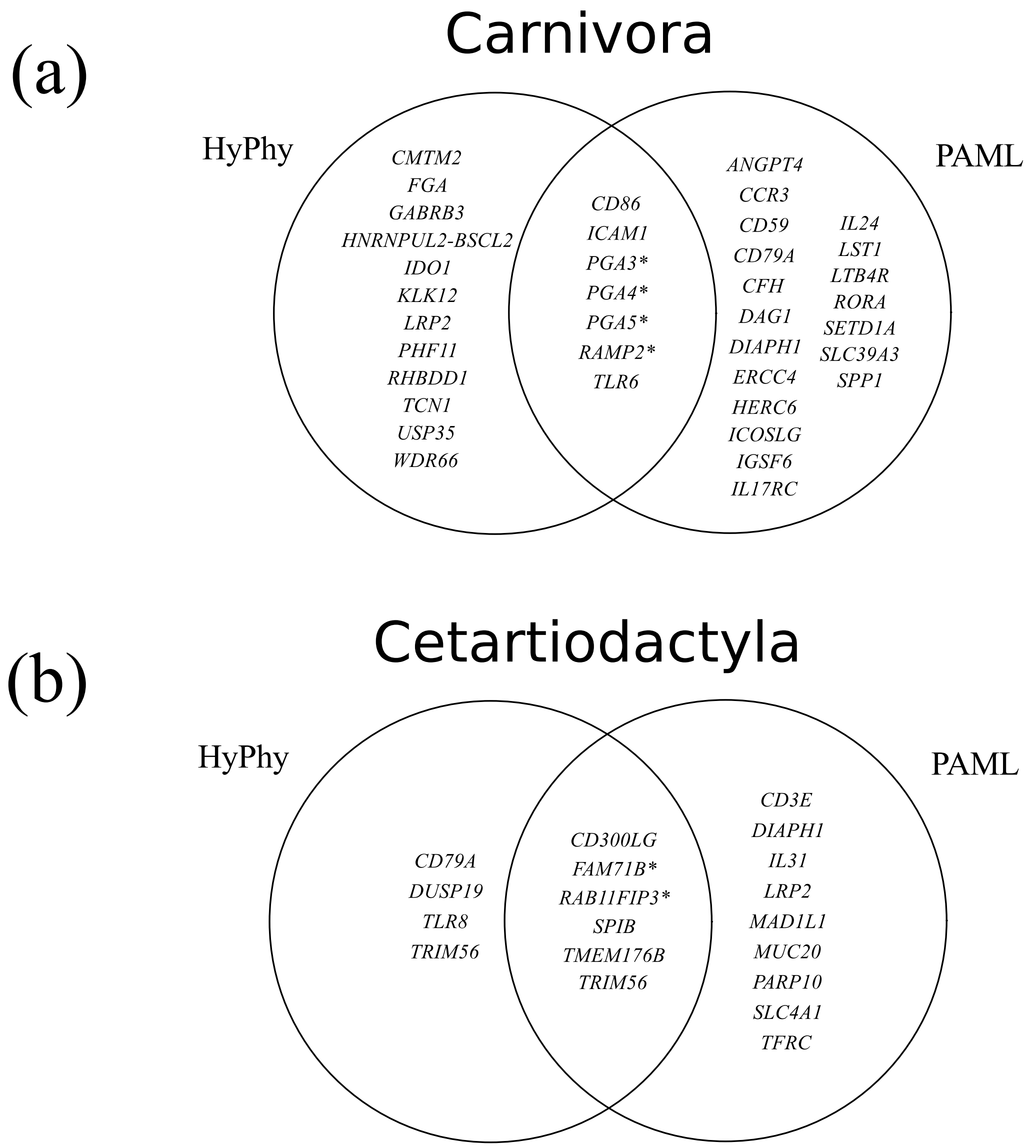

Supplementary Figure 15: Genes under positive selection on Carnivora and Cetartiodactyla branches (a) Genes showing evidence of significant positive selection after multiple test correction across both aBSREL (12,821 genes) and codeml (2,436 genes) for Carnivora are shown. Genes not in the candidate 2,453 genes that were significant in HyPhy and subsequently validated with codeml are designated by ‘*’. **(b)** Genes showing evidence of significant positive selection across both aBSREL (12,866 genes) and codeml (2,443 genes) for Cetartiodactyla are displayed.

(a)

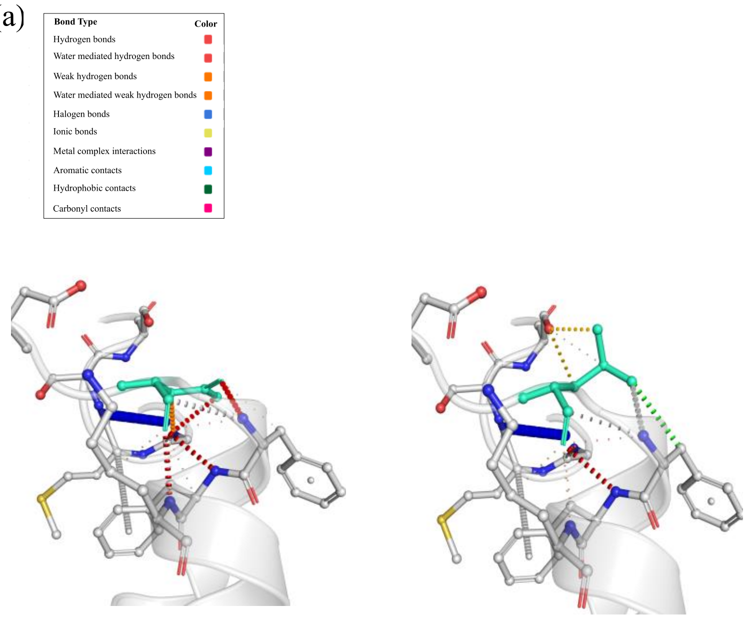

Bat wild type (Asparagine) Human variant (Leucine)

(b)

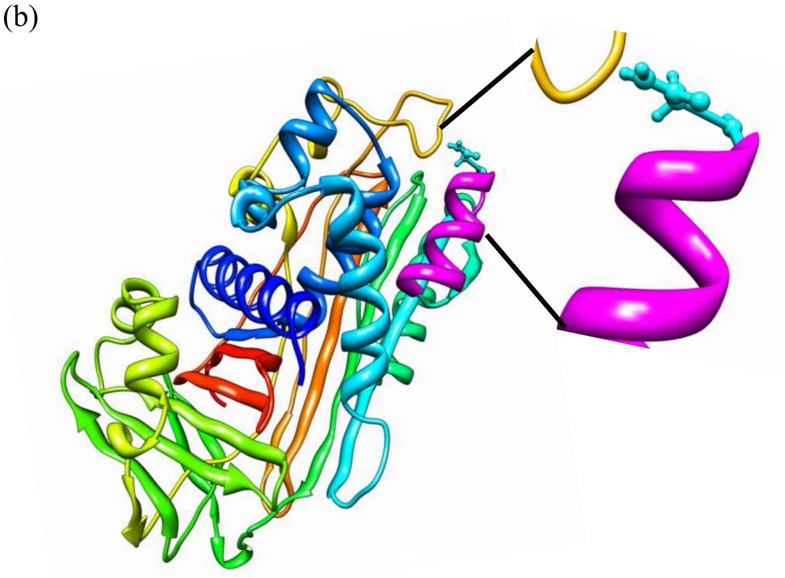

Supplementary Figure 16: SERPINB6 protein structure. (a) The protein site under selection in bats was explored for SERPINB6. To explore the effect of the substitution on the protein stability, the bat 'wild-type' was replaced with the 'human' version, with the net change in free energy determined. The bat-specific asparagine results in an increase in hydrogen bonds relative to the human leucine. **(b)** The inferred 3D structure for *SERPINB6* was determined using template-based modelling. The residue under selection, amino acid 108 is highlighted.

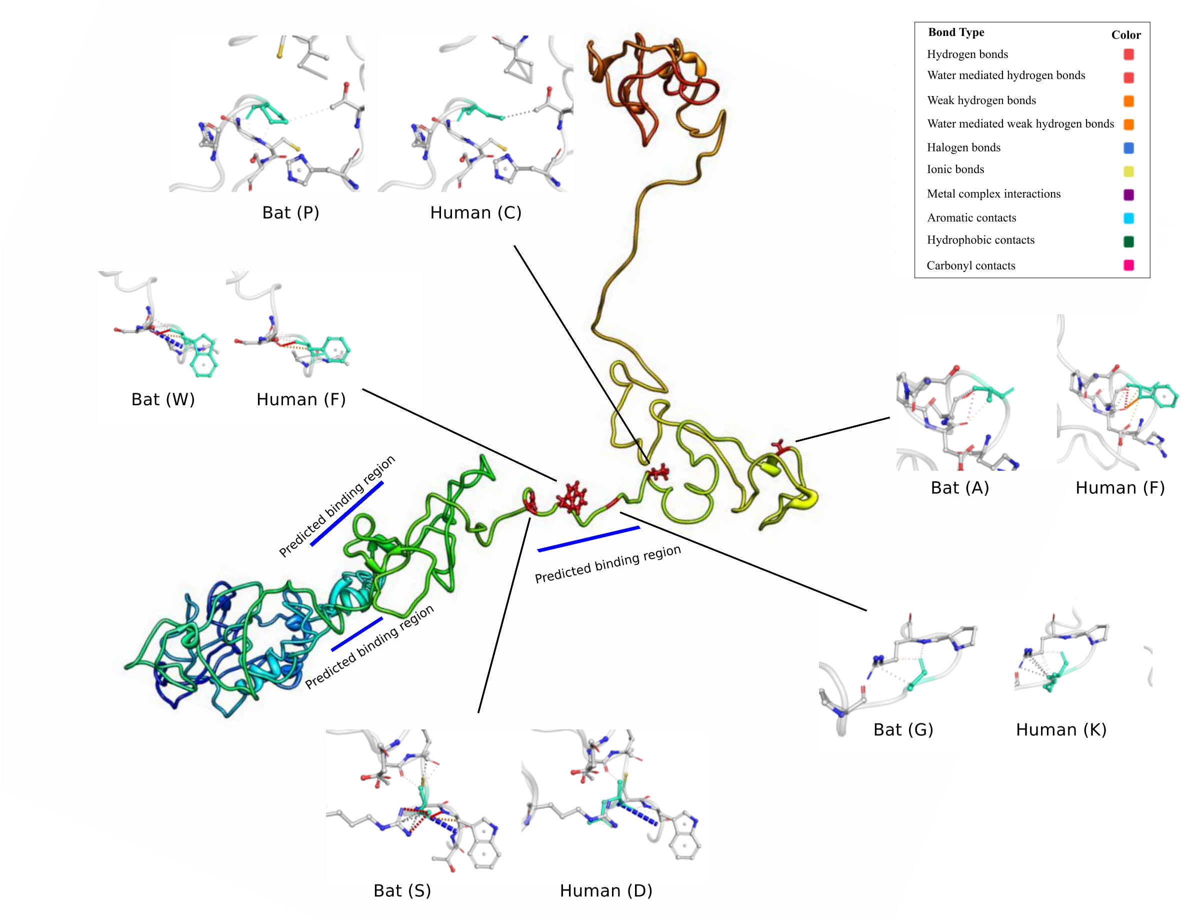

Supplementary Figure 17: INAVA protein structure. The inferred 3D structure for *INAVA* in *Rhinolophus ferrumequinum* was determined using template-based modelling. Predicted protein binding sites are highlighted. Five sites showing evidence of positive selection are also displayed. The bat-specific and human residues for each site, including their atomic bonds, are highlighted. The inferred *INAVA* structure contains a large number of coils and loops.

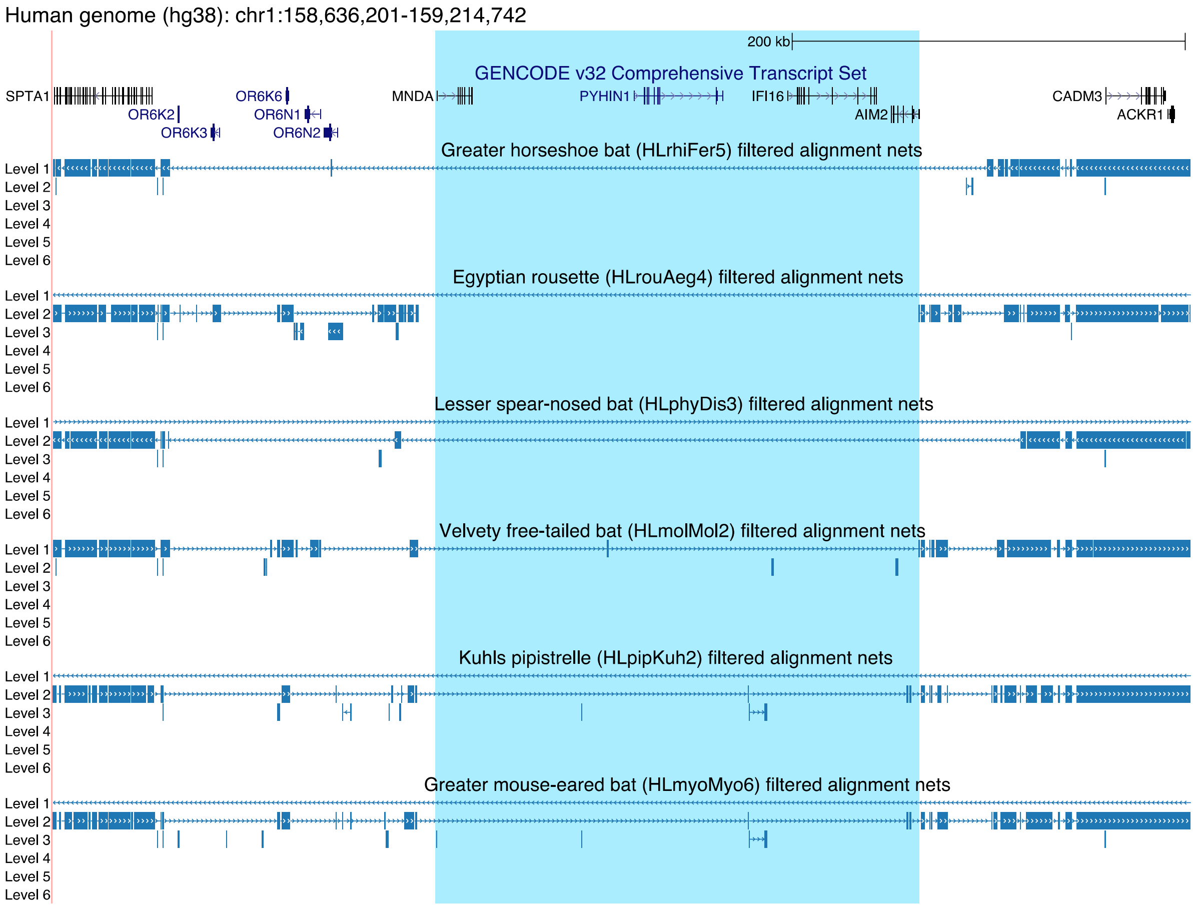

Supplementary Figure 18: Loss of *PYHIN* genes in bats. UCSC genome browser screenshot shows the *PYHIN* genes in the human genome (blue highlight). The alignment nets show that all six bats have large deletions that remove these genes, which is consistent with previous results that all bats have lost these genes[130]. Because these genes are also lost in several other mammals, we filtered them out in our genome-wide screen for gene losses.

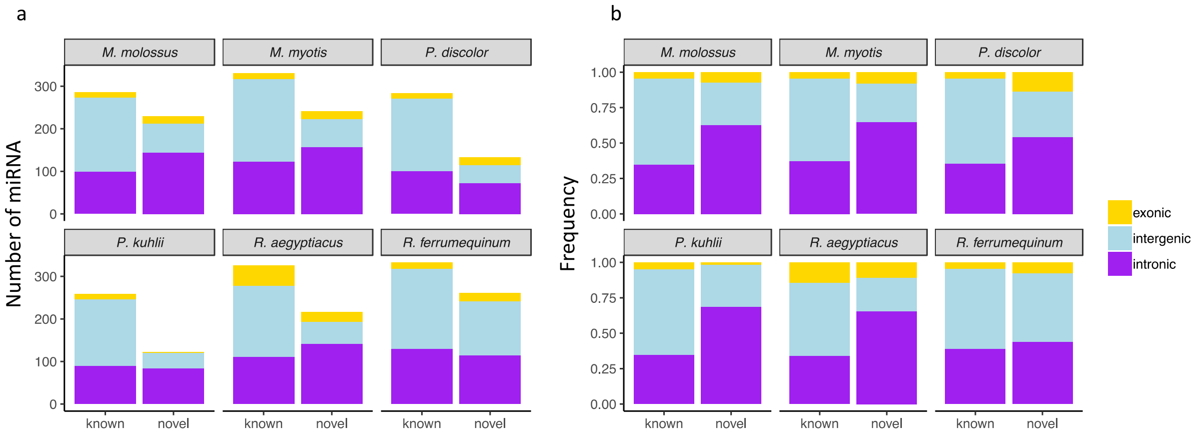

Supplementary Figure 19: Genomic annotation of predicted miRNA loci in 6 bat species. a) The number of known and novel miRNA located in exonic, intronic and intergenic regions; **b)** The percentage of known and novel miRNA located in exonic, intronic and intergenic regions.

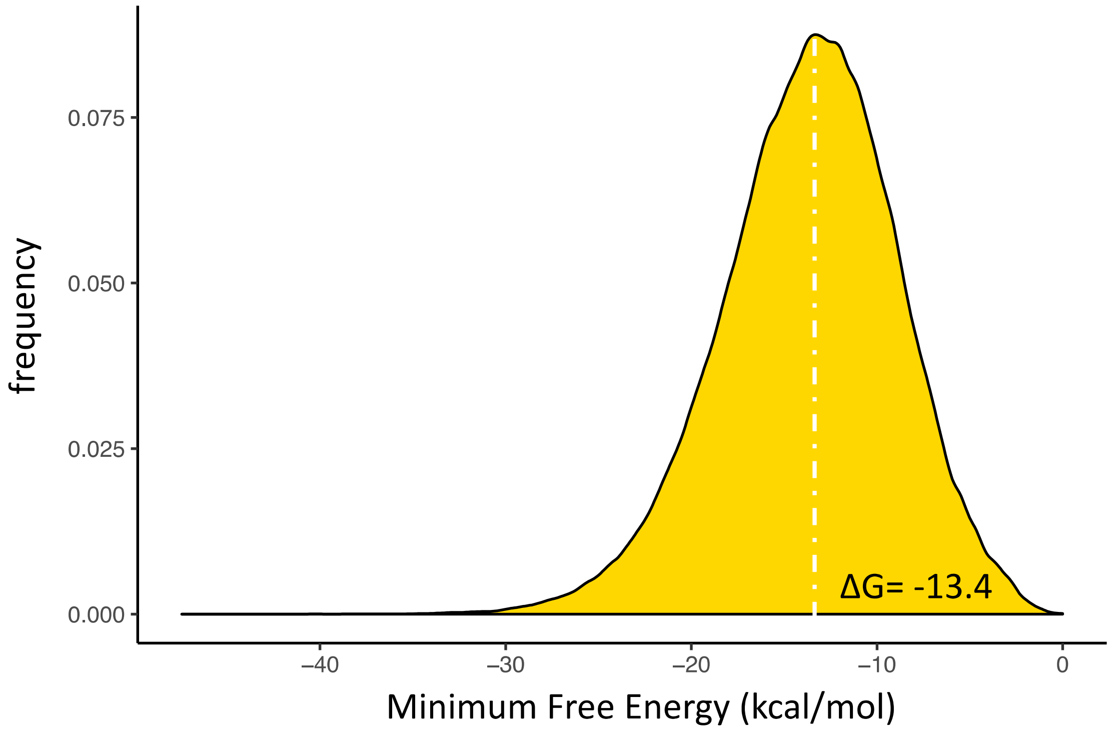

Supplementary Figure 20: The empirical minimum free energy (MFE) distribution based on 1,000,000 human miRNA and target predictions by miranda.

Supplementary Table 1: Information of 48 mammalian genomes used for comparative and phylogenetic analyses in this study. The species highlighted in blue indicate 6 bat species whose genomes were sequenced in this study while the species in red indicate 7 species used as references for comparative analyses. For cow and cat, we additionally used the most recent bosTau9 and felCat9 assembly to assess completeness of BUSCO genes and UCEs.

Species name	Common name	Order	Genome version
Trichechus manatus	American manatee	Afrotheria	triMan1
Loxodonta africana	African bush elephant	Afrotheria	loxAfr3
Orycteropus afer	Aardvark	Afrotheria	oryAfe1
Echinops telfairi	Lesser hedgehog	Afrotheria	echTel2
Dasypus novemcinctus	Armardillo	Xenarthra	dasNov3
Gorilla gorilla	Gorilla	Primates	gorGor5
Homo sapiens	Human	Primates	hg38
Pan troglodytes	Chimpanzee	Primates	panTro5
Pongo abelii	Orangutan	Primates	ponAbe2
Macaca fascicularis	Long-tailed macaque	Primates	macFas5
Saimiri boliviensis	Squirrel monkey	Primates	saiBol1
Callithrix jacchus	Common marmoset	Primates	calJac3
Carlito syrichta	Tarsier	Primates	tarSyr2
Microcebus murinus	Grey mouse lemur	Primates	micMur3
Otolemur garnettii	Northern greater galago	Primates	otoGar3
Ochotona princeps	American pika	Largomorpha	ochPri3
Oryctolagus cuniculus	European rabbit	Largomorpha	oryCun2
Rattus norvegius	Rat	Rodentia	Rnor6
Mus musculus	Mouse	Rodentia	mm10
Microtus ochrogaster	Vole	Rodentia	micOch1
Cricetulus griseus	Chinese hamster	Rodentia	criGri1
Heterocephalus glaber	Naked mole rat	Rodentia	hetGla2
Cavia porcellus	Guinea pig	Rodentia	cavPor3
Ictidomys tridecemlineatus	Thirteen-lined ground squirrel	Rodentia	speTri2
Tupaia belangeri	Northern tree shrew	Scandentia	tupBel1
Sorex araneus	Common shrew	Eulipothphla	sorAra2
Erinaceus europaeus	European hedgehog	Eulipothphla	eriEur2
Condylura cristata	Star-nosed mole	Eulipothphla	conCri1
Balaenoptera acutorostrata	Minke whale	Cetartiodactyla	balAcu1
Tursiops truncatus	Bottlenose dolphin	Cetartiodactyla	turTru3
Bos taurus	Cattle	Cetartiodactyla	bosTau8
Sus scrofa	Pig	Cetartiodactyla	susScr11
Camelus ferus	Camel	Cetartiodactyla	camFer2
Equus caballus	Horse	Perissodactyla	equCab3
Ceratotherium simum	White rhinoceros	Perissodactyla	cerSim1
Manis javanica	Sunda pangolin	Pholidota	manJav1
Manis pentadactyla	Chinese pangolin	Pholidota	manPen1
Canis lupus familiaris	Dog	Carnivora	canFam3
Ailuropoda melanoleuca	Giant panda	Carnivora	ailMel1
Leptonychotes weddellii	Seal	Carnivora	lepWed1
Mustela putorius	Polecat	Carnivora	musPut1
Felis catus	Cat	Carnivora	felCat8
Phyllostomus discolour	Pale spear-nosed bat	Chiroptera	phyDis3
Pipistrellus kuhlii	Kuhl's pipistrelle	Chiroptera	pipKuh2
Myotis myotis	Greater mouse-eared bat	Chiroptera	myoMyo6
Molossus molossus	Velvety free-tailed bat	Chiroptera	molMol2
Rhinolophus ferrumequinum	Greater horseshoe bat	Chiroptera	rhiFer5
Rousettus aegyptiacus	Egyptian fruit bat	Chiroptera	rouAeg4

Supplementary Table 2: The final genomes (all lengths in Mb). N50 values correspond to *post hoc* genome size of the final assemblies (sum of the length of all scaffolds).

<table>
<tr><th rowspan="2">Species</th><th colspan="4">Scaffolds</th><th colspan="5">Primary Contigs</th></tr>
<tr><th>Number</th><th>Total Length</th><th>Maximum Length</th><th>N50</th><th>Number</th><th>Total Length</th><th>Maximum Length</th><th>N50</th><th>Avg. QV</th></tr>
<tr><td>M. myotis</td><td>92</td><td>2,003</td><td>223</td><td>94.45</td><td>630</td><td>1,974</td><td>52</td><td>12.51</td><td>42.7</td></tr>
<tr><td>P. kuhlii (pre-curation)</td><td>202</td><td>1,776</td><td>197</td><td>80.24</td><td rowspan="2">597</td><td rowspan="2">1,763</td><td rowspan="2">51</td><td rowspan="2">10.59</td><td rowspan="2">40.8</td></tr>
<tr><td>P. kuhlii (post-curation)</td><td>129</td><td>1,776</td><td>206</td><td>96.73</td></tr>
<tr><td>R. ferrumequinum</td><td>50</td><td>2,075</td><td>126</td><td>92.00</td><td>347</td><td>2,056</td><td>81</td><td>21.75</td><td>46.2</td></tr>
<tr><td>P. discolor</td><td>41</td><td>2,095</td><td>215</td><td>171.08</td><td>451</td><td>2,059</td><td>72</td><td>15.51</td><td>42.9</td></tr>
<tr><td>R. aegyptiacus</td><td>29</td><td>1,894</td><td>186</td><td>113.81</td><td>271</td><td>1,867</td><td>81</td><td>22.00</td><td>43.9</td></tr>
<tr><td>M. molossus</td><td>60</td><td>2,319</td><td>252</td><td>110.67</td><td>412</td><td>2,268</td><td>77</td><td>22.17</td><td>42.2</td></tr>
</table>

Supplementary Table 3: Manual curation and consistency with karyotypes. This table shows data for both the non-curated and curated *P. kuhlii* assembly.

Species	Splits / Joins	# of Chromosomes in Karyotype = N	Correlation with top N Scaffolds	Tail Status	% of Data in N Largest Scaffolds
M. myotis	2 / 6	21+2	0.993	Tail (18)	95.62%
P. kuhlii	not curated	21+2	0.992	Tail (33)	86.54%
P. kuhlii	4 / 76	21+2	0.995	Tail (2)	97.99%
R. ferrumequinum	3 / 1	28+1	0.980	Incline (5)	98.89%
P. discolor	10 / 13	15+2	0.989	Cliff (0)	99.67%
R. aegyptiacus	0 / 5	17+2	0.995	Cliff (0)	99.85%
M. molossus	2 / 6	23+2	0.989	Incline (7)	98.52%

Supplementary Table 4: The presence of highly-conserved BUSCO genes in the genome and in the gene annotations.

			BUSCO applied to genome assembly			BUSCO applied to gene annotation		
	**species**	**assembly**	**Complete**	**Fragmented**	**Missing**	**Complete**	**Fragmented**	**Missing**
	Homo	hg38 (GCA_000001405.27)	94.59%	2.46%	2.95%	99.95%	0.00%	0.05%
	Mus	mm10 (GCA_000001635)	95.30%	2.36%	2.34%	99.83%	0.02%	0.15%
	Canis	canFam3 (GCA_000002285.2)	95.30%	2.36%	2.34%	98.56%	1.00%	0.44%
	Felis	felCat9 (GCA_000181335.4)	95.30%	2.39%	2.31%	98.39%	0.97%	0.63%
	Equus	equCab3 (GCF_002863925.1)	96.22%	2.07%	1.71%	97.78%	0.85%	1.36%
	Bos	bosTau9 (GCA_002263795.2)	94.10%	2.97%	2.92%	98.98%	0.68%	0.34%
	Sus	susScr11 (GCF_000003025.6)	94.08%	3.51%	2.41%	98.85%	0.68%	0.46%
Bat1K assemblies	*Rhinolophus*	this study	95.42%	2.36%	2.22%	99.66%	0.17%	0.17%
	Rousettus	this study	95.83%	1.92%	2.24%	99.34%	0.29%	0.37%
	Phyllostomus	this study	94.91%	1.80%	3.29%	99.66%	0.15%	0.19%
	Molossus	this study	92.93%	3.17%	3.90%	99.49%	0.19%	0.32%
	Pipistrellus	this study	95.35%	2.36%	2.29%	99.56%	0.17%	0.27%
	Myotis	this study	94.44%	2.88%	2.68%	99.63%	0.24%	0.12%

Supplementary Table 5: Analysis of ultraconserved elements (UCEs) that do not align with ≥ 85% identity and at least 150 bp.

UCE not found	species in which the UCE was not found	assembly	Reason	Reason
uc.157	Miniopterus	minNat1	Assembly artifact	UCE overlaps a 1189 bp assembly gap
uc.158	Miniopterus	minNat1	Assembly artifact	UCE overlaps a 1833 bp assembly gap
uc.159	Miniopterus	minNat1	Assembly artifact	UCE overlaps a 1786 bp assembly gap
uc.157	Cow	bosTau8	Assembly artifact	part of the UCE overlap a 193 bp assembly gap. UCE is fully present in the new bosTau9 assembly.
uc.170	Cow	bosTau8	Assembly artifact	UCE overlaps a 100 bp assembly gap. UCE is fully present in the new bosTau9 assembly.
uc.10	Dog	canFam3	Assembly artifact	UCE overlaps a 300 bp assembly gap contained in a 4.3 kb locus in canFam3. However gap size is likely underestimated as the corresponding locus in human is 100 kb and several chrUn parts align to the human locus (but not the UCE). UCE is fully present in the Dingo (Canis lupus dingo) assembly
uc.157	Dog	canFam3	Assembly artifact	UCE overlaps a 476 bp region in canFam2 (chr3:31,944,063-31,944,538) with low quality scores (Phred <30), this low-quality region has remained identical in canFam3. Searching the NCBI traces finds a single Sanger Read (ti:294708094) that aligns well but was apparently not incorporated in the assembly. UCE is fully present in the Dingo (Canis lupus dingo) assembly
uc.296	Dog	canFam3	Assembly artifact	UCE overlaps a 340 bp assembly gap. UCE is fully present in the Dingo (Canis lupus dingo) assembly
uc.3	Dog	canFam3	Assembly artifact	UCE overlaps a 4474 bp assembly gap. UCE is fully present in the Dingo (Canis lupus dingo) assembly
uc.157	Cat	felCat8	Assembly artifact	UCE overlaps a 643 bp assembly gap. UCE is fully present in the new felCat9 assembly.
uc.398	Cat	felCat8	Assembly artifact	UCE overlaps a 1270 bp assembly gap. UCE is fully present in the new felCat9 assembly.
uc.47	Myotis	this study	real divergence	
uc.394	Pipistrellus	this study	real divergence	
uc.446	Pipistrellus	this study	real divergence	
uc.47	Pipistrellus	this study	real divergence	

Supplementary Table 6: Best-fit models of sequence evolution for all coding genes, CNEs and 1st +2nd codon site alignments were determined using IQTREE.
(See separate file)

Supplementary Table 7: The metrics of the alignments of 12,931 genes.
(See separate file)

Supplementary Table 8: Results of the genome-wide screen for positive selection in coding genes. Uncorrected P-values are calculated by using the likelihood ratio test as implemented for the aBSREL model in HyPhy. These values are corrected using the Holm-Bonferroni correction for the number of branches tested. The Gene-corrected P-values are produced by applying the false discovery rate procedure over all genes tested (n=12,931) to the LRT derived p-value. The "Double-corrected" value is the Branch-corrected p-value post FDR correction (n=12,931).
(See separate file)

Supplementary Table 9: The significant genes under positive selection along the bat ancestral branch using PAML. The likelihood ratio test (LRT) was used to calculate p-values using a chi-square distribution (one sided). P-values were adjusted using FDR correction (n=2,453).
(See separate file)

Supplementary Table 10: The lists of genes under positive selection on Carnivora and Cetartiodactyla branches. The likelihood ratio tests (LRT) was used to calculate p-values using a chi-square distribution (one sided). P-values were adjusted using FDR correction, n=2,436 and 2,443, respectively.
(See separate file)

Supplementary Table 11: Genes that were inferred to be lost in all 6 bats analysed in this study.

Gene Symbol	Ensembl Gene ID
AS3MT	ENSG00000214435
HIST1H4K	ENSG00000273542
IL36G	ENSG00000136688
KLK4	ENSG00000167749
KRBA2	ENSG00000184619
LRRC70	ENSG00000186105
MS4A3	ENSG00000149516
U2AF1L4	ENSG00000161265
ZBED9	ENSG00000232040
ZFP30	ENSG00000120784

Supplementary Table 12: Gene families that were estimated to have undergone a contraction or expansion in the ancestral bat lineage. Values shown are P-values calculated using the Viterbi method as implemented in CAFE for evidence in shift in the rate of birth/death of a gene family along the given branch. "Corrected Chiroptera" gives the Viterbi P-value after FDR correction along the Chiroptera ancestral branch. The Family ID is the PANTHER family ID. Where multiple PANTHER families were collapsed, IDs were concatenated. If no human protein was present in a family, no PANTHER ID was not assigned, and an internal identifier used. Expansion/Contraction was determined by comparing the Chiroptera ancestor to the inferred scrotiferan ancestor.
(See separate file)

Supplementary Table 13: The number of 286 conserved miRNA gene copies across 48 mammalian taxa based on the *de novo* genomic prediction using the Infernal pipeline.
(See separate file)

Supplementary Table 14: Oligonucleotide sequences used for cloning. Restriction site tags and spacers between individual miRNA binding sites are highlighted in bold, mature miRNAs are italic, and miRNA seed sequences are underlined.

Insert for cloning	SENSE OLIGO (5’→3’)	ANTISENSE OLIGO (5’→3’)
Bat-miR-19125	**CTAGA**TCCCTTGGAAAGAGCCTTGTTTTGGAAGGGAAGGGGGAAGAGGCTCTGCCCTTGACC*TCTACCCTTTGTTTCCTTCCAGC*CTTTGTCCAGGAGTTGAGGAAGAGG**G**	**TCGAC**CCTCTTCCTCAACTCCTGGACAAAGGCTGGAAGGAAACAAAGGGTAGAGGTCAAGGGCAGAGCCTCTTCCCCCTTCCCTTCCAAAACAAGGCTCTTTCCAAGGGA**T**
Bat-miR-4665	**CTAGA**CCCCTACTTGCAGTTGGTCCGACGG*TTGTGGGTTATTGTTAAG*CTGATTAACATTGTCTCCCTCCACACAACCACATTTGACTGACTTTGTATTTTGCCCTAGTC**G**	**TCGAC**GACTAGGGCAAAATACAAAGTCAGTCAAATGTGGTTGTGTGGAGGGAGACAATGTTAATCAGCTTAACAATAACCCACAACCGTCGGACCAACTGCAAGTAGGGG**T**
Bat-miR-6665	**CTAGA**ACAAAGTAGGTTAGATCTTGCCAGA*TTAGGTGGAGATTCTCGCAGGG*GGAGTTCAACTTCATATACCCTTGCAAGATACTCCTCTGTCTGGAAAGGTCTTCCTCT**G**	**TCGAC**AGAGGAAGACCTTTCCAGACAGAGGAGTATCTTGCAAGGGTATATGAAGTTGAACTCCCCCTGCGAGAATCTCCACCTAATCTGGCAAGATCTAACCTACTTTGT**T**
Bat-miR-19125_sensor	**TCGAG**GCTGGAAGGAAACAAAGGGTAGAGAATATGCTGGAAGGAAACAAAGGGTAGA**T**	**CTAGA**TCTACCCTTTGTTTCCTTCCAGCATATTCTCTACCCTTTGTTTCCTTCCAGC**C**
Bat-miR-4665_sensor	**TCGAG**CTTAACAATAACCCACAAGAATATCTTAACAATAACCCACAA**T**	**CTAGA**TTGTGGGTTATTGTTAAGATATTCTTGTGGGTTATTGTTAAG**C**
Bat-miR-6665_sensor	**TCGAG**CCCTGCGAGAATCTCCACCTAAGAATATCCCTGCGAGAATCTCCACCTAA**T**	**CTAGA**TTAGGTGGAGATTCTCGCAGGGATATTCTTAGGTGGAGATTCTCGCAGGG**C**
Bat-miR-337	**CTAGA**ACAGTCAGTAAGTGGGGGGTGAGAACGGCTTCATCCAGGAGTTGATGCCCAGTTATCCAGC*GCCTAGA*TGATGCCTTTCTTCATCCCCTTCAA**G**	**TCGAC**TTGAAGGGGATGAAGAAAGGCATCATCTAGGCGCTGGATAACTGGGCATCAACTCCTGGATGAAGCCGTTCTCACCCCCCACTTACTGACTGT**T**
hsa-miR-337	**CTAGA**GTAGTCAGTAGTTGGGGGGTGGGAACGGCTTCATACAGGAGTTGATGCACAGTTATCCAGC*TCCTATA*TGATGCCTTTCTTCATCCCCTTCAA**G**	**TCGAC**TTGAAGGGGATGAAGAAAGGCATCATATAGGAGCTGGATAACTGTGCATCAACTCCTGTATGAAGCCGTTCCCACCCCCCAACTACTGACTAC**T**
Bat-miR-337_sensor	**TCGAG**GAAGAAAGGCATCATCTAGGCGGAATATGAAGAAAGGCATCATCTAGGCG**T**	**CTAGA**CGCCTAGATGATGCCTTTCTTCATATTCCGCCTAGATGATGCCTTTCTTC**C**
hsa-miR-337_sensor	**TCGAG**GAAGAAAGGCATCATATAGGAGGAATATGAAGAAAGGCATCATATAGGAG**T**	**CTAGA**CTCCTATATGATGCCTTTCTTCATATTCCTCCTATATGATGCCTTTCTTC**C**

Supplementary Table 15: The statistics of 3'UTR analysis for 6 bat genomes.

	Total 3UTR	Different genes with overlapped 3UTR loci	After merging overlapped coordinates	Pseudo 3UTR
M. molossus	13,671	290	11,912	8,613
M. myotis	13,263	406	11,024	8,811
P. kuhlii	6,891	182	6,372	5,612
P. discolor	15,122	476	12,196	9,030
R. ferrumequinum	7,913	226	7,194	6,346
R. aegyptiacus	16,115	327	13,394	9,519

Supplementary Table 16: The gene targets of human and bat miR-337 predicted by RNAhybrid and miranda. The gene targets, which were specific to bat and human and were shared between bat and human, are listed respectively.

(See separate file)

Supplementary Table 17: Summary of miRNA sequencing and analysis in 6 bat genomes. Note that *P. discolor* brain includes samples of cortex, cerebellum and striatum.

Species	Tissue	Accession Number	Raw reads	Mapping rate	Known miRNA	Novel miRNA
Myotis myotis	Brain	SRR10153055	44,256,216	90.3%	329	242
	Kidney	SRR10153054	41,823,612	82.7%		
	Liver	SRR10153043	42,013,686	81.8%		
Pipistrellus kuhlii	Brain	SRR10153039	46,857,126	92.6%	258	122
	Kidney	SRR10153038	35,303,058	73.2%		
	Liver	SRR10153037	39,045,247	75.0%		
Molossus molossus	Brain	SRR10153042	35,168,361	93.8%	286	229
	Kidney	SRR10153041	44,074,092	91.6%		
	Liver	SRR10153040	36,055,759	86.5%		
Phyllostomus discolor	Brain	SRR10153046<br>SRR10153047<br>SRR10153048	107,798,649	94.7%	284	133
	Kidney	SRR10153045	39,805,199	91.7%		
	Liver	SRR10153044	27,597,872	89.9%		
Rhinolophus ferrumequinum	Brain	SRR10153036	52,426,146	91.8%	332	261
	Kidney	SRR10153053	50,908,913	91.1%		
	Liver	SRR10153052	48,450,654	86.2%		
Rousettus aegyptiacus	Brain	SRR10153051	25,458,780	91.6%	325	217
	Kidney	SRR10153050	45,673,617	91.6%		
	Liver	SRR10153049	33,831,764	91.0%		

Supplementary Table 18: The summary of 12 novel miRNA at the ancestral bat lineage. These newly-evolved miRNAs were not found in any other species.
(See separate file)

Supplementary Table 19: Overview of species and tissues used for high molecular weight genomic DNA (HMW gDNA) extraction.

Species	Sex	Provided by	Year of collection	Location
Molossus molossus	male	Dina Dechmann	2018	Gamboa, Panama (9.1165° N, 79.6965° W)
Myotis myotis	female	Emma Teeling & Sébastien Puechmaille	2015	Limerzel, France (47.6333° N, 2.3500° W)
Pipistrellus kuhlii	male	Emma Teeling & Andrea Locatelli	2017	Bergamo, Italy (45.7430° N, 9.5831° E)
Phyllostomus discolor	male	Sonja Vernes	2016	Munich, Germany (Captive colony)
Rhinolophus ferrumequinum	female	Gareth Jones	2016	United Kingdom (51.7108° N, 2.2776° W)
Rousettus aegyptiacus	male	Sonja Vernes	2017	Berkeley, USA (Captive colony)

Supplementary Table 20: The information of gDNA extraction for 6 bat species. The table includes gDNA extraction protocol, size range of extracted gDNA determined by PFGE, and applied technologies (CLR: continuous long reads, PCE: phenol-chloroform-extraction). MA= MagAttract used for Pacbio CLR and 10x (40-60kb); Plug= 50-500kb only used for Bionano.

Species	Tissue	Extraction	Size range (kb)	PacBio CRL	Bionano	10x linked reac
Molossus molossus	muscle	PCE	50 - 150	X		
	liver	plug	50 - > 500		X	X
Myotis myotis	muscle	PCE	50 - 300	X		X
	muscle	Plug	50 - > 500		X	
Pipistrellus kuhlii	muscle	PCE	50 - 250	X		X
	heart	Plug	50 - 400		X	
Phyllostomus discolor	muscle	Plug<br>MA	40-60		X	
Rhinolophus ferrumequinum	lung	PCE	50 - 250	X		X
	lung	Plug	VGL		X	
Rousettus aegyptiacus	muscle	PCE	50 - 250	X		
	liver	plug	50 - > 500		X	X

Supplementary Table 21: The information of PacBio CLR library preparation and sequencing.

Species	Shearing size (kb)	Size selection (kb)	PacBio polymerase	No. of SMRT cells	Average yield per SMRT cell (Gb)	Average insert N50 per SMRT cell (kb)
Molossus molossus	75	25	2.1	26	4.8	23.7
Myotis myotis	35	12 - 15	2.0	39	3.31	14.3
Pipistrellus kuhlii	35 - 40	15 - 20	2.0	48	2.55	13.76
Phyllostomus discolor	35 - 40	12 - 15	2.0	43	4.10	15.05
Rhinolophus ferrumequinum	40	18	2.0	25	5.09	18.25
Rousettus aegyptiacus	60	18	2.0	35	3.92	18.08

Supplementary Table 22: Sequencing depth, effective genome coverage and mean molecular length calculated by the 10x Supernova tool.

Species	Long gDNA	megasize gDNA	Sequenced fragments (Mi reads)	Genome coverage (linked reads)	Mean molecular length (kb)
Molossus molossus	-	X	354	49x	131.9
Myotis myotis	X	-	319	44x	29.4
Pipistrellus kuhlii	X	-	327	46x	19.7
Phyllostomus discolor	X	-	789	109x	16.0
Rhinolophus ferrumequinum	X	-	345	48x	28.8
Rousettus aegyptiacus	-	X	365	51	97.9

Supplementary Table 23: Overview of samples used for Iso-seq.

Species	Year of collection	Location	Tissue	Storage	Extraction	RIN
Molossus molossus	2017	Panama	brain	snap frozen	RNAeasy	9.1
			testes	snap frozen	RNAeasy	9.0
Myotis myotis	2015	Limerzel, France	brain	snap frozen	RNAeasy	8.4
			liver	snap frozen	RNAeasy	8.9
			kidney	snap frozen	RNAeasy	9.1
Pipistrellus kuhlii	2015	Italy	brain	snap frozen	Chloroform-Isopropanol	8.1
Phyllostomus discolor	2016	Munich, Germany, (LMU captive colony)	brain	snap frozen	Relia prep	7.4
			testes	snap frozen	RNAeasy	9.7
Rhinolophus ferrumequinum	2018	United Kingdom	brain	snap frozen	RNAeasy	9.1
Rousettus aegyptiacus	2018	Berkeley, USA, (captive colony)	brain	Qiazol frozen	RNAeasy	8.6
			testes	Qiazol frozen	RNAeasy	8.7

Supplementary Table 24: Statistics of PacBio dataset. The Raw data set contains all PacBio subreads longer then 500 b. The Filtered_1 data set contains only statistics for the longest read of each Zero-mode waveguide (ZMW). The Filtered_2 data set, that was used for the assembly, contains only the longest subread per ZMW with a minimum length of 4 kb.

		Number of SMRT Cells	Number of Reads (M)	Total Base Pairs (Gbp)	Estimated Coverage	Average Read Length (Kbp)	Longest Read (Kbp)	Finish Date
M. myotis	*Raw*	48	21.4	182.1	90.9	8.5	150.6	May 2017
	Filtered_1		16.4	150.4	75.1	9.2		
	Filtered_2		11.4	140.2	70.0	13.8		
P. kuhlii	*Raw*	48	16.0	143.6	80.8	9.0	150.4	Jun 2017
	Filtered_1		12.8	122.4	68.9	9.6		
	Filtered_2		9.2	115.0	64.7	12.6		
R. ferrumequinum	*Raw*	25	14.5	152.0	73.3	10.5	161.2	Aug 2017
	Filtered_1		11.3	127.1	61.2	11.2		
	Filtered_2		8.2	121.0	58.3	14.8		
P. discolor	*Raw*	43	18.2	163.5	78.0	9.0	148.0	Mar 2018
	Filtered_1		15.6	148.7	71.0	9.5		
	Filtered_2		10.8	138.9	66.3	12.8		
R. aegyptiacus	*Raw*	33	12.0	122.3	64.6	10.2	174.1	Feb 2018
	Filtered_1		11.1	117.5	62.0	10.6		
	Filtered_2		7.7	110.5	58.3	14.4		
M. molossus	*Raw*	26	11.1	135.1	58.3	12.1	101.0	Sep 2018
	Filtered_1		9.0	124.9	53.8	13.8		
	Filtered_2		6.7	120.2	51.8	18.0		

Supplementary Table 25: Statistics of 10x datasets and yields over 100 kb. Raw: raw Illumina sequencing statistics; barcode removed: Illumina read pair statistics after trimming off the 16 bp 10x and 7 bp Illumina barcodes from the R1 reads; Molecule length >100K: Statistics for Molecule lengths that were calculated from the final assemblies and the tool bxcheck (https://github.com/pd3/bxcheck).

		Number of Lanes	Number of Reads (M)	Total Base Pairs (Gbp)	Estimated Coverage	Read Cloud N50 (Kbp)	Finish Date
M. myotis	*Raw*	4	319.4	95.8	47.8	23	Aug 2017
	barcode removed		319.4	88.5	44.2		
	Molecule length >=100K		3.8	1.1	0.5		
P. kuhlii	*Raw*	4	327.4	98.9	55.7	19	Nov 2017
	barcode removed		327.4	91.3	51.4		
	Molecule length >=100K		4.6	1.4	0.8		
R. ferrumequinum	*Raw*	4	345.0	104.2	50.2	30	Nov 2017
	barcode removed		345.0	96.2	46.4		
	Molecule length >=100K		5.4	1.6	0.8		
P. discolor	*Raw*	8	788.9	236.7	113.0	13	Mar 2018
	barcode removed		788.9	218.5	104.3		
	Molecule length >=100K		18.5	5.6	2.7		
R. aegyptiacus	*Raw*	8	365.1	110.2	58.2	94	Oct 2018
	barcode removed		365.1	101.8	53.8		
	Molecule length >=100K		167.9	50.7	26.8		
M. molossus	*Raw*	8	354.8	107.2	46.2	128	Oct 2018
	barcode removed		354.8	99.0	42.7		
	Molecule length >=100K		226.4	68.4	29.5		

Supplementary Table 26: Statistics of Bionano dataset. The filtered data set were used for the *de novo* Bionano assembly and consists of molecules with a minimum length of 150 kb and a minimum of 9 label sites.

		Technology	Number of Molecules (M)	Total Length (Gbp)	Estimated Coverage	Average Molecule Length (Kbp)	Finish Date Where
M. myotis	*Raw*	*DLE1*	27.7	1960	979	71	Nov 2018
	Filtered		2.0	396	198	195	Ploen
P. kuhlii	*Raw*	*DLE1*	17.9	1419	799	79	Nov 2018
	Filtered		1.9	460	259	242	Ploen
R. ferrumequinum	*Raw*	*BSPQI*	6.4	547	263	85	Dec 2017
	Filtered		1.0	313	151	308	Rockefeller
	Raw	*BSSSI*	12.0	1046	504	89	Dec 2017
	Filtered		2.1	597	288	286	Rockefeller
P. discolor	*Raw*	*BSPQI*	6.7	764	365	114	Jun 2017
	Filtered		1.8	480	229	265	Rockefeller
	Raw	*BSSSI*	2.7	315	150	118	Jun 2017
	Filtered		0.7	186	89	151	Rockefeller
R. aegyptiacus	*Raw*	*DLE1*	2.4	410	216	169	Feb 2019
	Filtered		1.0	320	169	309	Dresden
M. molossus	*Raw*	*DLE1*	9.2	948	409	102	Oct 2017
	Filtered		2.0	462	199	234	Ploen

Supplementary Table 27: Statistics of Bionano restriction map assemblies.

	Technology	Number of Maps	Total Length (Gbp)	Average Map Length (Mbp)	N50 Map Length (Mbp)
M. myotis	*DLE1*	342	2.22	6.5	44.8
P. kuhlii	*DLE1*	474	1.78	3.7	13.4
R. ferrumequinum	*BSPQI*	1390	2.12	1.5	2.3
	BSSSI	812	2.36	2.9	6.3
P. discolor	*BSPQI*	1193	2.42	2.0	3.1
	BSSSI	1345	2.07	1.5	2.4
R. aegyptiacus	*DLE1*	58	1.96	33.8	88.5
M. molossus	*DLE1*	222	2.56	11.6	80.4

Supplementary Table 28: Statistics of Hi-C dataset. The estimated coverage was computed by using the final assembly sizes (including gap size).

	Number of Cycles	Number of Reads (M)	Total Base Pairs (Gbp)	Estimated Coverage	Finish Date
M. myotis	80 PE	376.4	30.1	15.0	Jun 2017
P. kuhlii	80 PE	389.8	31.2	17.6	May 2017
R. ferrumequinum	150 PE	306.5	46.3	22.3	Sep 2017
P. discolor	150 PE	1331.4	199.7	95.3	Feb 2018
R. aegyptiacus	150 PE	924.0	139.5	73.7	Dec 2018
M. molossus	150 PE	975.1	147.2	63.5	Feb 2019

Supplementary Table 29: The genomes after read assembly. (all lengths in Mb, NG50 correspond to *post hoc* genome size of the final assemblies.)

Species	Primary Contigs			Alternate Contigs			Discarded Contigs		
	Number	Total Length	NG50	Number	Total Length	N50	Number	Total Length	N50
M. myotis	598	1,976	11.80	671	55	0.10	406	22	0.06
P. kuhlii	527	1,765	10.24	624	53	0.10	270	13	0.05
R. ferrumequinum	324	2,056	21.74	67	58	0.10	120	7	0.07
P. discolor	421	2,056	16.15	234	20	0.10	245	14	0.06
R. aegyptiacus	260	1,866	21.74	108	10	0.11	141	9	0.07
M. molossus	396	2,261	21.58	565	89	0.18	205	15	0.08

Supplementary Table 30: The genomes after Bionano scaffolding. (all lengths in Mb; NG50 correspond to *post hoc* genome size of the final assemblies. Δ Number refers to the number of contig breaks. Δ NG50 refers to difference between the NG50 of Bionano contigs and NG50 of locally-phased contigs.)

Species	Scaffolds				Δ Primary Contigs	
	Number	Total length	Maximum length	NG50 length	Δ Number	Δ NG50
M. myotis	119	2,003	113	62.10	+9	+0.00
P. kuhlii	223	1,776	92	48.91	+8	+0.00
R. ferrumequinum	49	2,076	201	96.92	+16	+0.00
P. discolor	99	2,095	121	47.75	+27	-1.00
R. aegyptiacus	49	1,951	178	93.67	+10	+0.00
M. molossus	76	2,319	132	84.84	+11	+0.00

Supplementary Table 31: Statistics of 6 bat genomes after Hi-C scaffolding. (all lengths in Mb, NG50 correspond to *post hoc* genome size of the final assemblies. Δ Number refers to the number of contig breaks. Δ NG50 refers to difference between the NG50 of Hi-C contigs and NG50 of Bionano contigs.)

Species	Scaffolds				Δ Primary Contigs	
	Number	Total Length	Maximum Length	NG50	Δ Number	Δ NG50
M. myotis	100	2,003	218	89.76	+22	+0.00
P. kuhlii	202	1,776	197	80.24	+62	+0.00
R. ferrumequinum	48	2,076	128	90.45	+7	+0.00
P. discolor	64	2,095	215	104.13	+3	+0.00
R. aegyptiacus	40	1,951	186	121.83	+2	+0.00
M. molossus	67	2,319	230	100.25	+5	+0.00

Supplementary Table 32: Number of gene evidence separated by type of evidence that were used to annotate coding genes in the genomes of the six bats. * the reference species in this projection was *Myotis lucifugus* (Ensembl gene annotation), while for all other projections, we used our *Myotis myotis* gene annotation.

Evidence	Molossus	Myotis	Phyllostomus	Pipistrellus	Rhinolophus	Rousettus
Human Projections	76,605	76,781	76,363	74,627	77,238	76,670
Mouse Projections	49,206	50,555	49,750	48,964	49,916	49,319
Bat Projections	51,471	20241 *	50,790	53,358	49,862	49,222
TAMA filtered transcripts	25,046	29,148	16,326	19,236	25,099	43,004
FLNC, ANGEL Positive transcript	62,303	61,638	107,272	28,398	25,866	87,623
GenomeThreader Alignments	8,449 (stringent parameters), 18,556 (lax parameters)	23,787	14,372	14,132	25,795	13,192
Augustsus single genome mode	64,664	57,976	51,137	39,673	42,882	44,162
Augustus CGP	24,729	23,800	24,067	21,100	22,001	23,373

Supplementary Table 33: Related species used for Genome Threader alignments. Details of publicly available cDNA and protein data aligned to 6 genome assemblies for annotation from a related species.

Reference species	Query species	Query source	No. of query cDNA	No. of query peptides	Related at taxonomic rank
Molossus molossus	*Miniopterus natalensis*	Refseq	25,266	25,266	Superfamily: Vespertilionoidea
Myotis myotis	*Myotis lucifugus*	Ensembl	22,432	20,719	Genus: Myotis
Phyllostomus discolor	*Desmodus rotundus*	Refseq	28,829	28,829	Family: Phyllostomidae
Pipistrellus kuhlli	*Eptesicus fuscus*	Refseq	18,724	18,263	Subfamily: Vespertilioninae
Rhinolophus ferrumequinum	*Rhinolophus sinicus*	Refseq	29,785	29,785	Genus: Rhinolophus
Rousettus aegyptiacus	*Pteropus vampyrus*	Ensembl	18,086	17,053	Subamily: Pteropodinae

Supplementary Table 34: Sources of RNA-Seq and Iso-seq transcriptomic data that we used for annotating genes.

Species	Tissue	Accession Number/ Bioproject
Myotis myotis	Brain	SRR11528221
	Heart	SRR11528219; SRR11528220
	Liver	SRR11528217; SRR11528218
	Kidney	SRR11528215; SRR11528216
Molossus molossus	Blood	SRR11526509 - SRR11526516
Phyllostomus discolor	Brain	PRJNA291690
Pipistrellus kuhlii	Fibroblast	PRJNA565655
Rhinolophus ferrumequinum	Brain	SRR1048140
	Brain	SRR1048142
	Liver	SRR2754983
	Liver	SRR2757329
	Intestine	SRR6749599
	Intestine	SRR6749600
	Intestine	SRR6749601
	Intestine	SRR6749602
Rousettus aegyptiacus	Testes	SRR2914372
	Liver	SRR2914369
	Kidney	SRR2914360
	Heart	SRR2914359
	Brain	SRR2914295
	Liver	SRR2914059
	Kidney	SRR2913355
	Heart	SRR2913354
	Brain	SRR2913353

Supplementary Table 35: Base pair counts and genome proportion estimates of transposable element classes in each examined taxon.
(See separate file)

Supplementary Table 36: The library of viral protein sequences used for endogenous viral elements (EVE) analysis.
(See separate file)

Supplementary Table 37: The probes of viral proteins gag, pol and env that were used to identify the endogenous retrovirus sequences in 6 bat genomes.

Family	Abbreviation	Name	POL	ENV	GAG
Alpharetroviruses	ALV	Avian Leukosis Virus	AJG42161.1	AJG42162.1	AJG42160.1
	RSV	Rous Sarcoma Virus	CAA48535.1	CAA48536.1	CAA48534.1
Betaretroviruses	SRV	Simian retrovirus 2	ATN28189.1	ATN28190.1	ATN28187.1
	JSRV	Jaagsiekte sheep retrovirus	AAD45226.1	NP_041188.1	AAA89180.1
Gammaretroviruses	DrERV	Desmodus rotundus endogenous retrovirus	AJR27940.1	AJR27937.1	AJR27933.1
	PoERV	Porcine endogenous retrovirus	AAL38193.1	CAA76583.1	ADG27335.1
	KoRV	Koala Retrovirus	YP_009513211.1	YP_009513212.1	AAF15097.1
Deltaretrovirus	BLV	Bovine leukemia virus	BAA00544.1	ALB75304.1	AAC82585.1
	HTLV	Human T-lymphotropic virus 2	AAD34842.1	AAD34843.1	AAB59884.1
Epsilonretroviruses	WEHV	Walleye epidermal hyperplasia virus 1	AAD30048.1	AAD30049.1	AAD30047.1
Lentiviruses	HIV-1	Human immunodeficiency virus 1	NP_789740.1	AAC82596.1	AAC82593.1
	FIV	Feline immunodeficiency virus	CAA40318.1	AAB59940.1	AAB59936.1
Spumaretroviruses	BFV	Bovine foamy virus	AFR79239.1	AAB68771.1	AAB68769.1
	FFV	Feline foamy virus	CAA11581.1	CAA70076.1	CAA70074.1

Supplementary Table 38: The Number of viral integrations in analysed genomes. The number of all retroviral integrations found in six bat genomes for each viral class and protein (Pol, Gag, Env). Pol sequences were additionally searched in seven reference genomes and their numbers were compared.

(See separate file)

Supplementary Table 39: Divergence time estimates (in millions of years) using r8s. Their respective nodes are relative to tree topology 1.

Node	Divergence Time (Mya)
Eutherian Root	99.37
Atlantogenata	93.26
Afrotheria	71.7
Paenungulata	59.2
Tubulidentata+Afrosoricida	68.91
Boreoeutheria	94.67
Euarchontoglires	82.85
Primates	66
Haplorhini	61.26
Simiiformes	29.21
Catarrhini	20.55
Hominidae	10
Homininae	5.73
Pan+Homo	5.11
Saimiri+Callithrix	12.93
Strepsirrhini	48.84
Glires	81.85
Rodentia+Lagomorpha	78.81
Lagomorpha	50.76
Rodentia	66
Hystricomorpha	64.86
Muroidea	34.86
Murinae	21.61
Microtus+Cricetulus	28.24
Heterocephalidae+Caviidae	37.41
Laurasiatheria	88.14
Eulipotyphla	82.45
Erinaceidae+Soricidae	76.85
Scrotifera	77.26
Carnivora+Pholidota+Cetartiodactyla+Perissodactyla	74.67
Cetartiodactyla+Perissodactyla	73.13
Cetartiodactyla	55.86
Sus+Bos+Cetacea	52.74
Bos+Cetacea	47.51
Cetacea	34
Perissodactyla	55.5
Carnivora+Pholidota	69.9
Pholidota	8.03
Carnivora	43
Caniformia	34.64
Arctoidea	27.12
Leptonychotes+Mustela	25.53
Chrioptera	63.39
Yangochiroptera	52.79
Molossus+Vespertilionidae	47.39
Vespertilionidae	24.7
Yinpterochiroptera	56.64

Supplementary Table 40: The 15 topologies showing different arrangements of Laurasiatheria and the number of gene trees supporting them with the highest likelihood. Positions of other mammals are fixed relative to the coding-gene supermatrix topology (topology 1).
(See separate file)

Supplementary Table 41: The name, alignment length and number of taxa present for 10,857 conserved non-coding elements (CNEs).
(See separate file)

Supplementary Table 42: RF distances between all gene trees.
(See separate file)

Supplementary Table 43: All 2,453 genes relating to ageing/immunity/metabolism used in selection analysis with PAML and their taxonomic representation.
(See separate file)

Supplementary Table 44: Protein structure prediction of the genes under positive selection in bats.
(See separate file)

Supplementary Table 45: The miRNA families that were gained and lost in the bat lineages.
(See separate file)

Supplementary Table 46: Investigation of conserved one-to-one single-copy miRNA genes in 6 bat species compared to other 42 mammalian taxa. The conservation of mature miRNA and their seed regions was manually curated. 5p and 3p indicate the coordinates of 5p & 3p mature miRNA in multiple alignments. The empirical expression data were based on miRBase (release 22). The missing values indicate that the miRNA does not have either 5p or 3p mature sequences.
(See separate file)

Supplementary Data File 1: *De novo* curated transposable elements combined with a vertebrate library of known TEs in RepBase.
(See separate file)

Supplementary Data File 2: Alignments of 12,931 protein-coding genes across 48 mammals.
(See separate file)

Supplementary Data File 3: Predicted 3D protein structures of candidate genes.
(See separate file)

References

1 Teeling, E. C. *et al.* A molecular phylogeny for bats illuminates biogeography and the fossil record. *Science* **307**, 580-584 (2005).

2 Teeling, E. C. *et al.* Bat Biology, Genomes, and the Bat1K Project: To Generate Chromosome-Level Genomes for All Living Bat Species. *Annu Rev Anim Biosci* **6**, 23-46 (2018).

3 A reference standard for genome biology. *Nat Biotechnol* **36**, 1121 (2018).

4 Kitzman, J. O. Haplotypes drop by drop. *Nat Biotechnol* **34**, 296-298 (2016).

5 Mostovoy, Y. *et al.* A hybrid approach for *de novo* human genome sequence assembly and phasing. *Nat Methods* **13**, 587-590 (2016).

6 Nowoshilow, S. *et al.* The axolotl genome and the evolution of key tissue formation regulators. *Nature* **554**, 50-55 (2018).

7 Grohme, M. A. *et al.* The genome of Schmidtea mediterranea and the evolution of core cellular mechanisms. *Nature* **554**, 56-61 (2018).

8 Myers, E. W. The Dresden AZZembLER for long read projects. Available at:https://dazzlerblog.wordpress.com/.

9 Tischler, G. & Myers, E. W. Non hybrid long read consensus using local *de Bruijn* graph assembly. *bioRxiv*, 106252 (2017).

10 Tischler, G. Haplotype and repeat separation in long reads. *bioRxiv*, 145474 (2017).

11 Myers, E. W. Detecting and Masking Repeats, Available at: https://dazzlerblog.wordpress.com/2016/04/01/detecting-and-soft-masking-repeats/.

12 Garrison, E. M., G. . Haplotype-based variant detection from short-read sequencing. *arXiv*, arXiv:1207.3907 (2012).

13 Kerpedjiev, P. *et al.* HiGlass: web-based visual exploration and analysis of genome interaction maps. *Genome Biol* **19**, 125 (2018).

14 Li, H. & Durbin, R. Fast and accurate short read alignment with Burrows-Wheeler transform. *Bioinformatics* **25**, 1754-1760 (2009).

15 Ghurye, J., Pop, M., Koren, S., Bickhart, D. & Chin, C. S. Scaffolding of long read assemblies using long range contact information. *BMC Genomics* **18**, 527 (2017).

16 Bradnam, K. R. *et al.* Assemblathon 2: evaluating *de novo* methods of genome assembly in three vertebrate species. *GigaScience* **2**, 10 (2013).

17 Koren, S. *et al.* *De novo* assembly of haplotype-resolved genomes with trio binning. *Nat Biotechnol* **36**, 1174-1182 (2018).

18 Rhie, A. *et al.* Towards complete and error-free genome assemblies of all vertebrate species. *bioRxiv*. doi: https://doi.org/10.1101/2020.05.22.110833 (2020).

19 Kent, W. J., Baertsch, R., Hinrichs, A., Miller, W. & Haussler, D. Evolution's cauldron: duplication, deletion, and rearrangement in the mouse and human genomes. *Proc Natl Acad Sci U S A* **100**, 11484-11489 (2003).

20 Sharma, V., Schwede, P. & Hiller, M. CESAR 2.0 substantially improves speed and accuracy of comparative gene annotation. *Bioinformatics* **33**, 3985-3987 (2017).

21 Gremme, G., Brendel, V., Sparks, M. E. & Kurtz, S. Engineering a software tool for gene structure prediction in higher organisms. *Information and Software Technology* **47**, 965-978 (2005).

22 Kim, D., Langmead, B. & Salzberg, S. L. HISAT: a fast spliced aligner with low memory requirements. *Nat Methods* **12**, 357-360 (2015).

23 Li, H. *et al.* The Sequence Alignment/Map format and SAMtools. *Bioinformatics* **25**, 2078-2079 (2009).

24 Pertea, M. *et al.* StringTie enables improved reconstruction of a transcriptome from RNA-seq reads. *Nat Biotechnol* **33**, 290-295 (2015).

25 Camacho, C. *et al.* BLAST+: architecture and applications. *BMC Bioinformatics* **10**, 421 (2009).

26 Colombo, M., Karousis, E. D., Bourquin, J., Bruggmann, R. & Muhlemann, O. Transcriptome-wide identification of NMD-targeted human mRNAs reveals extensive redundancy between SMG6- and SMG7-mediated degradation pathways. *RNA* **23**, 189-201 (2017).
27 Stanke, M., Schoffmann, O., Morgenstern, B. & Waack, S. Gene prediction in eukaryotes with a generalized hidden Markov model that uses hints from external sources. *BMC Bioinformatics* **7**, 62 (2006).
28 Hoff, K. J., Lomsadze, A., Borodovsky, M. & Stanke, M. Whole-Genome Annotation with BRAKER. *Methods Mol Biol* **1962**, 65-95 (2019).
29 Haas, B. J. *et al.* Automated eukaryotic gene structure annotation using EVidenceModeler and the Program to Assemble Spliced Alignments. *Genome Biol* **9**, R7 (2008).
30 Rodriguez, J. M. *et al.* APPRIS 2017: principal isoforms for multiple gene sets. *Nucleic acids Res* **46**, D213-D217 (2018).
31 Waterhouse, R. M. *et al.* BUSCO applications from quality assessments to gene prediction and phylogenomics. *Mol Biol Evol* **35**, 543-548 (2017).
32 Bejerano, G. *et al.* Ultraconserved elements in the human genome. *Science* **304**, 1321-1325 (2004).
33 Kent, W. J. BLAT--the BLAST-like alignment tool. *Genome Res* **12**, 656-664 (2002).
34 Hecker, N. & Hiller, M. A genome alignment of 120 mammals highlights ultraconserved element variability and placenta-associated enhancers. *GigaScience* **9**, giz159 (2020).
35 Platt, R. N., 2nd, Blanco-Berdugo, L. & Ray, D. A. Accurate Transposable Element Annotation Is Vital When Analyzing New Genome Assemblies. *Genome Biol Evol* **8**, 403-410 (2016).
36 Smit, A., Hubley, R. & Green, P. *RepeatMasker Open-4.0. 2013-2015. Available at:* http://www.repeatmasker.org.
37 Kumar, S. & Subramanian, S. Mutation rates in mammalian genomes. *Proc Natl Acad Sci U S A* **99**, 803-808 (2002).
38 Edgar, R. C. MUSCLE: multiple sequence alignment with high accuracy and high throughput. *Nucleic Acid Res* **32**, 1792-1797 (2004).
39 Capella-Gutierrez, S., Silla-Martinez, J. M. & Gabaldon, T. trimAl: a tool for automated alignment trimming in large-scale phylogenetic analyses. *Bioinformatics* **25**, 1972-1973 (2009).
40 Rice, P., Longden, I. & Bleasby, A. EMBOSS: the European Molecular Biology Open Software Suite. *Trends Genet* **16**, 276-277 (2000).
41 Abrusan, G., Grundmann, N., DeMester, L. & Makalowski, W. TEclass--a tool for automated classification of unknown eukaryotic transposable elements. *Bioinformatics* **25**, 1329-1330 (2009).
42 Fu, L., Niu, B., Zhu, Z., Wu, S. & Li, W. CD-HIT: accelerated for clustering the next-generation sequencing data. *Bioinformatics* **28**, 3150-3152 (2012).
43 Li, W. & Godzik, A. Cd-hit: a fast program for clustering and comparing large sets of protein or nucleotide sequences. *Bioinformatics* **22**, 1658-1659 (2006).
44 Wicker, T. *et al.* A unified classification system for eukaryotic transposable elements. *Nat Rev Genet* **8**, 973-982 (2007).
45 Pace, J. K., 2nd & Feschotte, C. The evolutionary history of human DNA transposons: evidence for intense activity in the primate lineage. *Genome Res* **17**, 422-432 (2007).
46 Platt, R. N., 2nd, Mangum, S. F. & Ray, D. A. Pinpointing the vesper bat transposon revolution using the *Miniopterus natalensis* genome. *Mob DNA* **7**, 12 (2016).
47 Altschul, S. F., Gish, W., Miller, W., Myers, E. W. & Lipman, D. J. Basic local alignment search tool. *J Mol Biol* **215**, 403-410 (1990).
48 Katzourakis, A. & Gifford, R. J. Endogenous viral elements in animal genomes. *PLoS Genet* **6**, e1001191 (2010).
49 Larsson, A. AliView: a fast and lightweight alignment viewer and editor for large datasets. *Bioinformatics* **30**, 3276-3278 (2014).
50 Abascal, F., Zardoya, R. & Posada, D. ProtTest: selection of best-fit models of protein evolution. *Bioinformatics* **21**, 2104-2105 (2005).

51 Stamatakis, A. RAxML version 8: a tool for phylogenetic analysis and post-analysis of large phylogenies. *Bioinformatics* **30**, 1312-1313 (2014).
52 Taylor, D. J., Dittmar, K., Ballinger, M. J. & Bruenn, J. A. Evolutionary maintenance of filovirus-like genes in bat genomes. *BMC Evol Biol* **11**, 336 (2011).
53 Hayward, A., Grabherr, M. & Jern, P. Broad-scale phylogenomics provides insights into retrovirus-host evolution. *Proc Natl Acad Sci U S A* **110**, 20146-20151 (2013).
54 Skirmuntt, E. C. & Katzourakis, A. The evolution of endogenous retroviral envelope genes in bats and their potential contribution to host biology. *Virus Res* **270**, 197645 (2019).
55 Xu, X., Zhao, H., Gong, Z. & Han, G. Z. Endogenous retroviruses of non-avian/mammalian vertebrates illuminate diversity and deep history of retroviruses. *PLoS Patholog* **14**, e1007072 (2018).
56 Katzourakis, A., Tristem, M., Pybus, O. G. & Gifford, R. J. Discovery and analysis of the first endogenous lentivirus. *Proc Natl Acad Sci U S A* **104**, 6261-6265 (2007).
57 Farkasova, H. *et al.* Discovery of an endogenous Deltaretrovirus in the genome of long-fingered bats (Chiroptera: Miniopteridae). *Proc Natl Acad Sci U S A* **114**, 3145-3150 (2017).
58 Katzourakis, A., Gifford, R. J., Tristem, M., Gilbert, M. T. & Pybus, O. G. Macroevolution of complex retroviruses. *Science* **325**, 1512 (2009).
59 Wu, Z. *et al.* Virome analysis for identification of novel mammalian viruses in bat species from Chinese provinces. *J Virol* **86**, 10999-11012 (2012).
60 Katzourakis, A. *et al.* Larger mammalian body size leads to lower retroviral activity. *PLoS Pathog* **10**, e1004214 (2014).
61 Hron, T. *et al.* Remnants of an Ancient Deltaretrovirus in the Genomes of Horseshoe Bats (Rhinolophidae). *Viruses* **10**, 185 (2018).
62 Ranwez, V., Douzery, E. J. P., Cambon, C., Chantret, N. & Delsuc, F. MACSE v2: Toolkit for the Alignment of Coding Sequences Accounting for Frameshifts and Stop Codons. *Mol Biol Evol* **35**, 2582-2584 (2018).
63 Kalyaanamoorthy, S., Minh, B. Q., Wong, T. K. F., von Haeseler, A. & Jermiin, L. S. ModelFinder: fast model selection for accurate phylogenetic estimates. *Nat Methods* **14**, 587-589 (2017).
64 Nguyen, L. T., Schmidt, H. A., von Haeseler, A. & Minh, B. Q. IQ-TREE: a fast and effective stochastic algorithm for estimating maximum-likelihood phylogenies. *Mol Biol Evol* **32**, 268-274 (2015).
65 Hoang, D. T., Chernomor, O., von Haeseler, A., Minh, B. Q. & Vinh, L. S. UFBoot2: Improving the Ultrafast Bootstrap Approximation. *Mol Biol Evol* **35**, 518-522 (2018).
66 Sanderson, M. J. r8s: inferring absolute rates of molecular evolution and divergence times in the absence of a molecular clock. *Bioinformatics* **19**, 301-302 (2003).
67 Foley, N. M., Springer, M. S. & Teeling, E. C. Mammal madness: is the mammal tree of life not yet resolved? *Philos Trans R Soc Lond B Bio Sci* **371**, 20150140 (2016).
68 Meredith, R. W. *et al.* Impacts of the Cretaceous Terrestrial Revolution and KPg extinction on mammal diversification. *Science* **334**, 521-524 (2011).
69 Degnan, J. H. & Rosenberg, N. A. Discordance of species trees with their most likely gene trees. *PLoS Genet* **2**, e68 (2006).
70 Shimodaira, H. An approximately unbiased test of phylogenetic tree selection. *Syst Biol* **51**, 492-508 (2002).
71 Springer, M. S. & Gatesy, J. An ABBA-BABA Test for Introgression Using Retroposon Insertion Data. *bioRxiv* (2019).
72 Charlesworth, B. Fundamental concepts in genetics: effective population size and patterns of molecular evolution and variation. *Nat Rev Genet* **10**, 195-205 (2009).
73 Hobolth, A., Andersen, L. N. & Mailund, T. On computing the coalescence time density in an isolation-with-migration model with few samples. *Genetics* **187**, 1241-1243 (2011).
74 Jermiin, L. S., Jayaswal, V., Ababneh, F. M. & Robinson, J. Identifying Optimal Models of Evolution. *Methods Mol Biol* **1525**, 379-420 (2017).
75 Ho, S. Y. & Jermiin, L. Tracing the decay of the historical signal in biological sequence data. *Syst Biol* **53**, 623-637 (2004).

76 Ababneh, F., Jermiin, L. S., Ma, C. & Robinson, J. Matched-pairs tests of homogeneity with applications to homologous nucleotide sequences. *Bioinformatics* **22**, 1225-1231 (2006).
77 Chifman, J. & Kubatko, L. Quartet inference from SNP data under the coalescent model. *Bioinformatics* **30**, 3317-3324 (2014).
78 Vachaspati, P. & Warnow, T. SVDquest: Improving SVDquartets species tree estimation using exact optimization within a constrained search space. *Mol Phylogenet Evol* **124**, 122-136 (2018).
79 Springer, M. S. & Gatesy, J. Delimiting Coalescence Genes (C-Genes) in Phylogenomic Data Sets. *Genes* **9**, 123 (2018).
80 Springer, M. S. & Gatesy, J. The gene tree delusion. *Mol Phylogenet Evol* **94**, 1-33 (2016).
81 Scornavacca, C. & Galtier, N. Incomplete Lineage Sorting in Mammalian Phylogenomics. *Syst Biol* **66**, 112-120 (2017).
82 Bogdanowicz, D. & Giaro, K. Comparing Phylogenetic Trees by Matching Nodes Using the Transfer Distance Between Partitions. *J Comput Biol* **24**, 422-435 (2017).
83 Schliep, K. P. phangorn: phylogenetic analysis in R. *Bioinformatics* **27**, 592-593 (2011).
84 Paradis, E., Claude, J. & Strimmer, K. APE: Analyses of Phylogenetics and Evolution in R language. *Bioinformatics* **20**, 289-290 (2004).
85 Smith, M. D. *et al.* Less is more: an adaptive branch-site random effects model for efficient detection of episodic diversifying selection. *Mol Biol Evol* **32**, 1342-1353 (2015).
86 Team, R. C. R: A Language and Environment for Statistical Computing *R Foundation for Statistical Computing, Vienna, Austria* (2016).
87 Yang, Z. PAML 4: phylogenetic analysis by maximum likelihood. *Mol Biol Evol* **24**, 1586-1591 (2007).
88 Notredame, C., Higgins, D. G. & Heringa, J. T-Coffee: A novel method for fast and accurate multiple sequence alignment. *J Mol Biol* **302**, 205-217 (2000).
89 Mizuta, K. *et al.* Ultrastructural localization of megalin in the rat cochlear duct. *Hear Res* **129**, 83-91 (1999).
90 Kantarci, S. *et al.* Mutations in LRP2, which encodes the multiligand receptor megalin, cause Donnai-Barrow and facio-oculo-acoustico-renal syndromes. *Nat Genet* **39**, 957-959 (2007).
91 Konig, O. *et al.* Estrogen and the inner ear: megalin knockout mice suffer progressive hearing loss. *Faseb J* **22**, 410-417 (2008).
92 Sirmaci, A. *et al.* A truncating mutation in SERPINB6 is associated with autosomal-recessive nonsyndromic sensorineural hearing loss. *Am J Hum Genet* **86**, 797-804 (2010).
93 Tan, J., Prakash, M. D., Kaiserman, D. & Bird, P. I. Absence of SERPINB6A causes sensorineural hearing loss with multiple histopathologies in the mouse inner ear. *Am J Pathol* **183**, 49-59 (2013).
94 de Magalhaes, J. P. & Toussaint, O. Telomeres and telomerase: a modern fountain of youth? *Rejuvenation Res* **7**, 126-133 (2004).
95 Carbon, S. *et al.* AmiGO: online access to ontology and annotation data. *Bioinformatics* **25**, 288-289 (2009).
96 O'Sullivan, T. *et al.* Interleukin-17D mediates tumor rejection through recruitment of natural killer cells. *Cell Rep* **7**, 989-998 (2014).
97 Chen, Q. X. *et al.* Genomic variations within DEFB1 are associated with the susceptibility to and the fatal outcome of severe sepsis in Chinese Han population. *Genes Immun* **8**, 439-443 (2007).
98 Alturaiki, W. *et al.* Expression of the B cell differentiation factor BAFF and chemokine CXCL13 in a murine model of Respiratory Syncytial Virus infection. *Cytokine* **110**, 267-271 (2018).
99 Bao, Y. Q. *et al.* Increased circulating CXCL13 levels in systemic lupus erythematosus and rheumatoid arthritis: a meta-analysis. *Clin Rheumatol* **39**, 193-219 (2019).
100 Maleki, K. T., Cornillet, M. & Bjorkstrom, N. K. Soluble SEMA4D/CD100: A novel immunoregulator in infectious and inflammatory diseases. *Clin Rheumatol* **163**, 52-59 (2016).
101 Liu, Y. J. *et al.* TSLP: an epithelial cell cytokine that regulates T cell differentiation by conditioning dendritic cell maturation. *Annu Rev Immunol* **25**, 193-219 (2007).

102 Hase, K. *et al.* Uptake through glycoprotein 2 of FimH(+) bacteria by M cells initiates mucosal immune response. *Nature* **462**, 226-230 (2009).
103 Killick, K. E. *et al.* Receptor-mediated recognition of mycobacterial pathogens. *Cell Microbiol* **15**, 1484-1495 (2013).
104 Flo, T. H. *et al.* Lipocalin 2 mediates an innate immune response to bacterial infection by sequestrating iron. *Nature* **432**, 917-921 (2004).
105 Xu-Monette, Z. Y. *et al.* Clinical and Biologic Significance of MYC Genetic Mutations in De Novo Diffuse Large B-cell Lymphoma. *Clin Cancer Res* **22**, 3593-3605 (2016).
106 Bergemann, A. D. & Johnson, E. M. The HeLa Pur factor binds single-stranded DNA at a specific element conserved in gene flanking regions and origins of DNA replication. *Mol Cell Biol* **12**, 1257-1265 (1992).
107 Foley, N. M. *et al*. Growing old, yet staying young: The role of telomeres in bats' exceptional longevity. *Sci Adv* **4**, eaao0926 (2018).
108 Huang, Z. *et al.* Longitudinal comparative transcriptomics reveals unique mechanisms underlying extended healthspan in bats. *Nat Ecol Evol* **3**, 1110-1120 (2019).
109 Castrillo, A., Joseph, S. B., Marathe, C., Mangelsdorf, D. J. & Tontonoz, P. Liver X receptor-dependent repression of matrix metalloproteinase-9 expression in macrophages. *J Biol Chem* **278**, 10443-10449 (2003).
110 Laurencikiene, J. & Ryden, M. Liver X receptors and fat cell metabolism. *Int J Obes* **36**, 1494-1502 (2012).
111 Castro, L. F., Lopes-Marques, M., Goncalves, O. & Wilson, J. M. The evolution of pepsinogen C genes in vertebrates: duplication, loss and functional diversification. *PloS One* **7**, e32852 (2012).
112 Linsley, P. S. *et al.* Human B7-1 (CD80) and B7-2 (CD86) bind with similar avidities but distinct kinetics to CD28 and CTLA-4 receptors. *Immunity* **1**, 793-801 (1994).
113 Figenschau, S. L. *et al.* ICAM1 expression is induced by proinflammatory cytokines and associated with TLS formation in aggressive breast cancer subtypes. *Sci Rep* **8**, 11720 (2018).
114 Liu, G. *et al.* Characterization of the peripheral blood transcriptome and adaptive evolution of the MHC I and TLR gene families in the wolf (*Canis lupus*). *BMC Genomics* **18**, 584 (2017).
115 Borrego, F. The CD300 molecules: an emerging family of regulators of the immune system. *Blood* **121**, 1951-1960 (2013).
116 Schotte, R. *et al.* The transcription factor Spi-B is expressed in plasmacytoid DC precursors and inhibits T-, B-, and NK-cell development. *Blood* **101**, 1015-1023 (2003).
117 Condamine, T. *et al.* Tmem176B and Tmem176A are associated with the immature state of dendritic cells. *J Leukoc Biol* **88**, 507-515 (2010).
118 Tsuchida, T. *et al.* The ubiquitin ligase TRIM56 regulates innate immune responses to intracellular double-stranded DNA. *Immunity* **33**, 765-776 (2010).
119 Roy, A., Kucukural, A. & Zhang, Y. I-TASSER: a unified platform for automated protein structure and function prediction. *Nat Protoc* **5**, 725-738 (2010).
120 Yang, J. *et al.* The I-TASSER Suite: protein structure and function prediction. *Nat Methods* **12**, 7-8 (2015).
121 Zheng, W. *et al.* LOMETS2: improved meta-threading server for fold-recognition and structure-based function annotation for distant-homology proteins. *Nucleic Acids Res* **47**, W429-W436 (2019).
122 Pettersen, E. F. *et al.* UCSF Chimera--a visualization system for exploratory research and analysis. *J Comput Chem* **25**, 1605-1612 (2004).
123 Zhang, C., Freddolino, P. L. & Zhang, Y. COFACTOR: improved protein function prediction by combining structure, sequence and protein-protein interaction information. *Nucleic Acids Res* **45**, W291-W299 (2017).
124 Yang, J., Roy, A. & Zhang, Y. Protein-ligand binding site recognition using complementary binding-specific substructure comparison and sequence profile alignment. *Bioinformatics* **29**, 2588-2595 (2013).
125 Yang, J., Roy, A. & Zhang, Y. BioLiP: a semi-manually curated database for biologically relevant ligand-protein interactions. *Nucleic Acids Res* **41**, D1096-D1103 (2013).

126 Rodrigues, C. H., Pires, D. E. & Ascher, D. B. DynaMut: predicting the impact of mutations on protein conformation, flexibility and stability. *Nucleic Acids Res* **46**, W350-W355 (2018).
127 Sharma, V. *et al.* A genomics approach reveals insights into the importance of gene losses for mammalian adaptations. *Nat Commun* **9**, 1215 (2018).
128 Sharma, V. & Hiller, M. Coding Exon-Structure Aware Realigner (CESAR): Utilizing Genome Alignments for Comparative Gene Annotation. *Methods Mol Biol* **1962**, 179-191 (2019).
129 Zerbino, D. R. *et al.* Ensembl 2018. *Nucleic Acids Res* **46**, D754-D761 (2018).
130 Ahn, M., Cui, J., Irving, A. T. & Wang, L. F. Unique Loss of the PYHIN Gene Family in Bats Amongst Mammals: Implications for Inflammasome Sensing. *Sci Rep* **6**, 21722 (2016).
131 Han, M. V., Thomas, G. W., Lugo-Martinez, J. & Hahn, M. W. Estimating gene gain and loss rates in the presence of error in genome assembly and annotation using CAFE 3. *Mol Biol Evol* **30**, 1987-1997 (2013).
132 Katoh, K. & Standley, D. M. MAFFT multiple sequence alignment software version 7: improvements in performance and usability. *Mol Biol Evol* **30**, 772-780 (2013).
133 Guindon, S. & Gascuel, O. A simple, fast, and accurate algorithm to estimate large phylogenies by maximum likelihood. *Syst Biol* **52**, 696-704 (2003).
134 LaRue, R. S. *et al.* Guidelines for naming nonprimate APOBEC3 genes and proteins. *J Virol* **83**, 494-497 (2009).
135 Hayward, J. A. *et al.* Differential Evolution of Antiretroviral Restriction Factors in Pteropid Bats as Revealed by APOBEC3 Gene Complexity. *Mol Biol Evol* **35**, 1626-1637 (2018).
136 Nawrocki, E. P. Annotating functional RNAs in genomes using Infernal. *Methods Mol Biol* **1097**, 163-197 (2014).
137 Kalvari, I. *et al.* Rfam 13.0: shifting to a genome-centric resource for non-coding RNA families. *Nucleic Acids Res* **46**, D335-D342 (2018).
138 Quinlan, A. R. & Hall, I. M. BEDTools: a flexible suite of utilities for comparing genomic features. *Bioinformatics* **26**, 841-842 (2010).
139 Thompson, J. D., Higgins, D. G. & Gibson, T. J. CLUSTAL W: improving the sensitivity of progressive multiple sequence alignment through sequence weighting, position-specific gap penalties and weight matrix choice. *Nucleic Acids Res* **22**, 4673-4680 (1994).
140 Friedlander, M. R., Mackowiak, S. D., Li, N., Chen, W. & Rajewsky, N. miRDeep2 accurately identifies known and hundreds of novel microRNA genes in seven animal clades. *Nucleic Acids Res* **40**, 37-52 (2012).
141 Kozomara, A. & Griffiths-Jones, S. miRBase: annotating high confidence microRNAs using deep sequencing data. *Nucleic Acids Res* **42**, D68-D73 (2014).
142 Berezikov, E. Evolution of microRNA diversity and regulation in animals. *Nat Rev Genet* **12**, 846-860 (2011).
143 Langmead, B. & Salzberg, S. L. Fast gapped-read alignment with Bowtie 2. *Nat Methods* **9**, 357-359 (2012).
144 Enright, A. J. *et al.* MicroRNA targets in *Drosophila*. *Genome Biol* **5**, R1 (2003).
145 Kruger, J. & Rehmsmeier, M. RNAhybrid: microRNA target prediction easy, fast and flexible. *Nucleic Acids Res* **34**, W451-W454 (2006).
146 Huang da, W., Sherman, B. T. & Lempicki, R. A. Systematic and integrative analysis of large gene lists using DAVID bioinformatics resources. *Nat Protoc* **4**, 44-57 (2009).
147 Devanna, P. *et al.* Next-gen sequencing identifies non-coding variation disrupting miRNA-binding sites in neurological disorders. *Hum Genet* **23**, 1375-1384 (2018).
148 Devanna, P., van de Vorst, M., Pfundt, R., Gilissen, C. & Vernes, S. C. Genome-wide investigation of an ID cohort reveals *de novo* 3'UTR variants affecting gene expression. *Hum Genet* **137**, 717-721 (2018).
149 Conway, J. R., Lex, A. & Gehlenborg, N. UpSetR: an R package for the visualization of intersecting sets and their properties. *Bioinformatics* **33**, 2938-2940 (2017).
