# Supplementary material for: Six reference-quality genomes reveal evolution of bat adaptations
Source: Nature. 2020 Jul 22;583(7817):578–84. doi: 10.1038/s41586-020-2486-3 (PMC8075899; doi:10.1038/s41586-020-2486-3)
Supplement: Supplementary file 2 — Reporting Summary [file 41586_2020_2486_MOESM2_ESM.pdf]

nature research

Corresponding author(s): Michael Hiller, Sonja Vernes, Gene Myers, Emma Teeling

Last updated by author(s): Jun 30, 2020

Reporting Summary

Nature Research wishes to improve the reproducibility of the work that we publish. This form provides structure for consistency and transparency in reporting. For further information on Nature Research policies, see our Editorial Policies and the Editorial Policy Checklist.

Statistics

For all statistical analyses, confirm that the following items are present in the figure legend, table legend, main text, or Methods section.

n/a	Confirmed	
☐	☒	The exact sample size (*n*) for each experimental group/condition, given as a discrete number and unit of measurement
☐	☒	A statement on whether measurements were taken from distinct samples or whether the same sample was measured repeatedly
☐	☒	The statistical test(s) used AND whether they are one- or two-sided<br>*Only common tests should be described solely by name; describe more complex techniques in the Methods section.*
☐	☒	A description of all covariates tested
☐	☒	A description of any assumptions or corrections, such as tests of normality and adjustment for multiple comparisons
☐	☒	A full description of the statistical parameters including central tendency (e.g. means) or other basic estimates (e.g. regression coefficient) AND variation (e.g. standard deviation) or associated estimates of uncertainty (e.g. confidence intervals)
☐	☒	For null hypothesis testing, the test statistic (e.g. *F*, *t*, *r*) with confidence intervals, effect sizes, degrees of freedom and *P* value noted<br>*Give P values as exact values whenever suitable.*
☐	☒	For Bayesian analysis, information on the choice of priors and Markov chain Monte Carlo settings
☒	☐	For hierarchical and complex designs, identification of the appropriate level for tests and full reporting of outcomes
☒	☐	Estimates of effect sizes (e.g. Cohen's *d*, Pearson's *r*), indicating how they were calculated

Our web collection on statistics for biologists contains articles on many of the points above.

Software and code

Policy information about availability of computer code

Data collection	Data collection did not involve any software or code.
Data analysis	DAmar (https://github.com/MartinPippel/DAmar); DAZZLER (https://github.com/thegenemyers/); DACCORD (v0.0.14-release-20180525105343); MARVEL (https://github.com/schloi/MARVEL); GenomicsConsensus (https://github.com/PacificBiosciences/GenomicConsensus); Longranger (v2.2.0); FreeBayes (v1.2.0); bcftools (v1.9); Bionano Solve (v3.3); HiGlass (v0.6.3); bwa (v0.7.17-r1194); Arima (https://github.com/ArimaGenomics/mapping_pipeline); Salsa2 (v2.2); Assemblathon 2 (https://github.com/ucdavis-bioinformatics/assemblathon2-analysis); TOGA; CESAR (v2.0); Genome Threader (v1.7.0); HISAT2 (v2.0.0); Samtools (v1.9); StringTie (v1.3.4d); TAMA; ncbi-BLAST+ (v2.6.0); IsoSeq (v3.1.0); Bamtools (v2.4.1); Minimap2 (v2.10-r784-dirty); Bedtools (v2.27.1); Augustus (v3.3.1); BRAKER (v2.1); Multiz (v11.2); EVidenceModeler (v1.1.1); BUSCO (v3); BLAT (v36x2); RepeatMasker (v4.0.9); MUSCLE (v3.8.31); EMBOSS (v1); cd-hit-est (v4.6.6); RM2Bed.py (https://github.com/davidaray/bioinfo_tools); Aliview (v1.25); ERVin (https://github.com/strongles/ervin); Prottest (v3.4.2); RAxML (v8); MACSE (v2.01); IQ-TREE (v1.6.10); UFBoot (v2.0.0); r8s (v1.81); Homo (v2.0); Saturation (v1.0); PAUP* (v4.0b10); SVDquartets; Phangorn R package (v2.5.5); ape R package (v5.3); HyPhy (v2.3.11); R (v3.3.1); PAML (v9.4); T-Coffee; I-TASSER; UCSFChimera (v1.14); DynaMut; CAFE (v4.0); POrthoMCL (https://github.com/etabari/PorthoMCL); MAFFT (v7.310); PhyML (v20120412); Infernal (v1.1.2); CAFE (v4.2.1); Phylip (v3.696); ClustalW (v2.1); Geneious (v7.1.9); miRDeep2 (v2.0.0.8); Cutadapt (v1.14); CD-HIT (v4.6.7); bowtie (v2.2.5); miranda (v3.3a); RNAhybrid (v2.2.1); DAVID; GraphPad (http://www.graphpad.com); UpSetR R package (v1.4.0);

For manuscripts utilizing custom algorithms or software that are central to the research but not yet described in published literature, software must be made available to editors and reviewers. We strongly encourage code deposition in a community repository (e.g. GitHub). See the Nature Research guidelines for submitting code & software for further information.

Data

Policy information about availability of data

All manuscripts must include a data availability statement. This statement should provide the following information, where applicable:

- Accession codes, unique identifiers, or web links for publicly available datasets
- A list of figures that have associated raw data
- A description of any restrictions on data availability

All data generated or analysed during this study are included in this published article and its supplementary information files. All genomic and transcriptomic data are publicly available for visualization via the open-access Bat1K genome browser (https://genome-public.pks.mpg.de) and for download at https://bds.mpi-cbg.de/hillerlab/Bat1KPilotProject/. In addition, the assemblies have been deposited in the NCBI database and GenomeArk (https://vgp.github.io/genomeark/). Accession numbers and BioProjects for all data deposits can be found in the supplementary information files of this article.

Field-specific reporting

Please select the one below that is the best fit for your research. If you are not sure, read the appropriate sections before making your selection.

☒ Life sciences ☐ Behavioural & social sciences ☐ Ecological, evolutionary & environmental sciences

For a reference copy of the document with all sections, see nature.com/documents/nr-reporting-summary-flat.pdf

Life sciences study design

All studies must disclose on these points even when the disclosure is negative.

Sample size	Genomes and transcriptomes were generated from a single individual per species to ensure that there were no confounds introduced into assemblies or annotations due to inter-individual differences. This is the standard in the field. Lab experiments on miRNA target regulation were replicated independently 3 times, each independent replication involved 3 independent samples. This is the same sample size that has been successfully used previously for equivalent tests published, which show significant differences.
Data exclusions	No data were excluded from the analyses.
Replication	All lab experiments were replicated 3 independent times. All attempts at replication were successful.
Randomization	Randomisation was not necessary as genomes and transcriptomes were generated from a single individual per species. Small RNA were sequenced from brain, kidney and liver tissues from a single individual per species. The protocols for genomic DNA extraction, genome sequencing, total RNA extraction, miRNA-Seq, IsoSeq, cellular reporter assays and data analysis pipelines were consistently applied to 6 bat species.
Blinding	The investigators were not blinded to the location, species and sex during sample collection for genome sequencing, Iso-seq and miRNA-Seq. Blinding was not necessary since these identifying factors were not variables in the analyses and data was generated from a single individual per species.

Reporting for specific materials, systems and methods

We require information from authors about some types of materials, experimental systems and methods used in many studies. Here, indicate whether each material, system or method listed is relevant to your study. If you are not sure if a list item applies to your research, read the appropriate section before selecting a response.

Materials & experimental systems

n/a	Involved in the study	
☒	☐	Antibodies
☐	☒	Eukaryotic cell lines
☒	☐	Palaeontology and archaeology
☐	☒	Animals and other organisms
☒	☐	Human research participants
☒	☐	Clinical data
☒	☐	Dual use research of concern

Methods

n/a	Involved in the study	
☒	☐	ChIP-seq
☒	☐	Flow cytometry
☒	☐	MRI-based neuroimaging

Eukaryotic cell lines

Policy information about cell lines

Cell line source(s)	HEK293T/17 cells used for functional assays were sourced from ATCC (American Type Culture Collection)

Authentication	Cell line was authenticated by the supplier (ATCC) via visual inspection of morphology and STR analysis.
Mycoplasma contamination	We confirm that cell lines were regularly tested for mycoplasma contamination and always tested negative.
Commonly misidentified lines (See ICLAC register)	No commonly misidentified cell lines were used.

Animals and other organisms

Policy information about studies involving animals; ARRIVE guidelines recommended for reporting animal research

Laboratory animals	This study did not involve laboratory animals.
Wild animals	A female M. myotis bat from Limerzel, France was euthanized at a bat rescue centre in 2015, and immediately dissected. A female Rhinolophus ferrumequinum bat died unexpectedly and suddenly during sampling in Bristol, United Kingdom in 2016, and was dissected immediately. A male Pipistrellus kuhlii bat was captured and dissected in Bergamo, Italy in 2017. A male Molossus molossus bat was captured and dissected in Gamboa, Panama in 2018. A male Phyllostomus discolor bat originated from a breeding colony in the Department Biology II of the Ludwig-Maximilians-University in Munich, Germany, and it was dissected in 2016. A male Rousettus aegyptiacus bat originated from a breeding colony at University of California (UC), Berkeley USA, and it was dissected in 2017.
Field-collected samples	Samples were collected from the field, as noted above, but experiments were not performed in the field, as terminal samples were collected.
Ethics oversight	Myotis myotis: All procedures were carried out in accordance with the ethical guidelines and permits (AREC-13-38-Teeling) delivered by the University College Dublin and the Préfet du Morbihan, awarded to Emma Teeling and Sébastien Puechmaille respectively. Rhinolophus ferrumequinum: All the procedures were conducted under the license (Natural England 2016-25216-SCI-SCI) issued to Gareth Jones. Pipistrellus kuhlii: The sampling procedure was carried out following all the applicable national guidelines for the care and use of animals. Sampling was done in accordance with all the relevant wildlife legislation and approved by the Ministry of Environment (Ministero della Tutela del Territorio e del Mare, Aut.Prot. N": 13040, 26/03/2014). Molossus molossus: All sampling methods were approved by the Ministerio de Ambiente de Panama (SE/ A-29-18) and by the Institutional Animal Care and Use Committee of the Smithsonian Tropical Research Institute (2017-0815-2020). Phyllostomus discolor: Approval to keep and breed the bats was issued by the Munich district veterinary office. Under German Law on Animal Protection, a special ethical approval is not needed for this procedure, but the sacrificed animal was reported to the district veterinary office. Rousettus aegyptiacus: All experimental and breeding procedures were approved by the UC Berkeley Institutional care and use committee (IACUC). All experiments involving cell lines were conducted complying with the guidelines and regulations of the biosafety office of the Radboud University (Nijmegen, The Netherlands), under Dutch law.

Note that full information on the approval of the study protocol must also be provided in the manuscript.
