# Supplementary material for: Six reference-quality genomes reveal evolution of bat adaptations
Source: Nature. 2020 Jul 22;583(7817):578–84. doi: 10.1038/s41586-020-2486-3 (PMC8075899; doi:10.1038/s41586-020-2486-3)

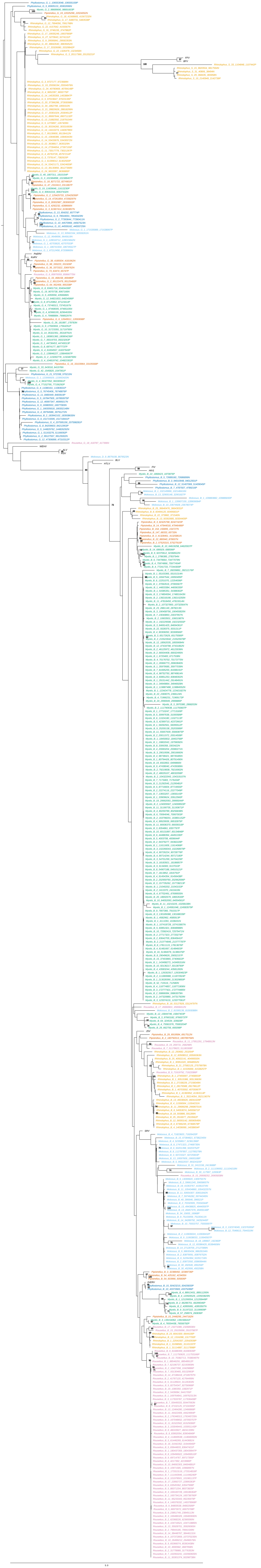

Supplement: Supplementary file 3 — | Reconstructed phylogenetic tree of viral pol sequences found in six species of bats Each species used in the ERV search is marked with a colour: Phyllostomus (navy blue), Myotis (green), Pipistrellus (orange), Rhinolophus (yellow), Molossus (light blue), Rousettus (pink) and reference sequences (black). Bootstrap values are shown where the values are ≥ 70%. The tips of the phylogeny are labelled with the species name, position in the reference genomes and the direction of the sequences (N- negative/P- positive). [file 41586_2020_2486_MOESM3_ESM.pdf]
