# Supplementary material for: Six reference-quality genomes reveal evolution of bat adaptations
Source: Nature. 2020 Jul 22;583(7817):578–84. doi: 10.1038/s41586-020-2486-3 (PMC8075899; doi:10.1038/s41586-020-2486-3)
Supplement: Supplementary file 7 — Predicted 3D protein structures of candidate genes. [file 41586_2020_2486_MOESM7_ESM.zip › Data_S3/Bat_Protein_Structures/AZGP1/AZGP1_S511107_results/lscore.html]

I-TASSER results


[Back to the Results Page]
  
  

# Top five models with local structure error profiles

The local accuracy is defined as the distance deviation (in Angstrom) between residue positions
in the model and the native structure.
Since the native structure is unknown, the distance errors in the following plots
are estimated by
ResQ
using support vector regression that makes
use of the coverage of threading alignment, divergence of I-TASSER simulation decoys, and
sequence-based secondary structure and solvent accessibility predictions.
The numerical data of the ResQ prediction are listed in this file: lscore.txt.

More details of ResQ and the local structure error prediction can be found at   
J Yang, Y Wang, Y Zhang. ResQ: An approach to unified estimation of B-factor and residue-specific error in protein structure prediction, Journal of Molecular Biology, 428: 693-701 (2016).
